# Supplementary material for: Breeding displacement in gray wolves (Canis lupus): Three males usurp breeding position and pup rearing from a neighboring pack in Yellowstone National Park
Source: PLoS One. 2022 Nov 30;17(11):e0256618. doi: 10.1371/journal.pone.0256618 (PMC9710779; doi:10.1371/journal.pone.0256618)
Supplement: S1 File — (DOCX) [file pone.0256618.s001.docx]

WOLF OBSERVATION NOTES

July 2016

by Rick McIntyre**

As of 6/1 the Junction Butte Pack still numbers ten adults and nine pups. The Lamar Canyon has four adults.

Unless I note otherwise the Junction wolves were at the den area every day in June. I will note some of the sightings I had there.

On 6/1 Kira did a flight and saw that the Eight Mile wolves had moved their pups to Gardiner’s Hole. A new bison carcass was seen just east of the Lamar den forest.

The four Lamar adults were at that carcass on the 2^nd^. At Slough a gray yearling was digging at the sage den. In the evening 911 reportedly went there and pups followed him. Some of the pups went into the sage den.

I saw most of the Junction pups at the sage den on 6/3, but one gray pup was still at the natal den. A gray yearling came in with the head of an elk calf and gave it to a pup.

On the 4^th^ we saw 1015 and the black female do double scent marks. 1013 and a total of five blacks were in the Mollie’s group. They were at or near a new bison carcass at Amethyst Bench. The unknown black yearling was north of Dorothy’s. The Mollie’s wolves did a lot of howling and the black howled back. I went to Slough and the Junctions also did a lot of howling. I think that both packs and the lone black were hearing each other.

In the evening the four Lamars came out and both 965 and 993 marked over 926's FLU. Later the Mollie’s wolves chased the Lamars to the east. 826, Small T, and 965 ran across the road to the north near the Picnic Area. The Mollie’s continued east after 993. He reportedly ran across the road at 21's crossing. The Mollie’s turned around west of the confluence.

I had four black Mollie’s and 1013 south of Hubbard Hill on the 5^th^. 1015 and the black female did a double scent mark. I saw five black pups at Slough. I had the two Lamar males howling to the north in Soda Butte Valley and the two females howling from the south. Later the two females reportedly crossed to the north.

We had the usual sightings at Slough on the 6^th^.

People reported seeing all five black pups at Slough on the 7^th^. We have been wondering if the fifth pup might have been lost. The pups nursed that day and that was the last time we saw nursing.

On the 8^th^ I saw all nine pups at Slough.

I hear that a collared gray and an uncollared gray were seen at the coyote den near Lamar Canyon West early on the 9^th^. I saw a yearling coming back from that area and others saw 911 on that route earlier. Two ravens have been hanging out in the den area and often are perched below the pups at the sage den. The pups generally ignore them. I saw five black pups.

On the 10^th^ 779 and a black adult were seen on K Meadow. They later went downhill and were lost. Dan did a flight and saw 926 and 965 on Druid Peak. He had seven pups with the Eight Mile wolves in Gardiner Hole. Nine Junction wolves were reportedly seen in the evening.

I had 926 and Small T going back toward the den forest from the west early on the 11^th^. I saw nine pups at Slough, but not all at once. They were spread out and in different locations.

On the 12^th^ I saw 907 nursing pups for about two minutes. She and three yearlings led the pups up to the diagonal forest and the pups explored that area. Later 907 brought them back to the den area. I had a count of nine pups. We saw 911 and 969 south of Dorothy’s. In the evening the pups went south of the burnt stump and came back to the den area. People saw five black pups that evening.

I saw 907 come into the den area on the 13^th^. Yearlings and pups chased her and she did not regurgitate to the pups until the yearlings dropped out. Later 969 came in and did about the same. A black pup seemed to cache some meat. I had what seemed like five black pups. I did not see five black pups after that count in the days to come. In the evening five pups went to the diagonal forest by themselves and came back. 969's pups are now likely two months old.

As of the 14^th^, we have only been seeing four black pups and four gray pups. One black pup pulled 907's tail.

On the 15^th^ it was raining in the morning, but we saw wolves at the Slough den. A gray female yearling has a bad limp on her hind left leg. In the evening I had three of the Lamar wolves at the Norris RS. After I leave people saw all four Lamars going toward Cache Creek.

Most of the adults left on a hunt on the morning of the 16^th^. A black pup seemed to cache some meat. The hunting adults were not back by the evening.

I saw 907 chasing 12 bighorn rams on the 17th. 890 came back from the west that morning. 907 is often visible in the den area, but we do not see 969 there as much. I get her signal there often, but do not always see her there. We have been having a lot of howling by the adults and pups. A bison was hit by a car last evening and I see that it was dragged off to the north.

On the 18^th^ Doug had 890 near that bison carcass. A fair amount was eaten, and it was dragged a bit to the north.

The adults and pups went up to the Diagonal Forest and came back on the morning of 6/19. 907's pups are now likely two months old.

I had all ten adults and eight pups at Slough on the 20^th^. We now feel that the fifth black pup has been lost. Six of the adults went from the den area to the diagonal forest and later the other four adults and five pups followed that route. In the evening nine adults left the den area and went downhill to the south, then turned around and went out of sight toward the campground.

On the 21^st^ I got signals from 926 and 965 in the general den area. We later learned that 993's GPS points indicated that he had localized that day at lower Cache Creek and that he was likely injured at that time. His collar sent out a mortality signal from that area on the evening of 6/26.

I got 926 and 965 in the den area again on the 22^nd^. In the evening at Slough 907, two yearlings and seven pups went south on the den ridge and went out of sight just north of aspen pass. They later went back north. Pups tried to nurse on 907 but failed. On that day I had my 90,000^th^ Yellowstone wolf sighting.

907 was the only collared wolf at the den on the 23^rd^. Two yearlings and all eight pups were also there.

A bison bull came into the Junction den area on the 24^th^ and pups ran from it. The bull chases a yearling and later two yearlings chased him. Doug did a flight and got signals from 926 in Soda Butte Valley.

I got signals from 926 in the den area on the 25^th^. At Slough three pups went to the diagonal forest by themselves and came back on a different route. 969 was at the den. She is usually not there. 907 is in the den area much more often than 969.

On 6/26 I see a run over snowshoe hare on the road west of Upper Baronett.

We had 926, 965, and Small T on an elk calf carcass north of Coyote that morning. The gray male Junction yearling went with five pups to the area just north of the horizontal forest, then they went back to the den area. In the evening nine adults went downhill from the den area into the lion meadow, then turned back uphill and went into the diagonal forest.

At Slough the limping gray yearling interacted with three black pups that passed through the den area. I got 926 and 965 in Round Prairie.

In early morning on the 28^th^ 890 and 911 went north toward the area west of the campground. The gray male yearling brought several pieces of meat into the den area from the east. He put one piece in the sage den. Other people saw 926, 965, and Small T south of Footbridge. 926 did a lot of flirting with 965 as they headed south. Later that day Wolf Project people hiked out to Cache Creek and found 993's body. It looked like he had been trampled but lived on for a few days. His marrow was poor.

At Slough on the 29^th^ six pups led to the diagonal forest and two yearlings followed them. They all later went back to the den area and the pups slipped into the den.

I got good signals from 965 at Round Prairie that day.

On the 30^th^ I had three black pups go to the meadow below the diagonal forest then come back to the den area.

I checked back on nursing data and found that I last saw 969 nursing pups on 5/27. Her pups would have been six weeks and two days old at that time and 907 pups would have been six days younger. She walked away from pups trying to nurse on 6/1 and was not seen nursing after that.

907 seemed to nurse a number of pups for about two minutes on 6/12 and may have nursed one pup for 45 seconds on 6/22. She walked away from pups trying to nurse after that. When she did the definite nursing on the 12^th^ 969's pups were three days short of nine weeks old and her own pups would have been two days short of eight weeks old.

On the evening of 6/2 911 lead the pups to the sage den.

The last sighting of the fifth black pup seemed to be on the morning of 6/13.

July 1:

It is 42 when I leave at 0438.

I do not get any signals in Lamar.

I arrive at Slough at 0520 and just get 994.

At 0535 I see the black male yearling in the dead tree meadow. He sniffs around the marsh and is probably getting the scent of the pups. Then he looks around.

The black goes east and continues to sniff around as he heads toward the lower part of the diagonal meadow. He probably is on the scent trail of the pups. Soon the yearling is in the meadow below the diagonal forest where the pups had been last evening. Some bison chase him, and he continues on to the northeast.

At 0602 he is at the creek north of the third lot. The black sniffs at the site in the willows where he had carried part of a bull elk carcass that he took out of the creek last week. After that he wades part way into the creek and drinks.

We see him going north and we lose him behind a hill west of the creek. He was going northeast at that time.

I see a gray pup in the den area at 0633. 994 is downhill from the eastern trees.

A gray yearling goes downhill from the eastern trees at 0737. Then I see that both uncollared gray female yearlings are going east from the eastern trees. One of the stops, sits up and howls at 0744.

994 howls from behind the two dead trees. I see 907 bedded near him.

A gray pup is at the sage den.

At 0806 the black male is coming back to the eastern trees.

I see a black pup with 907 and have seen another black pup in the area.

I only saw three pups this morning: two blacks and one gray.

I only get 994 at 0945.

I go east and do not get any signals in Lamar.

It is 67 when I leave at 1734.

I do not get any signals in Lamar.

The signs and barriers are up in the Hitching Post lot.

I start looking at Slough at 1816 and see a black pup going downhill toward the dead tree meadow.

I get signals from 969 and 994.

Two black pups are playing behind the two dead trees.

I soon have two black pups and three gray pups in the meadow.

Two gray adults are now visible. Later I see that the collared one is 994 and the other is an uncollared female. She is in the meadow and he is bedded under the eastern trees.

One gray pup has a stick or white bone.

Another black pup is under the eastern trees. The other two black pups are now by the logs. I see the fourth black pup.

Two black pups go to the bedded gray female. She tilts back and paws at them. The two blacks run to a gray pup. The yearling gets up and I see that it is the limping one.

She goes with some of the pups to the south, toward the burnt stump. Three blacks and three grays are with her and she plays with them as they travel. Two black pups are leading. The yearling romps and interacts with them.

The gray male yearling is now also with them. I now see that 907 and 994 are also in that area.

The lead wolves are now south of the stump. They greet each other. 907 is north of the stump and all the others are now south of the stump.

907 joins the other three gray adults and they socialize.

The gray male runs south, and the six pups follow in single file at a run. They are soon to the upper left of the small diagonal forest. The limping gray female runs to catch up with them. A gray pup runs all out to catch up as well.

I lose them behind a knoll there at 1940.

I soon pick them up to the upper left of the two diagonal trees. That is the furthest south the pups have been. They turn back at 1944 and head north. A gray pup is leading back toward the den.

Three of the adults veer uphill to the upper right. The gray male is not with them. The pups continue north and some of them are on different routes than other pups.

Some of the adults come back down to them. 907 is now leading back toward the stump at a run. The limping female is playing with some of the pups.

A black pup is on the ground and a gray pup stands over it and lunges at it. The black lunges back at it.

The pups are now approaching the sage den. A black pup runs fast to catch up.

The black female yearling is coming down the trail from the eastern trees. Pups run to her. They all go east toward the broken tree stump at the east end of the meadow.

I now have five gray adults. The drab gray yearling is there, and the pups run to her.

At 2009 they are in the sage in that area.

The limping gray yearling is still playing with pups.

At 2028 I just get 969 and 994.

I get weak signals from 926 at Hitching Post at 2051.

July 2:

It is 43 when I leave at 0436.

I get a good signal from 926 east of Footbridge at 0503. I do not get 965. I lose her at Picnic Area.

I go to Slough and at 0534 see a gray adult, two black pups and a gray pup going south toward the burnt stump. This is the limping gray female.

I see the black male yearling with a gray pup and they are catching up with the others.

The wolves are on a route downhill from the burnt stump.

The gray female is chasing the pups. Two black pups run back and forth as she goes after them. I now have two gray pups there with the two black pups.

The pups explore as they go south. The gray female follows, but I have lost the black male.

I now have 994 with them, the drab female yearling and see a third and fourth black pup there. I only have two gray pups.

I get a loud signal from 969. 890 and 994 are weaker.

The limping gray is now leading north at run. She is soon back in the dead tree meadow. I soon see four black pups and her on the grassy hill above the meadow.

I spot 969 coming out of the gully by the sage den. A gray pup is at the den.

The black male yearling comes in from the south and passes 969. The pups run to him and greet him in the sage below the den. The gray pup at the den stays there and looks downhill.

I also see the black female yearling bedded in the dead tree meadow. Pups and other adults are all interacting there. A gray yearling somehow ends up cross way on the back of one of the black yearlings and runs it for a few moments before slipping off.

Suddenly two black pups there run off in different directions like they are scared of something.

I see 969 going toward the burnt stump. 994 and the drab uncollared gray are with her. All three grays bed up by the stump.

Two black pups and a gray pup are at the sage den. They run downhill.

I soon have the two black pups and two gray pups in the meadow with the limping gray female.

Two of the gray adults by the stump are up and going uphill. 994 is apart from them and he stalks toward some bighorn rams. They run off and he watches them.

I lose 969 in the pass in that area at 0827.

The drab yearling goes toward the diagonal forest and I lose her.

I go back to the lot and get 890. I see him coming in from the south. Two gray adults follow him uphill. He did not do a regurgitation. I lose him up at the eastern trees.

I see the male black yearling in the meadow.

We hear that 926 and 965 are visible in the Institute area. I go that way at 0855.

I get 926 and 965 as I go by the Institute. Emile reports that he had 926 south of the road in that area. She crossed the road east of Hubbard Hill to the north and joined 965. They chased three or four coyote pups and did not get one. Four adult coyotes harassed the wolves.

I stop at Hubbard Hill and at 0914 see 926 and 965 to the northwest. Coyote are giving the alarm call out of sight. The two wolves go west, and I lose them.

I go to the Institute at 0920. I do not see the wolves again. I spot four adult coyotes and they are acting like they are searching for their pups. Three go out of sight into a gully and the other one stays on a ridge and looks around.

I do not get the Lamar signals there at 0943.

I go behind the buildings and get faint signals from 926 at 0948.

965 is weak at Hubbard at 0959.

Both later are weak at the Institute.

I do not get either signal when I go east at 1023.

It is 75 when I leave at 1723.

I get variable signals from 965 from the Institute through Dorothy’s and do not get 926.

I arrive at Slough and at 1806 see pups by the logs. They go to the eastern trees and I see all eight there.

I get signals from 890 and 969.

I see the black female yearling.

A black pup is in the dead tree meadow and is going north.

We lose the pups, then see some of them nearing the diagonal forest. I see three blacks and three grays near the gap. I then spot the black male yearling and 907 going through the lawn downhill from the gap at 1857. I lose them in the trees. The pups must have been following the two yearlings to the forest, then got distracted.

The pups are exploring and not following the two yearlings. They move toward the lawn and I see two blacks and a gray chasing each other there. They run right and I lose them.

907 and the black yearling are out behind the horizontal forest and are going north at 1917. 907 is leading. I lose them going toward the knob near the yellow grass meadow.

I hear that all three Wapiti adults were at the bison carcass this morning at 0800.

I see 890 at the right end of the eastern trees with three gray pups and black pup. 994 is also there.

At 1944 I see three black pups coming back from the diagonal forest. They pass by the crescent rock and head toward the eastern trees.

I see a black pup bedded in the dead tree meadow.

A gray adult and 890 are still at the eastern trees. The new pups run in and harassed 890.

I do a check at the lot at 2006 and get 890 and 994.

I stop at the entrance lot and get good omni signals from 926 and 965. I think they are toward the bowl or secret passage. They are weaker at 2932.

I go east and get weak signals from 926 after coming out of the east end of Lamar Canyon. I do not get 965.

Both signals are weak at Coyote. She is faint at the Institute.

July 3:

It is 36 when I leave at 0439.

I get weak signals from 926 in Soda Butte Valley and lose her after passing Hitching Post.

It is now too dark to see at 0500.

I get signals from 911 and 969 at Slough at 0525.

I see a back pup east of the western trees.

I soon have four black pups and a gray pup in the dead tree meadow. A GPS collared gray and a black yearling are also there. One black pup is playing with the gray adult.

One black pup lifts its head and howls.

A number of pups climb up on a boulder.

I see a second gray adult bedded in the meadow.

I hear group howling that including higher pitched yip yapping from the pups. The black male is up and howling. It is 0548.

A second gray pup runs down to the meadow and I see a third gray at the sage den.

A black pup goes to the sage den and the gray pup there. Another black pup and a gray pup also go there.

Later three black pups and three gray pups play together on the grassy slope behind the two dead trees.

A black pup from that group goes on the trail up to the eastern trees. The other two blacks and the three grays follow the first pup. The gray pup at the den then goes that way. All go out of sight east of the eastern trees. I have seen all eight pups.

I see 907 in the meadow. She was one of the two grays I had seen earlier. I hear that the other gray adult was a yearling and that it went out of sight.

At 0747 a black pup goes down to the spring.

I see the limping gray yearling going downhill from the eastern trees.

I have the black female yearling up between the two dead trees. A black pup is with her.

At 0734 three pups are running around in the meadow. Two get together and mouth each other.

969 comes in from the left to the meadow with what look like the hindquarters of a small fetus. The pups run to her. She puts it down, but the pups continue to run after her rather than go to the item. 969 stops and puts her head down to regurgitate.

907 gets up to the west of them. She looks at the other wolves, then beds again.

969 chases a magpie from the hindquarters.

A gray pup goes there and picks up the small hindquarters and carries it around. This may be part of a pronghorn fawn. That pup puts it down and controls it.

Two gray yearlings and the black female yearling run in from the north. The grays include the male and the limping female. They go to 969. She blocks them from the hindquarters. 969 pins the black female, then blocks and pins the other two yearlings. It is obvious that she only wants the pups to get the food.

At one point the gray male tries to crawl toward the food. She pins him.

The gray pup is eating at the site. None of the yearlings are there.

969 goes there and hoovers over the spot. Two black pups and a gray pup go to the site.

One gray yearling is on its back with its legs up in the air.

969 walks off to the east and seems to be resigned to the yearlings getting part of the meat.

A black pup greets the male yearling. The other yearlings are with the pups at the site.

969 comes back from the spring and has likely had a drink there. She looks full. I then see her going up the trail to the north.

Three pups and a gray yearling are at the meat and I can see the yearling eating beside the pups. It does not keep the pups away. I see a gray pup eating there. It is 911.

Two more gray pups run to the site. That makes three gray pups and two black pups there. The other gray yearling also comes in. The black yearling is walking around east of the site.

Later the limping gray yearling plays with the pups in the willows. She seems to get a rodent there and I see her chewing.

The drab female gray yearling comes in. The pups run to her and they greet her. The black yearling also greets her. The drab yearling goes to the meat.

I see a black pup run off from the site with a tidbit.

The drab female goes up to the eastern trees. The male gray is at the feeding site as is the limping female.

The male gray goes to the spring and drinks.

The limping gray has been doing a lot of playing with the pups.

I still have a gray pup at the site at 0946.

I get signals from 911 and 969 when I leave at 1053.

I do not get any signals in Lamar.

It is 54 and raining when I leave at 1738.

I get 926 and 965 in Soda Butte Valley at 1800 but only get him around the den.

When I park at Slough at 1825, I get 911 and 965.

At 1831 I see a bedded gray adult near the east end of the eastern trail.

A gray pup is west of the dead trees.

I see four black pups and two gray pups below the live tree under the burnt stump. They are playing.

The gray adult by the eastern trees gets up and it is the drab yearling female.

A gray pup is sitting up at the sage den. It goes in the den. That pup may have been the one near the dead trees earlier.

The limping gray yearling is with the six pups. She chases a gray pup.

A gray pup has a tidbit and two black pups chase it. The other three pups watch them.

There is thunder and the pups ignore it.

The wolves howl and we can hear them.

I now have four black pups and three gray pups there. The fourth gray pup comes out of the sage den. That pup probably is the one that seems to often be apart from the others.

The seven pups wag their tails as they gather around the limping yearling. The pup are doing a lot of playing together and running around while the fourth gray is still by itself.

The yearling chews on an antler.

The four gray pup is starting to more toward the others. They are now under the eastern trees.

The limping gray goes down toward that fourth gray and the pup hesitates on seeing her. The yearling stops and stares at it. The pup turns around and goes back toward the sage den. The yearling runs after it and chases it to the den where they greet each other. That pup seems wary and unsure of itself. I wonder if something physically is wrong such as poor eyesight. It seems smaller than the other pups and is more golden.

The yearling goes down into the gully.

The seven pups are on the alert and looking down that way. They run downhill, then stop and look downhill again. The pups are acting like they are seeing something in the gully.

The fourth pup had gone downhill from the den. It comes back up and goes into the den.

The main group of pups look west. I hear that the limping yearling went that way.

I now have two black pups and three gray pups at the sage den. Another black pup is just uphill from there. I think the fourth gray is still down in the den.

A ranger vehicle is going through Little America with its siren on. All the pups look that way in unison and move their heads as the sound moves further east. Seven pups are visible at the den now.

The drab yearling howls at 2039. She is up by the burnt stump

I see 969 in that area. She looks at the pups at the sage den.

The seven pups howl. I now have all four blacks and four grays there. They look at the adults.

I see the male yearling by the stump.

The wolves are looking in the direction of the siren.

969 and the gray male move off to the south from the stump. The drab yearling stays bedded there. It is 2043. The gray male leads and he does a LFU.

Two black pups run down into the gully and others look that way. An adult is probably down there.

The drab yearling by the stump howls. I see the limping gray female there as well. Perhaps those two female are not all that interested in hunting while 969 and the male yearling just left on a hunt. Or perhaps they are going to a carcass and the two females are just not hungry.

I only get 969 when I leave at 2104.

I get weak signals from 926 from the Institute through Trout Lake.

July 4:

It is 42 when I leave at 0438.

I get a good signal from 926 from Soda Butte Valley through the den area. I do not get 965.

There is thick fog in the valley.

I can see the den area when I get to Slough.

I start looking at 0524. At 0540 I see a black pup east of the single tree at the north end of the western trees. Doug has seen other pups there.

I spot the black male yearling.

At 0552 we see 890 going south near the Simpson Tree to the west. The limping gray female is south of him and she is sniffing around as she moves south. I lose her south of the southern round tree.

The black male is looking into the sage den. A gray pup comes out.

Lizzie got good signals from 994 in the Peregrine Hills area.

The black male is gone but I see a black pup where he had been.

We get a report of a black wolf swimming the river to the north from the aspen area.

The black male runs west and meets up with a gray adult. I see a number of wolves there with their heads down. That is the area where I had seen the black pup earlier. I now see two black pups, the drab yearling and the limping gray yearling. A black pup leads north.

We hear that a collared gray was seen to the south of the road in Little America.

The group of wolves is going north. I now have four black pups there.

I see the black male. A gray pup is there. It is 0615. I see a second gray pup.

Three black pups and a gray pup are going south and are now near the small diagonal forest.

The limping gray yearling looks into the sage den, then sniffs around uphill. She follows a scent trail on the route to the eastern trees and I lose her up there.

The four pups that went south are running back to the den area.

The small gray pup is out at the sage den. It sits up and looks down toward the gully.

The three black pups are running back, and the shy gray pup is looking that way. The limping gray is now in the dead tree meadow. She wags her tail.

The gray male yearling is now with the pups. He and the limping gray are greeting the returning pups. They are southwest of the sage den. The wolves have their heads down. We had wolves at that site earlier and I wonder if there had been a regurgitation there.

A gray yearling leads up toward the burnt stump. Two black pups and three gray pups follow. I think the shy gray pup has joined them. The gray male is limping on his hind left leg, the same leg that the gray female limps on.

The small gray pup goes to a bigger gray pup and greets it.

I do not see any pups at the den.

The gray male is romping around with the pups and at one point ran sideways ahead of them so that he could look back at them.

At 0649 I see the black female yearling coming up the lion meadow with what looks like an elk leg with little meat on it. The bone is thick. She comes into the dead three meadow and a gray adult follows her as she goes below the sage den. That is the drab gray yearling.

I have sene all three gray uncollared yearlings and both black yearlings, along with 890.

I later see the drab gray with a gray pup near the burnt stump.

Three gray pups and a black pup are going south from the burnt stump at 0705. The three grays run back to the limping gray like they have not seen her for a while but had been with her five minutes ago. The black pup does not go to her, so she goes to it.

I have to go back to the car. When I get back, I hear that 890 had come in and regurgitated to the pups. I see him southwest of the sage den.

I hear that the three Lamar wolves were seen south of Footbridge at 0715 and later were lost going into the trees at Chalcedony Creek. That was at 0745. 965 did two RLUs.

We see the black male yearling to the west, north of the Simpson Tree. He is carrying a large leg that looks like it has bison fur on it. It is 807. We lose him in the hills and gullies south of the den ridge. Later he comes out without the leg.

I go to the car and at 0811 get loud signals from 890 and weak ones from 994. I go east at 0821.

Lizzie is ahead of me and she is getting 926 at the confluence. People are above the confluence and have not seen the wolves.

I get weak signals from 926 at Dorothy’s. I do not get her at Trash Can.

I go back and around 0812 hear that the black male has gone on the trail to the west from the southern round tree. We think he is heading back to a carcass.

I get 890 and 969 as I pass Slough. She was weak. I also get weak signals from her at Boulder. We see the black male going north toward the ridge southeast of the conifer/aspen forest at 0955. Doug had him come out of a gully north of the river and he had some meat in his mouth.

We lose him. People at Slough see him come out of the conifer/aspen forest.

I do not get any signals when I go east from Boulder at 1007.

I go back to Slough and see a black pup at the sage den at 1022. It goes into the den.

Another black pup comes down to the dead tree meadow.

969 and 890 are weak here.

A gray pup is in the meadow.

I go east at 1049.

I do not get any signals in Lamar.

It is 69 when I leave at 1732.

I do not get any signals in Lamar.

I hear that 890, a black adult, and pups are visible in the den area.

At 1814 I see the gray male going up to a high pass on the den ridge, then lose him over the top.

The black male yearling and the black female are in the dead tree meadow. I also see a gray uncollared yearling in the meadow and black pup.

I only get 890 at 1833.

I now have three black pups and four gray pups running around in the meadow.

The pups go to the gray yearling. They greet her and follow her around. The pups go to a log and play around there. The yearling stays with them. The fourth black pup joins them. This is the drab yearling.

She is different from the gray I saw in the meadow earlier. That gray was probably the third gray yearling, the limping one.

The drab yearling is doing a lot of playing with the pups. The pups are mostly playing on a log. The yearling walks off to the east and beds.

Melba calls to say she had seven people in the Wapiti RS. No wolves were visible at the time. The alpha were seen earlier.

The black female is with the pups at the log. They are playing vigorously by themselves now. It is 1925.

At 1928 890 comes in from the western trees and heads toward the sage den. He stops and looks that way. The pups are in the meadow and a low ridge is probably blocking his view of them.

890 stops several times to look around for the pups. He goes to the sage den, looks in, then continues east.

890 goes down the grassy slope to the meadow. A black yearling gets up and sees him. That black, a gray yearling, and the pups run to him. He does not feed them. 890 goes east and beds after drinking at the spring.

Five of the pups play around a rock in the meadow.

890 is watching the pups from the east.

One black pup sees another back pup about 100 yards away and romps over to it.

As pups play on a rock if one cannot get up on one side it goes to a different spot and gets to the top that way.

I only get 890 at 2008.

I go east and do not get any Lamar signals.

I later hear that after I left Slough a gray came in at 2000 with a piece of meat and wolves fed on it.

July 5:

It is 42 when I leave at 0438.

I do not get any signals in Lamar.

I do a check at Slough at 0518 and get 890, 969, and 994.

At 0532 I see 890 going south in the lower part of the lion meadow. He beds downhill from the leaning conifer.

I see a black yearling bedded south of him. The black gets up and I see that he has an elk leg.

I hear howling from that area.

The yearling is carrying the leg north.

907 is also in that area and she is going toward that yearling who is now bedded and eating on the leg. 890 follows 907. She goes to the yearling while 890 bypasses him to the west. She wags her tail at the black as he eats, then beds near him. He is guarding the leg. 907 is respecting his right of ownership.

A black pup is east of the western trees and it does a pounce.

A black pup is sniffing around the eastern trees.

The first black pup runs to the sage den. It stands there on alert, then runs down into the gully.

At 0632 I see the other black yearling with 890. That looks like the female. The other black must be the male. He still has the leg.

I see 907 limping south along the west bank of the creek. I see 994 in that area as well.

At 0643 I see another gray adult bedded by the creek just north of there.

The black male is up and carrying the leg north.

I see 907 and the black female south of the southern round tree and going further south.

I have two black pups near the sage den. A gray adult is near them. I also see a gray pup there.

994 is now up in the den area and he does a LFU.

I now have three black pups and a gray pup. They meet up with 994 and he may be doing a regurgitation to them. 969 and an uncollared gray yearling are also there. They are east of the western trees. The gray is the limping female yearling.

Three black pups and a gray pup follow 969 south. The limping yearling and the drab gray female follow. They are going on a route that takes them below the small diagonal forest.

The black male is bedded in the lion meadow with the leg.

At one point 969 bears her teeth at the pups. She continues to lead south and is just slightly above the flats at the base of the ridge.

They are now east of aspen pass and the diagonal tree. That is the furthest south the pups have been. The group is at the very lower part of the slope and close to the flats.

969 beds slightly behind a sage and the pups miss seeing her as they continue south. She gets up and follows. It is 0719.

The pups are with the drab female and are sniffing around a site. 969 and the limping gray come, and the three adults come together. It looks like drab pinned the limping one, but it may have just been play.

They have a big group howl at 0722.

969 continues south and the pups follow. They stop to sniff at a site with a yearling. 969 beds on a hill just south of them. She watches the pups sniff around and dig at a dirt patch. Then she goes to the pups.

The two yearlings are going back to the north on a different route from the one they took here, and the pups are following them. 969 follows last in line. It is 0749.

I hear howls and see that 969 is howling. The rest are joining in. The pups are doing the yip yapping call.

The two yearlings continue to lead north and the four pups and 969 follow. They come out above the two diagonal trees.

The drag gray leads as they pass the small diagonal forest at 0759.

Drab and 969 are now rushing to reach the den and are now almost at the gully below the sage den.

I see them greeting 911 to the east of the dead trees. A new gray pup is there. All the wolves are wagging their tails, including 911. I see the gray male and 994.

A black pup runs north from there. The limping yearling comes in.

I now have four gray pups in that area.

A gray pup follows a scent trail to the sage den and goes in it.

I see the fourth black pup running downhill from the eastern trees.

The last pup comes in from the south. It is 0808.

The gray comes out of the sage den and I see that it is the limping female. She goes to the eastern trees.

I hear that the black male brought the leg to the dead tree meadow and dropped it there.

A lot of adults come into the meadow with 911 leading. 994 does a LFU. Pups run to 911 and lick his face. He runs forward and puts his head down. Yearlings run to him. The wolves are eating there. A gray pup and two more black pups run there. Another black pup watches that area from the north, then runs in at a really fast pace.

Wolves are harassing 911 for another feeding.

I see a second collard gray there.

All eight pups are there. They follow 911.

969 comes in from the west.

Later, at 0837, three gray pups and a black pup run downhill to the upper part of the lion meadow. Since they are so active, I wonder if they were the ones not in the group that went south so far.

The veer into the sage to the east of the single aspen, then come back out to the meadow. They are playing and running around with a lot of energy.

As I leave Slough at 0914, I get 969 and 994.

I go west and do not get 890 through aspen. I turn around and go east.

I do not get any signals in Lamar.

It is 65 when I leave at 1736.

I do not get any signals in Lamar.

I start looking at Slough at 1817 and see a black yearling, a gray pup and a black pup near the two dead trees. The black yearling beds there. I see the other black yearling nearby.

I hear that one of the black yearlings and a GPS gray just came in from the west prior to my arrival. The drab gray ran out to greet them.

I now have two gray pups and three black pups.

890 is near the eastern trees.

The pups look intently to the east and I see 969 coming in from that direction. Two black pups and three gray pups run to her and greet her. She continues on without feeding them.

Later two pups wrestle in the meadow.

A collared gray adult with a dark back greets the black yearling left of the left dead tree. That collared gray and a black pup go east to the other bedded black yearling.

I see a cluster of wolves left of the left tree and they are surrounding 911. Several had run in to that site. 969 and a lot of pups are now there. It looks like 911 has done a regurgitation there. Other wolves seem to be feeding at that site in the sage.

Another gray adult is east of there.

I have all four gray pups there now.

One of the gray adults is 994. He goes right. It is 1942. 994 runs back.

The wolves are trying to get another regurgitation from 911. He puts his head down and does one.

The black female yearling is there but the black male is off to the east.

994 and the black female move off from 911 and the site.

The pups are clustered at the regurgitation site with their heads down.

I have all four black pups there, along with the four gray pups.

911 beds close to the pups at that site. He gets up. The pups run after him and he does a third regurgitation.

A black yearling and a gray yearling are there.

911 runs off to the east and the pups chase after him. He stops and does a fourth regurgitation. After that he runs off and I lose him over the hill to the north of the meadow.

Some of the pups and yearlings are at the third site and others are at the fourth site.

The drab yearling is there.

Later the drab gray plays with the pups.

I have seen seven adults, not 907 or the other two gray yearlings.

994, the black female yearling and drab run together and the black female gets on top of drab. I am not sure if that is play or dominance. 994 is not involved.

I go east at 2016.

I do not get any signals through Hitching Post at 2030.

July 6:

It is 41 when I leave at 0441.

I get weak signals from 926 in Soda Butte Valley and in the den area.

When I arrive at Slough at 0521, I get 890, 969, and 994.

I look from the lot and at 0522 see at least two blacks and a gray in the sage southwest of the sage den. They may be feeding on something there.

I go out to the hill and at 0529 see 890 coming in from the lower right. He sniffs around below the two dead trees.

I see both black yearlings, a collared gray adult, another gray adult, and a black pup in that area. The black male beds.

The wolves howl at 0536.

The limping gray yearling comes down the trail from the eastern trees. I also see the drab gray female yearling.

There is more howling at 0548.

I have seen all four black pups.

I hear faint howling at 0620.

The drab gray is at the eastern trees. She looks northeast and goes that way. I see two black pups romping around the crescent rock. A collared gray is out in front of them and is heading toward the diagonal forest. It may be 994.

A black pup is going toward the upper part of the diagonal forest.

I spot 890 going north, north of the diagonal forest. 969 is out in front of him. She howls, then follows a scent trail north. It is 0633.

I lose those wolves. They were uphill from the horizontal forest.

Later I see a lot of wolves north of the horizontal forest. A collared gray is leading. A bit later a black pup leads.

I soon have 890, 969, 994,the black male yearling, the black female yearling, the drab gray yearling, the limping gray yearling, four black pups, and three gray pups.

At 0645 they are to the upper right of the horizontal forest.

A black pup is still leading, and it is following a scent trail.

Now 890 is leading. He is well ahead of the others. 890 stops and looks back. Then he beds. They are near where the line of willows and aspens north of the horizontal forest veer off to the northwest.

890 moves further north at a slow pace. He stops and looks back. Many of the others are bedded.

Later 969 turns around and goes back to the south. The yearlings and pups follow her. It is 0706.

The wolves howl.

At times a black pup leads back to the south. It is going on a different route than the one they took to the north. On the way the black male plays with the pups and chases them.

At 0730 I see the fourth gray pup at the sage den. It is probably the small one that often stays back.

We lose the main group heading into the diagonal forest. At 0737 I see a black pup in the lawn in that forest.

Soon the wolves are going past the crescent rock and heading toward the eastern trees.

I see some of them just right of the eastern trees at 0739.

A minute later two gray pups are heading toward the sage den. The gray pup at the den is sleeping.

A black yearling and a black pup are also approaching the sage den. The first gray pup gets up and goes into the den.

The black yearling looks in the den. The drab yearling is there. The black yearling turns around and goes downhill from the den.

More pups come in. I have seen all four gray pups there. Pups go in and out of the den. At least three black pups are there or have gone downhill. It is 0751.

I go back to the car at 0809 and get signals from 890 and 969.

The black male goes to the meadow and drinks.

I go east at 0853.

I get loud signals from 926 and 969 toward the west end of the ledge trail from Picnic Area at 0919.

I hear that around 0845 all three Lamar wolves were seen up there.

I still get loud signals when I leave to the east at 1009. I did not see them.

I do not get any signals at Hitching Post at 1045.

I recall that a gray lingered at the natal den for a number of days after the other pups moved to the sage den. That probably is the same small gray pup that has stayed behind at the sage den when the other pups have wandered off.

It is 52 and raining when I leave at 1736.

At 1808 I get a good signal from 926 between Trash Can and Picnic Area. I do not get 965. Her signal soon drops off.

I go to Slough and see a black adult bedded in front of the west end of the eastern trees at 1833. A black is nearby. The black yearling gets up and walks off to the west and I lose it. I then see it heading to the sage den. The black looks in the den, then rushes downhill.

I see four black pups and two gray pups in the area.

969 comes in from the east. The pups run to her and greet her. She continues west and they chase her into the gully. It is 1844.

A gray pup is at the sage den. That would be the third gray pup/.

I see a gray yearling there as well.

Some of the pups go down to the meadow. A gray pup chases a black pup there.

969 is walking around the grassy hill above the meadow and sniffing around. She beds there.

The pups are bounding around in the meadow. As a black chases a black it looks like it grabbed the other pup’s tail. That pup stops and turns back to the first black.

I see 907 going into the gully. 969 had gotten up to look at her.

A lot of the pups are a log in the meadow. I see them biting each other’s fur.

I hear from Bill that at 0839 this morning 926 was seen crossing the road to the north. The other two Lamar wolves were also seen in that area. At one point all three were on the road.

A gray yearling is standing under the eastern trees.

The drab yearling comes into the area.

969 is still bedded above the meadow. She howls at 1938. A gray pup joins her. She continues to howl. A gray yearling under the eastern trees howls.

I go east and hear that the three Lamar wolves were interacting with coyotes east of the Institute.

I go there and get good signals from 926 and 926. At 2019 I see the three wolves to the north of the YES lot. Coyotes are calling out east of there.

I lose the wolves, then see 926 going northwest through the rolling hills at 2023.

At 2030 965 is weak and I do not get 926. I go east.

I later hear that visitors told Ranger Rebecca in Hayden that they saw seven Mollie’s and that most of them were black.

July 7:

It is 38 when I leave at 0440.

I do not get any signals in Lamar. It is foggy.

The Slough den area is mostly visible at 0527. I get signals from 890, 994, and 994.

I see one of the gray yearlings bedded under the eastern trees at 0533.

I see a black pup on the hill east of the eastern trees at 0553. I lose it going up toward the diagonal forest. Doug had seen some adults and other pups going that way, but they are out of sight now. He had two black adults. Two black pups and two gray pups there earlier.

At 0603 we see 890 well above the horizontal forest. He is going north.

I see 969 behind the two dead trees. She heads up toward the diagonal forest. I lose her approaching the gap there. She was following a scent trail.

Bill calls to say he just saw 926 crossing the road to the north near Dorothy’s. Other people had seen all three Lamar adults in that area earlier. All three were reportedly on the road at 0550.

I see the black female yearling going toward the gray yearling under the eastern trees at 0639.

A gray pup follows the black to the sage den at 0636. She looks in the den, then goes downhill. The gray pup beds on the sage den.

I see 907 near the dead trees and she howls at 0725.

The drab female is between the two dead trees. She goes to the den and looks in.

After she walks off a gray pup comes out of the den.

907 drinks at the spring then goes west, then north.

The black male yearling is also in the dead tree area.

At 0739 I see 969 and two pups in the lawn section of the diagonal forest. I lose them going north.

Both black yearlings are together in the meadow.

After doing a talk I see 969 with three black pups and three gray pups coming out of the gap at 0822. We lose them heading toward the eastern trees.

I see a black pup and a gray pup at the sage den. That accounts for all eight pups.

A gray pup and black pup from the main group run to the sage den. 969 is also there.

I see the drab female yearling and a black pup under the eastern trees. They go down to the meadow and I see that the drab female is limping a bit. She tries to grab the back of the back. It is 0841.

I leave Slough at 0916 and head to Gardiner.

I do not get any signals at Blacktail at 0955.

I see Melba in town, and she says she has been seeing the Wapiti alphas and the yearling in the RS after the seven hikers went through the RS. The pups were last seen on 6/30.

I go back through the North Entrance at 1133.

I leave the office at 1205.

I do not get any signals at Slough at 1249 and do not any signals in Lamar.

It is 70 when I leave at 1740.

I do not get any signals in Lamar.

I start looking at Slough at 1823. I see a gray adult going up toward the eastern trees.

I hear that two black pups and a gray pup were at the sage den earlier.

At 1830 I get three signals, all but 890.

I see the black female yearling with a black pup east of the eastern trees. The drab yearling is playing with a black pup behind the eastern trees. A third black pup is in the background of that area.

The black female goes downhill, and two black pup and two gray pups follow at 1914. The yearling and one black pup and one gray pup end up in the meadow. Two more gray pups and a black pup are going that way. Another black pup is behind the eastern trees.

There are now three gray pups and a black pup with the black yearling in the meadow. The yearling drinks at the spring.

Later I have two black pups and a gray going downhill in the lion meadow toward the single dead tree. No adults are with them. One black has a large blaze and the other black just has a small white dot on the chest. A third black pup is in the meadow uphill.

At this point on the tape I lost some of my narration. I recall that three black pups and a gray pup continued downhill and sniffed around at several bison wallows. They went a bit south of the aspen cluster in the lion meadow, sniffed around a wallow there, then moved back to the north.

I saw all eight pups, the black female, and the drab gray.

I do not get any signals in Lamar on the way in.

I get a call from Melba about Mollie’s wolves being in the Sour Creek RS area in the morning. I later heard that Rebecca had four blacks and three gray. The wolves reportedly came in from the south and headed north toward the Wapiti trailhead. No one saw them interact with the Wapiti wolves.

I later checked the GPS site for that day and saw that Mollie’s 1014 was south of the point of trees at 0530 and north of the point of trees at 0600. After that he was further to the north and east.

The Wapiti gray yearling was seen by herself in the RS in the evening.

July 8:

I leave at 0438.

The tape recorder is still having problems and it does not record my early notes.

The first note that registers is at 0617 where I say that the gray male yearling is getting up and howling.

I have four gray pups at the den area. They look around and some run east. Now some look southeast. They may be seeing other wolves there.

I see a gray adult at the east end of the eastern trees. The pups run that way. I note that the adult is the sixth one I have seen.

969 comes out in that area and goes west. The two adults come together and the first one is 907. They do not react to each other.

There was howling earlier from the den area and to the west.

I recall that earlier I saw 890, the black male, the gray male and the gray female yearling to the west. The black female was up in the den area. All that adds up to seven adults: three blacks, 907, 969, and two yearlings.

890 is heading north and he has to circle around a bison herd. He passes by the bison wallow near the clump of aspens where the pups had been last evening. 890 does not stop to sniff around the area.

He goes further north and beds. 911 is coming up that way with a raised wagging tail. He stands over 890 as 890 rolls on the ground. The black male joins them. 890 gets up and all three males to north at 0653.

911 would be the fifth gray adult. I have not seen the limping gray yearling or 994.

890 is leading up to the den area. 911 and the black male follow. 890 ends up west of the left dead tree. I do not see any other wolves in the den area.

I see other wolves east of the eastern trees. 890 and the black yearling joins them. There is a lot of tail wagging. 911 is going there. I lose them over the sage hill.

At 0655 I hear one wolf howling from the den area.

I see 994 romping around with a pup. He chases the pup and it runs really fast from him.

I lose some more sections of the tape.

I see 994 going north behind the crescent rock.

I have seen all eight pups and all ten adults.

I go east at 0952.

I do not get any signals through Footbridge at 1015.

I later hear that a black and gray were seen at Blacktail Ponds earlier.

It is 76 when I leave at 1745.

I do not get any signals in Lamar.

I see a black yearling behind the two dead trees at 1827.

I hear that two gray pups and two black pups came out of the sage den at 1700 and went east to the meadow. They played there for an hour. One of the female yearlings went to them. A collared gray was also seen. Also a third gray pup was spotted.

At 1830 I get weak signals from 890 and 969.

I see a gray pup east of the dead trees in the meadow. It gets a drink at the spring.

A gray adult with a dark back that may be 969 goes downhill into the gully and two black pups follow her.

I see a second gray pup going that way.

I later see a gray yearling go into the gully. It looks like drab. That was at 1833.

The black adult is bedded behind the left dead tree.

I see a third gray pup going uphill from the sage den. The two black pups that went downhill run back up to the eastern trees. I see a third black pup at the sage den.

I get signals from 890, 969, and 994.

I go east and do not get any signals through Hitching Post at 2048.

When I get home, I see that 1014 was north of the Sour Creek RS at 2200 last evening.

Laurie calls to say Larry and Linda saw the Wapiti alphas, the gray yearling, a black pup and a gray pup at the point of trees at the RS. They also saw six Mollie’s wolves in the area at 2050 in the evening: four blacks and two gray. They came out of the western trees, due west of the point of trees at the Sour Creek RS, went east and bedded. The GPS site has a point at 1900 for 1014 at Artists Point.

I later hear that Kori felt that the two grays were the Wapiti white female and a gray pup. She said that gray was a lot smaller than the other wolves. The two light ones were way ahead of the blacks and kept running while the four blacks stopped and had a rally among themselves.

Ranger Rebecca said she had seven Mollie’s wolves yesterday: four blacks and three grays.

July 9:

It is 48 when I leave at 0438.

I get weak signals from 926 at Footbridge at 0505.

I get 890, 911, and 969 at Dorothy’s at 0514.

When I do a check at Slough, I only get 994.

At 0525 I see a black pup going up the trail to the eastern trees. Then I have a total of two black pups and a gray pup romping off to the right. Doug saw three black pups there.

Two black pups go downhill to the lower left.

I now have three gray pups and those two black pups following 994 uphill.

Another gray adult is bedded between the two dead trees. I later see that it is the male yearling.

I also see the drab gray female. She is going east at 0559.

The black male yearling goes to the sage den and looks in. 994 is with him. They go down into the gully.

The gray male goes up the trail to the eastern trees and seems to be looking for the pups. He is sniffing around. I lose behind the sage covered hill to the east.

The black yearling goes up there and was following a scent trail. I wonder if they both may be going to the diagonal forest.

I then see the black yearling near the gap. He is still following a scent trail. I lose him going into the gap.

At 0621 I see the gray male again. He is going uphill to the eastern trees on the trail. Two black pups are there. The gray male comes goes north. I see more pups there. I have three gray pups and a black pup now.

I look at the diagonal forest and see a black pup coming out of the gap. It goes toward the den area and comes out to the right of the broken off tree east of the two dead trees. Then I see the black yearling and two gray pups following that route.

The gray male is back in sight. I also another gray adult and they greet each other. The other gray is 994.

994 chases the black yearling.

I see three gray pups and two black pups moving toward the den. I have two more black pups and a gray pup near the gap in the diagonal forest. That accounts for all eight pups.

The gray male is romping around with pups in the dead tree meadow.

The drab yearling is digging at a hole just downhill from that meadow at 0733.

I get a report of a sighting in Lamar. People saw two blacks and two grays to the south from Dorothy’s around 0630.

I go back to the lot and do a check at 0853. I get weak signals from 890 and 911 and better signals from 994.

I go to Dorothy’s and at 0933 get weak signals from 890 and 969.

I hear that at 0700 the three Wapiti adults and a black pup were seen by the river.

I go back to Lamar Canyon West and at 0953 get weak signals from 890 and 969. We look, but do not see anything.

I return to Dorothy’s and do not get any signals.

I go east and get faint signals from 926 at Hitching Post at 1011.

I get a report from Hayden that at 0543 three black Mollie’s wolves, including two with collars, chased the Wapiti alphas. The Wapitis split up and the three Mollie’s went after the alpha female. 755 and the yearling got together and crossed the road to the west. Later all three got back together east of the road. They crossed the road to the west at 0845. The Mollie’s were out of sight. At 1000 Mollie’s were seen well to the south.

I get another report that said the three Wapiti wolves were seen together west of the road. One of the black Mollie’s was well north of them.

I later hear that around 1600 the Wapiti alpha female, a black pup, and a gray pup were seen behind the point of trees.

It is 74 when I leave at 1721.

I do not get any signals in Lamar.

I get to Slough around 1810 and see a black pup and two gray pups in the dead tree meadow. Another black pup is downhill from the live conifer under the burnt stump. It is going south through the sage by itself. People had seen a second black pup there.

Earlier there were three black pups and three gray pups visible, but no pups.

I do not get any signals at 1819.

I see a third black pup by the sage den.

At 1912 I get weak signals from 911 and 994.

No wolves are visible.

I get the same signals at 1952.

I go east and do not get any signals in Lamar.

I later check the GPS site and saw that 1014 had a number of locations in the point of trees area from 0600 to 0700. At 0730 he was west of there and at 1000 well to the south.

July 10:

It is 49 when I leave at 0445.

I do not get any signals in Lamar.

I do a check at Slough at 0523 and get 890, 911, and 994.

At 0554 I see black female yearling at the east end of the eastern trees. Two black pups and a gray pup are with her. One black pup runs down to the meadow. Two other black pups go west toward the sage den. I see a gray pup. The black female beds.

Now two black pups are together on the grassy hill. The black female goes down to them. The two black pups run downhill and I see a third black pup there. I also see a fourth black pup.

Three of the black pups are now in the dead tree meadow.

We have thunder and it does not affect the pups.

Rain is coming so I go back to the car. It is 0602.

It lets up some and I look again from the lot at 0607.

A black pup is at the sage den and it goes inside. A gray pup comes into sight there. It and the black pups sit up at the site, then go down into the gully.

I see a second gray pup come out of the den.

One of the black pups in the meadow gets up and intently looks east. Other pups do the same. The pups run east, and I see all four grays. 994 and three black pups are running with them toward the incoming 969. They mob her but she does not do a regurgitation. She runs northwest with the others clustered around her. I lose them in the gully at 0706.

I have been seeing a bedded gray adult left of the left dead tree and Doug sees that it is 911.

The other two adults and the pups go up to the west end of the eastern trees and bed. I have all eight pups there. 907 is with 969, 994, and the black yearling there. It is 0729.

There is more thunder and the pups do not react to it.

It hails at 0738 and does again later.

The pups are clustered around the bedded 907. They must have annoyed her for she jumps up and growls at them.

I go in the car again at 0907 due to rain.

When I look again, I only have 994 visible there. The others are out of sight.

I get signals from 890, 911, and 994.

I later go east and do not get any signals through Footbridge at 0937.

I leave at 1734.

I get a message that 755 was seen on the east side of the road in Hayden near the South Rim Drive at 1215, then crossed to the west side. Ranger Rebecca saw the gray yearling cross the road near Alum Creek at 1000. No other Wapiti or Mollie’s wolves were seen.

I see what looks like 969 to the lower right of the sage den. She has a wagging tail and seems to be interacting with a pup, but I cannot see it.

She picks up a tidbit there and walks off with it. No pup follows. It is 1853.

The limping gray female runs in from the east and goes to where 969 had been. 969 comes back and I do see a black pup there. This is probably a regurgitation site.

At 1904 969 goes uphill to the far end of the eastern trees. She soon goes back downhill and returns to that spot with her head down. Then I see her romping around like she is with the pup.

The yearling goes up to the sage den and goes all the way in. I hear that a gray pup had just gone into the den ahead of her.

969 has her head up and is staring to the southeast. She howls and looks around. It is 1909.

I see the female black yearling in the lawn. I see a gray pup coming to her.

There had been a gray lump under the eastern trees and I now see that it is 907.

I have three black pups and three gray pups in the lawn. They play together and interact with the black yearling.

The black pup below the den would be the fourth black.

The yearling briefly digs at a spot there. She looks at one play group, then at another play group.

A gray adult is at the sage den. That must be the limping yearling. She does not have much of a limp now.

She goes down to where the black pup seems to be feeding on something. After getting behind the pup she strikes it on the side or rear end with a front paw. The pup turns around and lunges and snaps at her. She quickly backs up and the pup misses. The pup goes back to whatever it is feeding on and the yearling repeatedly comes back and hits it with either one front paw or at times both front paws. Each time the pup spins around and lunges and snaps and each time she moves back just far enough to avoid the bite. I am impressed that the young pup so vigorously defended its food against her. To the yearling this all seems like play, but it looks like it really annoys the pups and it is getting angry at her.

The double paw hit looks like she is pouncing on the pup.

She does not try to nip at the pup after it lunges and turns back to feeding.

At one point I see the pup pick up a grapefruit size piece of meat.

The yearling is wagging her tail.

I would estimate that she hits it about 25 times.

The pup walks off uphill and the yearling goes to the site. The pup comes back and takes over the site again without dispute from the gray. She continues to poke it with a paw and it reacts by lunging at her.

A black pup has come out in the gap.

I see 890 sniffing around in the gap. A gray pup is with him.

The black pup is following a scent trail to the south of the gap. It sits up and looks toward the den area.

The gray yearling is bedded by the sage den and the black pup is with her.

A gray pup is with the other black pup uphill and they are playing together.

At 2006 the gray yearling is at the feeding spot.

907 is now in the lawn. A black pup runs to her.

The wolves are howling there. I see the drab gray yearling and the black male yearling along with a lot of pups there. All of them go out of sight to the right.

A bit later I see adult wolves going north, above the horizontal forest. 890 is leading. Both black yearlings, 911, 907, 969, and the drab yearling are also in the group. It is 2029.

They continue north after going past the horizontal forest. I later lose them going north in the yellow grass meadow area at 2048.

I go east at 2052 and do not get any signals in Lamar.

I later hear that Melba saw 755 this morning and had the Wapiti white female and the gray female with three black Mollie’s by the sand pit operation near the bridge.

July 11:

It is 41 when I leave at 0449.

1014 was back in Pelican Valley last evening.

I do not get any signals in Lamar.

I see a small snowshoe hare at the side of the road west of Upper Baronett. It has some white on the hind legs.

It is still somewhat dark as I approach Slough at 0525. I do not get any signals.

Lizzie arrives a few minutes later and gets weak signals from 890.

There is a light coat of snow on Prospect and I hear that snow is on the Dunraven Pass

Road.

At 0551 I get good signals from 969 and weak ones from 890 and 911.

A black pup appears on the trail below the east end of the eastern trees. A black yearling is just east of there in the sage. The pup sits up and looks south.

What looks like the limping gray yearling goes up toward the eastern trees. A bit later she goes back downhill and sniffs around.

I see a black pup and a gray pup up at the lawn. I soon have three black pups there. The black pup near the eastern trees would be the fourth black.

969 goes north through the lawn. We later see her passing by the crescent rock and heading toward the eastern trees.

The gray female yearling is with the female black yearling and they are just above the dead tree meadow at 0643.

Twenty two horses and mules had come down the campground road earlier and passed us going south. We later heard that they had gotten lose from an outfitters camp well to the north. I called dispatch and Kevin Dillon, a black country ranger at Tower, said that he would come over.

The horses went toward the road entrance and I lost sight of them. Later we saw them going northwest through the rolling hills west of us. We lost sight of them, then saw they going west through aspen pass. Later they reappeared north of aspen pass and moved north, somewhat toward the general den area.

Two black pups are visible up by the diagonal forest at 0646.

The black and gray yearlings are looking toward the horses to the southwest. People tell me that the gray just howled while looking that way.

I do a check at 0739 and only get 994.

I see the drab gray yearling at the lawn at 0800. A black pup and a gray pup are there.

The limping gray yearling is still looking toward the horses.

The horses are now near the small diagonal forest and heading toward the den area.

The black yearling is also looking that way.

At 0726 the gray yearling is moving off to the right by the east end of the eastern trees. She is likely moving away from the horses. I see a black pup there.

The black yearling is standing and looking at the horses.

I soon have 907 going toward the crescent rock from the eastern trees. 890 is also there and going northeast, toward the diagonal forest. The limping gray yearling and 994 are on the top of the crescent rock and watching the horses. The black female is nearby and sitting up as she watches the horses.

890 starts to go back to the southwest.

The black female is in that area and she is casually going toward the horizontal forest. She does not seem too concerned about the horses.

The horses and mules are now in the upper part of the lion meadow, near the broken off stump. They go west through the meadow downhill from the sage den and veer back toward the small diagonal forest to the southwest. They soon are back below those trees.

The wolves are looking that way.

Kevin has been on the scene for some time monitoring the horses.

The limping gray has been watching the horses from the crescent rock. She now goes northeast.

The black female is going back to the eastern trees. 890 is going west through those trees.

The horses are now going back to the north, toward the den area.

At 0830 890 is watching them. Then he walks around by the bow log below the natal den.

I lose sight of him, then see him at the natal den. He goes back downhill.

A black pup is heading downhill to the sage den. A gray pup is at the den entrance. The gray goes up toward the black and both seem cautious about approaching each other.

That black pup goes to the sage den and stands there with another black pup. One goes into the den. The gray pup is sitting up above the den.

The horses have gone back south and are again by the small diagonal forest.

890 has had his head down in the tall grass by the bow log for some time. I see that there is a black pup there with him. That means at least three black pups and a gray pup are in the area.

890 goes toward the eastern trees and the pup follows. He passes behind the far east den of those trees with the pup still following.

No pups are visible at the sage den. They may be in the den.

At 0844 the limping gray is by the broken tree stump east of the dead tree meadow, where the horses and mules had been. She goes southwest and probably is on their scent trail. As she goes that way her head is high like she is looking for them. She is now southwest of the two dead trees and going toward the small diagonal forest. At times she goes at a slow run that way.

890, a black pup and a gray pup are going by the crescent rock toward the big diagonal forest.

The gray female is now below the burnt stump just north of the small diagonal forest. She had not been able to see the horses so far. But now she sees them on the south side of those trees. The gray turns around and runs back toward the den area. She stops and looks back at them.

I see a black pup and a gray pup heading toward the top of the big diagonal forest. 890 is bedded southwest of them. He gets up goes toward the gap and seems to be looking for the pups.

I see two gray adults and a black adult go through the lawn. Then 890 goes through.

The horses are going north by the burnt stump. They are heading toward the western trees in the den area.

The limping gray is now in the lawn and looking northeast.

We saw three horse riders come into the area below the big diagonal meadow. They continue on toward the upper part of the lion meadow and now must be seeing the horses to the west.

Kevin had to leave, and he gave the cell number of one of the riders to Lizzie and asked that she keep them informed about the location of the wolves. The horses are going toward the den area and we assume that the riders will be going to them. Lizzie calls the number, but no one answers. She leaves a message.

I call Tom Schwartz and ask that he come to Slough to help deal with the situation. I hear dispatch call him to say they just got a complaint call about the riders approaching the den area.

The horses are veering uphill by the western trees. They go behind those trees and soon are uphill from the ledge above the den area. I lose them going west through the pass.

A black pup came out of the sage den and went back in during that time.

The riders go through the den area by way of the dead tree meadow and pass between the natal den and the sage den as they head toward the area below the north end of the ledge. They ride uphill there and are soon above the ledge. Then they go out of sight on the route of the horses through the pass to the west.

The riders must have been with 100 to 200 feet of the sage den with the pup or pups likely inside. We do not see any other wolves in that area. The others are probably up at the big diagonal forest and I do not know if they were watching the horse herd and/or the riders passing through the den area.

I see a black pup to the right of the lawn.

I get weak signals from 890 and 994.

I go down the road and see the male gray yearling at the sage den at 1007. He goes in the den and comes out, then goes downhill to the south.

At 1028 we see the three riders leading the horses and mules down along the south side of the big diagonal forest. Kevin Dillon, Kevin Dooley and Tom Schwartz are here now. One of them gets a rider on the phone and is told that the wind must have prevented then from hearing the ringing phone. There were 23 messages on the phone about the situation. Tom asks them to stay out of the big diagonal forest after hearing from me that the wolves could be in there. He asks them to go down to the creek, then turn north. They comply.

At 1040 I get good signals from 890 and 994 and weaker ones from 969.

I go to the entrance area and take on last look at 1049. In the area north of the horizontal forest I see 890, 911 and what looks like the gray male yearling. 890 leads north and beds. The other two follow and also bed there. I might have seen a third gray adult.

I go east at 1056.

I do not get any signals at Footbridge at 1118.

I later hear that this was the Black Mountain Outfitters and they have a camp north of the park border.

It is 47 when I leave at 1728.

I do not get any signal in Lamar.

I get to Slough a 1808 and hear that earlier 890, a collared gray, an uncollared gray, a black pup and a gray pup were seen around an hour ago.

At 1815 I get a loud signal from 890. The other three signals are weak.

At 1819 I see 890 getting up, then bedding again on the slope just uphill from the horizontal forest.

People at the third lot show me a spot on the lower north side of the diagonal forest where they saw 907 digging at the base of a tree. That site would be just south of 890.

The people saw seven of the eight pups in that area around 1400 and they had a group howl.

I only get 890 and 969.

890 howls and looks south, toward that area. At 1932 he gets up and goes that way. 911 gets up between 890 and that tree and 890 gives him a subordinate greeting and rolls on the ground under him.

More wolves come in from the south toward the two males. I have the black female yearling and five grays there: 969, the three yearlings, and another gray that must be 907 or 994.

That would mean I have not seen the black male or 994.

Most of the wolves go to the site where 907 had dug and I see wolves digging there. They are wagging their tails.

I get all four signals at 2003.

I leave Slough at 2036.

I do not get any signals in Lamar.

I hear that two black Mollie’s wolves were seen today with the two Wapiti females. One of the blacks was collared and probably was 1014. The males and females reportedly were flirting. That included sniffing, jumping on backs and putting their heads on the backs of the others. They were in the Sour Creek RS and near the Wapiti Lake trail. 755 and the pups were not seen during the day, but he was spotted crossing the Chittenden bridge to the east at 2230.

July 12:

It is 37 when I leave at 0446.

I do not get any signals in Lamar. There is valley fog here.

I arrive at Slough at 0525 and only get a good signal from 890.

I see a black pup going southwest by the crescent rock at 0535. It goes to the sage den and looks south in an alert posture. Then it goes west to the log meadow and sniffs around.

A black bear is just north of the horizontal forest. It goes north, then veers east toward the creek.

890 and a black pup are on the south side of the diagonal forest at 0558. After walking around the pup goes into the lawn section of the diagonal forest. I see a black yearling there as well.

I go to the third lot and see 890 and the black yearling bedded in the lawn at 0604.

I only get 890 here.

A black pups and two gray pups are there. They play together and some of them harass 890.

890 walks out to the south and beds in the open.

I join Doug on the hill east of the road at 0800.

I see a video of the two Mollie’s black males interacting with the two Wapiti females.

At 1012 the black male yearling comes in from the south and goes by the western trees.

I see a gray pup carry a bone and going northeast from the crescent rock.

The black male goes up and over that rock formation. He reaches the lawn and a black pup chases him. It looks like he does a regurgitation to the pup.

The other black yearling that was with 890 in the lawn must have been the female.

I see at least three black pups and two gray pups in the lawn.

I only get 890 when I leave at 1047.

I do not get any signals in Lamar.

There are 11 cars in the horse trailer lot at Hitching Post and there is room for only one trailer. People are not paying any attention to the signs or sawhorses in the lot. Another seven cars are in the other smaller section by the outhouse. I count 23 cars in the Footbridge lot and the only open spot is the handicap section. I think most of the cars are connecting with fishermen and hikers.

It is 63 when I leave at 1731.

The rangers have put up roaps in the horse trailer lot and that should help manage the situation.

I do not get any signals in Lamar.

I do not get any Junction signals at Slough at 1813.

I look from several angles and do not see anything. A man in the third lot hear one wolf howling from the direction of the diagonal forest around 1730.

No one I talk to has seen wolves this evening.

I get weak signals from 890 at 1801 and 1938.

Lizzie tells me that she heard that at 1645 the two Wapiti females and the two black Mollie’s males were seen near the Sour Creek RS. They went west and tried to cross the road. The people went up on the hill west of Grizzly Overlook but could not spot them again. Lizzie had gotten 755 around 0800 toward the RS area. His signal dropped off at 0838. The area was fogged in. She did not get any Mollie’s signals in the morning and did not see any wolves. Since she did not get any Mollie’s signals we wonder if the collared black male is 1015 rather than 1014.

I do not get any signals at Slough when I leave at 1951.

I do not get any signals at Footbridge at 2008.

July 13:

It is 34 when I leave at 0446.

I do not get any Lamar signals on the way out.

I do a check at Slough at 0524 and get 969 and 994.

At 0535 I see a black pup running south through the lawn. Doug also saw a gray pup as well there.

I go down the road to try other angles.

I see a grizzly up above the diagonal forest. The black female yearling goes up toward it at 0553. The bear looks at her. The wolf goes closer as the grizzly moves of uphill. She soon turns around and goes back downhill. The black pauses and looks back at the bear. Doug had also seen a gray adult in that area.

The black is back on the south side of the diagonal forest and sits up there. She soon continues on and I lose her behind the ridge east of the eastern trees in the den area.

At 0601 I get 969 at the second lot.

I see the gray male yearling in the gap. He goes downhill and soon is in the lawn

I see pups with him in the lawn at 0602. I see five pups briefly, but the colors were had to see. Doug had three gray pups and two black pups. I see pups running around.

I have a gray adult downhill from the eastern tree that it has a GPS collar. Its tail is wagging.

The black yearling is on the grassy hill near the sage den.

At 0633 I see pups running around near the lower right side of the diagonal forest. Two gray yearlings are there, including the male. The black male yearling is also there. A GPS gray is there too. I later see that it is 907.

Yearlings are continually playing with the pups and often chase them around. Pups play with each other and wrestle and chase one another back and forth.

I eventually have all eight pups there.

The wolves at times are in a low area where it is hard to see them.

I go up Dave’s hill and look from there at 0717. This is a better angle.

The wolves move north on the back side of the horizontal forest. 907 is leading and she comes out north of those trees. A black yearling and a black pup are following her. Two other gray adults have bedded south of them.

I hear the wolves howling and see the lead ones going back to the south.

I see the pups howling.

The gray male yearling is leading back to the original area.

I wonder if they went back due to hearing other wolves howling.

Some of the pups are lagging behind but are following the scent trail of the lead adults. They stop and look south.

907 is now going back north to those three black pups. The fourth black pup was with her. The incoming pups pester her, and she does a regurgitation. I see pups walk off with tidbits in their mouths.

I see 994 and the drab female yearling there. I had not seen them before so perhaps they had howled, and the others came back to them.

The black male beds at 0737.

The limping gray female comes in from the south and joins them.

Adults are now playing with the pups. They are on the lower northern side of the diagonal forest.

The drab female rolls on her back with pups all over her. Later she goes over to what looks like the bedded 907 and gives her a submissive greeting.

So I have the three gray yearlings, 907, 994, and the black male yearling.

Most are out of sight at 0747. Then I see the drab gray play with a gray pup and nip at it. They go south and I lose them at 0751.

I see 907 at 0753 in the lawn. I hear howling. More wolves come into the lawn. They bed.

Doug saw the limping gray yearling go in and out of the sage den. I see her in that area. At 0816 she goes east from there. Then she heads up toward the lawn. She sniffs around the lawn. Apparently, the other wolves have left that site. Then I see a gray pup near her.

I do a check at 1000 and get three signals, all but 911.

I go east at 1011.

I get moderate signals from 926 in the Dorothy’s Knoll area at 1028. I do not get 965. 929 is better east of the Institute.

I also get her at the confluence, along with 965, at 1040. On direction she is good upriver and toward Norris.

I look, then head in.

Yesterday Emile thought that he had a black pup at the Sour Creek RS.

Lizzie called to say that the two Wapiti females and two black Mollies were seen west of the road near the gravel pit road north of the bridge in the early morning by Melba. They went out of sight heading north. Melba saw 755 by the bridge. Lizzie got 755 to the east near Otter Creek.

Melba called and filled me in on the day’s events in Hayden. They had 755 west of the road near the bridge around 0610. He tried to cross the road to the east but was blocked by cars. He did a lot of howling. The two Wapiti females and two Mollies blacks were at Cascade Meadow at 1150 and they went out of sight to the south. One of the blacks was collared. There may have been a third black in the group.

At 1256 755 was west of Otter Creek and trying to cross the road to the west but was blocked by traffic. He did a lot of howling. Ranger Rebecca stopped traffic and he crossed to the east at 1530. 755 looked very thin. People soon saw him with the four pups in the RS area, just across the river, to the east of the Alum Creek lots. There were two regular grays, a black, and a gray that was very dark on the back but had light legs. Lizzie later saw photos of those pups.

We got some vague unconfirmed reports that later the two Wapiti females joined 755 and the pups in the RS and that the two Mollie’s males were not with them.

Later 755 and two gray pups went south from the RS. They went into the trees in the sand box area of the RS. After that they were seen to the north and were going west. He did a lot of howling.

I checked the GPS site and saw that 1014 was east of Pelican Valley today. That seems to confirm that the one collared black in Hayden is 1015.

It is 63 when I leave at 1726.

I do not get any signals in Lamar.

I get 890, 911 and 969 at Slough at 1826.

At 1857 I hear group howling, then see two gray adults just south of the gap. Three black pups are near them. The black female yearling is there as well.

One of the gray adults is 969 and she howls.

I now have two gray pups.

I see the limping gray female yearling the gray male yearling and 911 there. 907 is also in that area.

A gray pup howls

Later the adults howl. 969 continues to howl after the others have stopped. 911 also does a lot of howling.

I go east at 2033.

July 14:

It is 36 when I leave at 0435. There is frost on the windshield.

1014 was in Pelican Valley last evening.

I do not get any signals in Lamar.

I see wolves going north, north of the horizontal forest from the first lot at 0526. I eventually see 890, 907, 911, the male gray yearling, a gray female yearling, the male black yearling, four gray pups and two black pups. 911 is leading.

We lose them behind the long ridge that leads to the yellow grass meadow. I only had them for a short time and may have missed seeing some of them.

Doug says he saw 969 and all four black pups in the group.

Ken calls at 0604 to say he has the three Lamar wolves running east, to the south of the Picnic Area. They have been looking back over their shoulders to the west. Ken has not seen other wolves that way. I go east.

I see a grizzly on Amethyst Bench at 0613.

At 0615 I stop at the confluence lot and see 926, 965 and Small T to the southwest, west of the river. Small T is chasing a coyote at top speed and is only a few lengths behind.

I hear several coyotes calling out in alarm.

I lose Small T then hear what might be coyote yips of pain.

The wolves chases coyotes back and forth in that area. There probably are coyote pups in the area.

Both black females have good coats and 965 looks much better. He still has a few spots on his face. His sides and back are now looking normal.

926 goes to 965 with a raised wagging tail.

When the wolves are not chasing the coyotes the coyotes follow them.

965 does two RLUs.

926 is limping a bit on a hind leg.

The wolves move off to the south and go to a small group of bison. They harass a small calf that is with its mother apart from the other nearby bison and 965 nips at the side of its neck a few times. Other adults come over and the wolves move off.

The wolves are in the Chalcedony RS. They go up on the high bank just west of the big cottonwood forest. I lose them in the thick sage there.

Ken said there were four adult coyotes involved with the wolves.

I go back to Slough. Doug says the wolves had gone to the lower right side of the yellow grass meadow, then came all the way back to the area on the lower right side of the diagonal forest. It is 0722. All eight pups and several adults came back from the north. 911 was the first adult to turn around and head back. He had 890, 907, 911, 969, the black male, the drab gray and the male yearling

At 0732 I see a black in that area.

I see the wolves coming out in that area. I have 911, a black pup, and a gray pup. I go down the campground road to get a better angle at 0737.

I do not see them down there.

At 0800 Doug sees a black pup in the lawn.

I only get weak signals from 890 at 0805. Later his signals is better.

I go west from Slough at 0853.

I hear that people saw the Canyon alpha pair and two gray adults. The alpha female looked like she was nursing.

I do not get any signals at the Nature Trail lot at Blacktail at 0939. I do a few scans of the area south of the road.

I go to Gardiner for a doctor’s appointment then head to Livingston for some X rays at 1057.

When I get back to Gardiner I talk to Melba and she says 755 was seen early this morning. Rebecca had him going south from the RS. Now they have the two black Mollie’s with the two Wapiti females, and they are heading toward the Nez Perce Picnic area. They lost them there at 1615. She thinks there is a carcass there. That call was at 1647.

I later hear that the four were first seen at 1430 at the point of trees in the RS.

At 1758 I only get weak signals from 890 at the first Slough lot.

I see a collared gray going toward the diagonal forest by the crescent rock at 1810.

I hear that the two black Mollie’s and the white female came back from the south at 1800 and are now visible from Grizzly Overlook. The female had blood on her face presumedly from a carcass. Both males had full bellies. The gray yearling was not with them. The three went out of sight heading west.

At 1857 I get weak signals from 890, 969, and 994.

At 1920 I see the black male and a black pup south of the diagonal forest. The male howls. He howls. I spot 907 south of the trees.

911 is bedded in the lawn.

I have four black pups and two gray pups in that area.

The black male has gone down to the lion meadow. 911 follows him. The black later beds and chews on a bison skull.

I have lost sight of 911.

I see 890 south of the diagonal forest at 1958.

907 is bedded on the grassy hill above the dead tree meadow. Four black pups are with or near her.

I get signals from 890, 969, and 994.

I go east at 2017.

I do not get any signals in Lamar.

I later hear that Emil saw a pup at Blacktail today.

July 15:

It is 43 when I leave at 0450.

I start getting 926 in Soda Butte Valley. She is loud at Trash Can, but even better at the Institute. I get 965 as I approach Dorothy’s. He is weak and 926 is also weak there. Both are best at Coyote and are probably to the north. I also get them as I approach Lamar Canyon East.

Doug tells me that some of the Junction wolves are near the south end of the den ridge. They have been howling. He also has been hearing howling from the diagonal forest.

I park at the first lot and walk out to our spot to the northeast. At 0556 I see the female black yearling, the limping gray yearling and 994 to the west, near the base of the south end of the den ridge.

I have seen wolves every day now since 12/16. That is seven months in a row.

Lizzie is only getting 969. I see her in the lion meadow playing with pups. There are three black pups and three gray pups with her. 969 does a play bow as a pup runs to her. It rolls on the ground under her. 969 runs back and forth among the pups. She pins a black pup. Some pups play together apart from 969. At times she goes from one pup to another and interacts with them one at a time.

The group moves to the meadow below the diagonal forest.

I hear that earlier another black pup was seen near the gap.

The group goes uphill on the lower northern side of the diagonal forest with 969 leading. They go out of sight at 0628.

I also hear that 911 was seen earlier.

I look back at the southern end of the ridge and see the black female going south. The gray female is going north. 994 is out in front of her.

I get a report of howling at 0646. 994 stops and looks north, like he is hearing the howls. I hear the howling.

The limping gray yearling is with 994 and she howls at 0651.

They go up toward the diagonal forest. Doug sees the female drink in the dead tree meadow. She ends up in the lawn. No others seem to be there.

994 is on the grassy hill near the two dead trees.

I hear that 755 and the gray yearling are being seen south of Grizzly Overlook.

No wolves are in sight at 0805.

I leave at 0827 and head to Hayden.

I hear that 755 followed the gray yearling to the Nez Perce area where there presumedly is a carcass. They were last seen from the lot with the three signs, then went out of sight to the south. Later 755 was seen in the Nez Perce area. That was around 0810.

Barb Moll saw the two black males and two female coming back from that area with bloody faces yesterday. People think the wolves got an injured elk calf that was seen down there.

Those black males and the white female have not been seen yet today according to Melba.

At 1026 I see 755 and the yearling across the river from the three signs lot. They are going north toward South Creek. After they move further north, I go to Grizzly Overlook and walk up the hill to the west. At 1037 we see them coming in from the south. They walk along the far bank of the river and do not go to the Sour Creek RS area. 755 at one point wades into the river and does a RLU. The yearling leads north just on the other side of the river.

We lose them continuing north in the Alum Creek area at 1106.

People at Grizzly Overlook tell us that the wolves went into the conifer forest on the east side of the river. That forest includes the den area at Otter Creek.

I go to Wapiti Trailhead. At 1128 I get faint signals from 755 there. It gets a bit better.

I go back to Grizzly Over and look from there.

Barb calls to say she just had 755 run in from the south. A collared and uncollared black chased him, but not too aggressively. The white female was behind the two black Mollie’s.

755 had blood on his face. He ran out of sight to the east and the blacks went that way. They were lost behind a hill. 755 stopped and look back, then went out of sight to the north. The other wolves did not continue after him and were not seen again. They probably went back to the south.

The chase was not at a fast pace.

Photos of the chase showed that both Wapiti females were with the two blacks.

I look at photos of the blacks and one has a regular looking collar that Dan later tells me would be 1015, not 1014. 1015's collar does not have the white plastic on the top while the one on 1014 does.

I get 755 at 1233 but no Mollie’s signals. I go north at 1301.

I get weak omni signals from the two Lamar wolves at the Exclosure Fence at 1424 and do not get them at Footbridge.

Barb hiked out on the trail from the lot and did not see any wolves.

It is 74 when I leave at 1749.

I get weak omni signals from 926 from Footbridge and in the den area. I get weak signals from 965 there and at the Picnic Area.

I get to Slough at 1829 and do not have any signals.

I later get weak signals from 969 and 994.

I see the black female yearling in the lower part of the lion meadow and later see all four black pups going up the rock outcrop on the lower south side of the diagonal forest.

I go east at 2026.

I get weak omni signals from both Lamar wolves at the Picnic Area at 2038.

I later hear that at 2015 the white female was seen south of Mud Volcano on the west side of the road. She howled for 30 minutes and there were answering howls from east of the river. She crossed the road and river and was seen with two blacks. They went north at 2100.

July 16:

It is 44 when I leave at 0442.

I get weak omni signals from 926 from east of Footbridge through the Exclosure Fence area.

When I arrive at Slough at 0525, I get a good signal from 994 and a weaker one from 969.

At 0546 I see a black adult and a black pup at the lawn. They go left and I soon have a black yearling coming out in the gap.

I soon lose the black. I do not see it or the pup again.

I go back to the car and at 0634 get 890, 969, and 994.

Carl calls to say he had a black adult, two black pups and a gray pup at Blacktail. They were going west, toward the Island of Trees. Then he lost them.

I go down the road to the fourth lot and get 969 toward the Yellow Grass Meadow.

While I do a talk at Bob’s Knob, I show the kids the grizzly family to the south. The cubs and mother chase a coyote.

At 0750 I hear that a collared black was seen from Grizzly Overlook.

I just get weak signals from 890 at 0835.

I leave Slough at 0839 and head to Blacktail. I get there at 0912 and do not get any signals. I do a few scans then hear that the group of three black Mollie’s and the two Wapiti females are visible from Grizzly Overlook. I leave at 0935.

I get to Grizzly at 1037 and see the three black Mollie’s. That includes an uncollared jet black, 1014, and 1015. The Wapiti white female and gray yearling are with them. I hear that the white female has done some scent marking.

I see the white female standing over a bedded black as she wags her tail. Then she interacts with 1014. After that she sniffs at the bedded black. That is the uncollared male.

I get a weak omni signal from 1014. He is three and 1015 is two.

755 is eight and the Canyon alphas should be eleven.

I hear that the gray yearling put her head over the back of 1014 and 1015. Then the white female came in and did that to 1014.

People tell me that none of the wolves have gone to the Sour Creek RS since the Mollie’s arrived. All the wolves have bypassed that area and gone north toward Otter Creek. On 7/16 755 and the gray yearling went through the open section of the RS area without stopping.

Deb tells me that on the 13^th^ the white female and two blacks came in from the south, passed through the area and went toward Otter Creek.

The uncollared black has a white spot at the tail scent mark. 1014 has a similar spot there.

One black seems to be pinning the other two blacks and he is probably 1014.

1014 is a bit more mottled than 1015. I see that 1014 looks thin. He sniffs under the white female and she jumps away. His collar looks light on top and I see the white plastic there.

The collar on 1015 is dark brown all the way around.

1014 sees to be snapping at flies.

The white female interacts with all three blacks. They all do a lot of tail wagging.

One collared black pins another black.

The male are just as friendly with the gray yearling as the white one.

One of the blacks has a slight limp. It may be 1015.

1014 puts his chin over the back of the white female. She playfully nips at her. Later she does more lunges and nips at him, but they seem playful.

The uncollared black does a RLU and GS.

The gray yearling does what looks like a FLU. One of the blacks marks the spot. The white female walks by without marking it. 1015 does a RLU there. 1014 sniffs the site and walks off.

The wolves are going south, parallel to the forest. The gray yearling is usually leading.

The uncollared black does a LFU.

1015 does a RLU and GS. The gray and uncollared black go by the spot. 1014 marks a nearby spot. The white female does a FLU at 1014's spot.

The gray continues to lead. The others veer off her route a bit. She sees that, goes to them and leads once more on their route south. It is 1410.

I go south to stay with them at 1430.

We see them off and on. At 1439 they come out of a gully further south and stop to look at a large herd of elk cows and calves. At first the elk do not see them. The white female is out in front of the other wolves.

Now some of the elk see the wolves. A number of cows and calves run south but the other elk stay in place. The wolves also stay put and watch the elk.

The white female looks back at the other wolves. Then she and the gray go forward with low heads at 1444. They stop and watch the elk.

The three black males are going south on a hill south of the elk. They stop and look down at them. The white female looks at the blacks and they look at her. She beds.

Soon the two females go forward. The lead black is also going south on the hill. The lead elk run south. The white female is in a stalk. Now she runs at the elk. I lose the elk members of the herd in a gully to the south. The white female is leading toward them and the others follow.

The elk bunch up and come back to monitor the wolves. The three blacks stop. The elk run toward them. One cow is collared. The three blacks back off. The gray is with them.

At 1450 all five are together. They socialize.

All the elk are stopped and staying in place. The wolves go toward them. We lose sight of them in a low area behind the elk. I see the heads of the elk moving in a way that indicates they are following the movement of the wolves to the south.

We lose the wolves after that.

I go down the road and see a cow elk run out of a gully. The white female and some of the blacks are in that gully and stay there. We wonder if the wolves got the cow’s calf.

I go to the three sign lot and at 1530 see two of the blacks up at the edge of the forest.

I go north at 1543 and do not get any signals from 755.

Deb later tells me that the white female came back to the north by herself with a full belly.

At 1745 I get 890 at Slough and at 1801 also get a weal signal from 969. Bill is here and he is not seeing any wolves.

Deb saw the white female going north through the gully at the Otter Creek RS. We wonder if the pups are there.

I go down the campground road and at 1812 get 890, 911, and 994.

I later go to Lamar Canyon West and get three signals, all but 911.

I do not get any signals in Lamar.

Later in the evening Deb saw the white female going back toward Otter Creek with a leg in her mouth. That implies that the pups are there.

I got another report from Larry and Linda that they had the white female and the three black males at 2143 near the new carcass.

I later hear that wolves were seen by Emile at Blacktail, to the southwest of the triangular forest: a black adult and two gray pups.

July 17:

It is 41 when I leave at 0448.

I do not get any signals in Lamar.

I start looking at Slough at 0532 and see the Junction wolves in the lion meadow just north of the group of aspens. I have the black female yearling, the gray male yearling, and 994. All eight pups are there as well.

The black female leads south to the aspens. The pups follow. The adults play with the pups and chase them.

I only get 994 at 0539.

994 and the gray male run south, and I then see the uncollared male chasing a pronghorn fawn to the north. Both are going all out. 994 and the black female join the chase. 994 stays the closest to the fawn as it runs north.

The fawn turns around and runs south and soon is back where the chase had started. 994 is still with it. They are now near the half dead willow in the flats. They run toward the creek ford.

I see the fawn running through the creek to the south. I lose both to the south in the rolling hills.

A bit later I see what may or may not be the same fawn running across the campground road just north of us. The uncollared gray pup is chasing it. He stops on the other side of the road, sees us, and turns around and goes back to the west. It is 0622.

The pups are going north in the lion meadow. The black yearling is with them.

At 0657 994 wades the creek at the ford and goes back to the north.

There is a big group howl at 0715.

The black yearling is leading the group up the lion meadow.

890 comes in from the south and is going up the lion meadow. The uncollared gray male runs to him and tries to get a regurgitation. 890 continues on and soon is going into the lawn. I do not see other wolves there.

The gray male is following 890's route as he carries a bone north.

Doug went to Blacktail and says he saw two black adults going from the den forest to the triangular forest.

People at Hayden think the wolves got two calves yesterday.

I get weak signals from 890 and 994 when I go at 0911.

I get signals from 969 in the upper Antelope Creek area at 0948.

I continue south and do not get any signals through Grizzly Overlook at 1052. No one has seen anything here. People think the wolves did get a calf yesterday in that gully where I last saw them and where the cow elk came out of.

I go to the three signs lot and turn around at 1115 without getting any signals.

I move north and get a weak omni signal from 1014 at Wapiti Trailhead and again at the Otter Creek Picnic Area.

I get a moderate signal from 969 and 911 at Antelope Creek at 1230 and they seems to come from where people in the lot tell me that they saw a carcass and two wolves yesterday from 1100 to 1245. We do not see anything there now.

I stop at Slough and heat that one of the black yearlings was seen at 1122. I only get signals from 890.

I do not get any signals in Lamar.

Carl called in the afternoon to say he had the three black Mollie’s wolves and the two Wapiti females at what probably was a new elk calf carcass at Alum Creek. He saw the gray yearling leave the site and go north toward the Otter Creek area. In the evening people saw the five adults from Grizzly Overlook. There was a lot of howling and the gray female played a lot with the three black males. No pups were seen.

Bill heard howling from Round Prairie while doing his evening talk at Pebble Creek.

July 18:

It is 36 when I leave at 0448.

I get weak signals from 926 in Round Prairie at 0505. I get her through the den area. She was best in Soda Butte Valley. I do not get 965.

I get all four signals at Slough at 0533.

At 0540 I see 911 to the north of the lower part of the diagonal forest. I see three other wolves there: a GPS collared gray, a black yearling, and an uncollared gray yearling. They howl at 0543. The wolves look south. 907 is the other GPS wolf. I see more adults and pups in that area. I eventually have 890, 907, 911, 969, 994, the female black yearling and one gray yearling. All eight pups are also there.

890 leads north and soon is going through the line of willows and aspens north of the horizontal forest. The others follow. I lose the lead wolves behind the long ridge on the way to the yellow grass meadow. The black yearling is behind the leaders and is playing with the pups. Two of the black pups run ahead on the route of the lead wolves. I lose the pups and yearling going north.

The lead wolves come out in the yellow grass meadow at 0607. 907 is out in front. She stops and looks back, then goes west. 890 comes into that area. More adults appear there. One gray does a leap and pounce in some tall grass. I see 969 to the upper left of the lead wolves. She beds.

I have 890 and three gray adults there.

The black male yearling approaches the diagonal forest area from the south. He follows a scent trail toward the gap, then stops, sits up and looks down at the crescent rock. It is 0612.

I look back to the north and see a collared gray leading north through the aspens. It stops and looks back, then slowly goes northeast. The other wolves come out and follow that route.

I lose 890 to the north in that area on the route past the meadow. Other adults turn west in that area, including 911. Some of them run northwest into a forested area. The lead wolves are out of sight thereby 0626. Then I see 890 going that way. More adults and pups go that way. I lose all of them in trees.

I look back at the male black yearling and see him sniffing around the dead tree meadow. He beds there and howls four or five times at 0635. Then he moves off to the southwest.

Lizzie is not getting any signals in Hayden.

I go to the third lot and get three signals, all but 911. I hear howling that seems to be coming from the diagonal forest at 0723. Then I have a group howl at 0733 from the direction of the yellow grass meadow.

I see 890 and a gray pup on an outcrop just west of that meadow. I soon have more wolves in that area and see that they are going south. The gray yearling is leading that way, back toward the diagonal forest. They are west of the usual route and area going through scattered trees. It is now 0744. It looks like the black female yearling is leading with 907 and 890 behind her. The pups follow those leaders. I also had another collared gray. I lose them behind the long ridge going south.

I hear that the three black Mollie’s males and both Wapiti females are in sight at Alum. I leave Slough at 0820.

I later hear from Doug that only the black female and 969 came all the way back with the pups to the den/diagonal forest area.

I see a run over snowshoe hare near the Gut Road.

I get to Alum Creek at 0920 and hear that the wolves just went into the trees to the northeast. That area is the southern end of the long forest east of the river that runs from the bridge to this area. To the south of that site is an island of trees. Emile says there is a carcass in the main forest and that grizzly dragged it in there from the open area south of the trees.

I get signals from 1014.

The gray yearling comes out around 1130, then goes back into the forest.

I go north then at 1200 hear that wolves are coming out of the trees. Around 1215 I see the white female, 1014, 1015, and the uncollared black male in that area. Most are bedded and they get up and then bed again. The white female looks full.

The three blacks go east, and she follows. It is 1229. She beds and they continue east.

The uncollared black does a LFU. The white female sits up, looks at the blacks going east, and follows.

The uncollared black wades in the river and drinks.

1015 has a white spot at the tail scent mark.

The uncollared black leaves the others and goes to an area west of the point of trees in the RS and seems to be sniffing or eating at a site for some time.

The two collared blacks are bedded. The female sniffs a spot and does a FLU there. Then she wades in the water and drinks where the black had done the same thing.

1014 is up and leading to the southeast. 1015 and the female follow.

I go to Grizzly Overlook at 1242.

I look from there at 1251 and see the uncollared black coming back to the others. The white female and gray yearling run to him. The two collared blacks are behind the two females. The uncollared black has a low head as he comes in and greets the females and males. All are wagging tails.

1015 walks off to the east. He has a slight limp. He beds and the uncollared black goes to him and gives him a submissive greeting.

All the wolves bed in that area and are spread out from each other.

Emile said that the five adults wolves came in from the north and went to the original carcass site, then followed the scent trail of the bear into the trees. They came back out and bedded. There was howling from the trees to the east and all five wolves howled back. The white female did the most howling. People thought that it was 755 howling to the east, but Lizzie did not get his signal. The three black males got up and went that way. The lead black was collared, and he went east into those trees, near one of the sand pits. The other two males had turned back halfway there and went back to the two females, who had only gone a few hundred yards before stopping and bedding. The lead collared black came back and joined the others and there was a lot of play and interaction between the two collared males and the two females.

1014's points have not been registering since 7/14, but they did register this morning and in the early afternoon. There were points in the woods where we think they have the carcass and out in the open where I saw them bed in the early afternoon. I later checked previous points and saw that from 0030 to 0630 there were a number of points just north of Otter Creek in the trees. That could be where the pups are. He then went south and was the possible carcass site by 0830.

I head back to the north. I do not try for signals when I went by the Slough area and did not get any signals in Lamar.

July 19:

It is 41 when I leave at 0449.

I get a good signal from 926 at Picnic Area and she seems best to the north. I do not get 965. I continue on at 0530.

I do not get any signals at Slough at 0545.

Doug had one wolf that bedded on the sage den, then went up to the lawn.

I go to the third lot and at 0600 see two black pups south of the gap in the diagonal forest. Doug is seeing three blacks and a gray there from his position to the south.

I now see more wolves: two gray pups.

I only get 994. They run downhill. All the pups go out of sight behind the ridge eastern trees.

I see the male gray yearling downhill from the eastern trees. A black pup is with him. Both are looking downhill as they sit up. The black pup runs downhill. The gray male beds. I see a gray pup below him. I see a second black pup.

The yearling goes to the sage den, walks over it and continues down into the gully. A black pup runs there as well.

The drab gray yearling is up by the eastern trees.

At 0612 I get weak signals from 969 and 994.

A black pup is at the sage den and is sniffing around. It goes down into the gully.

Three cow elk are coming up the lion meadow. They move up to the dead tree meadow. A gray pup is sitting up at the eastern trees and watching the three cows.

What a gray yearling, probably drab, is going northeast by the crescent rock at 0637.

A black pup is going toward the crescent rock from the eastern trees. Two black pups come out of the gully and the gray male. I see a gray pup in that area. The lead black pup is soon on the crescent rock.

The three elk are in the lawn and show no evidence that they are concerned about the likely presence of a lot of fresh wolf scent.

At 0651 the lead black pup is going toward the diagonal forest. When it gets close to the gap the pup looks at the elk in the lawn. The pup is in an alert posture. It goes toward the elk, stops to look at them again, then turns around and walks away.

I only get 994 when I leave Slough at 0721 to go to Hayden.

I later hear that seven pups were seen at Slough after I left.

When I arrive in Hayden, I hear that two black Mollie’s were seen earlier to the east of the Alum Creek lot, just as the fog was lifting.

I do not get any signals through the Wapiti Trailhead lot at 0811.

I go to Grizzly Overlook and do not see anything.

Quinn is doing a flight and I hear that he saw two gray pups at Blacktail. Carl had seen two black pups and a gray pup there on the 16^th^.

I go further south and do not get any signals through the three signs lot at 0957.

I get back to Grizzly at 1017 and still do not get anything.

I do another check at the Wapiti lot, the head north at 1131.

I do not get any signals at Antelope Creek at 1158 or at Slough at 1235.

I get moderate signals from 965 at Trash Can and weaker signals in the den area.

I do not go out in the evening.

I checked the GPS points for 1014 in the afternoon and saw that at 0630 he was east of the Fishing Bridge Junction. At 0800 he was going north from there. From 1000 through 1530 there were a cluster of points west of the road near Elk Antler Creek. The group probably had a kill there. I also printed up his points for the last 30 days and had a lot in the open area west of the Sour Creek RS.

I later talked to Quinn about his flight today. He saw two gray pups, 964, and a black adult at the Prospect RS. Carl had seen two black pups on the 16^th^. Yesterday 996's points were in Jardine. He got the Eight Mile signals in Gardiner’s Hole, but did not see them due to trees. Quinn only got 994 in the den area. He saw 890, 907 and a black adult coming downhill in the Buffalo Creek area. They were heading toward the den.

He only got 779 out of the Mollie’s and did not see her. Her signal was south of the mush pots den area by Pelican Creek. Quinn did not get 1014.

He did get 755 two kilometers southeast of Wrangler Lake, near Cottongrass Creek.

Lizzie got a report that at 2030 755 crossed the road from the west at Alum Creek.

July 20:

It is 45 when I leave at 0448.

The den closure and No Stopping signals are down in the Lamar den area.

I do not get any signals there at 0518.

I get 890 and 969 at Slough at 0531.

Doug says it got up to 101 in Gardiner the last two days.

At 0559 I see Junction adults and pups coming out of the gap and going south. I eventually have 907, the three gray yearlings, the female black yearling, four black pups, and three gray pups in the group.

They interact a lot and they move south. 907 tends to lead.

The wolves go down to the lion meadow. Some of the adults bed and the pups continue south as they explore. The adults get up and follow. As the pups get distracted by various things 907 goes out in lead again.

She stops, sits up and stares to the south. I wonder if she is seeing or hearing other wolves.

The pups howl at 0616. I see that 907 is also howling. The pups go to her and also stare to the south.

A black pup leads south, and I see them approaching a bedded gray. That gray gets up and gives a holding bite to the back of the neck of a black pup. This is 994 and he leads south.

The wolves are passing by the group of aspens in the lion meadow. A black pup is leading south.

I call Lizzie and she says she has not seen any wolves in Hayden.

There is a lot of playing involving the yearlings and the pups. The yearlings chase and wrestle with the pups.

A big bison bull is coming up the meadow toward the wolves. All the wolves watch him approach. One black pup hesitates, then moves off. Other pups move closer to the bull to get a better look. The bull chases 994. Then he stop and looks at the wolves. The bull chases 994 again. After that he moves off.

The yearlings resume playing with the pups.

The wolves howl at 0646. The pups come together for a rally and howl apart from the adults.

907 is staring to the south again.

The black female is rolling on the ground under some pups.

I now see that 969 has joined the group. The pups are chasing her around trying to get a regurgitation.

A gray yearling chases a gray pup and tries to nip at its rear end.

A number of the adults are standing over a gray pup who is rolling on the ground and seem to be poking at it.

994 chases the gray male yearling.

969 leads south and the other adults and pups follow. They go by the half dead tree. That is the furthest the pups have been to the south.

The wolves continue south, and I see them off and on.

I see 994 to the lower left of the Simpson tree in the flats. At 0722 one of the female yearlings beds there.

I lose 994 in the far southern end of the flats as he continues south.

I see a black pup at the bend in the creek west of the ford area. It sits up and looks south. Then I see two more black pups, two gray pups, the black yearling, and 907 there.

The pups seem to be looking down at the creek in that area.

907 beds and watches the pups.

We see pups go down to the creek in that area. Doug sees 969 go down into that section of the creek. This should be the first known time the pups have been at the creek.

907 gets up and looks that way. She can probably see the pups at the water.

A black pup comes up out of that area. 907 goes down into the site.

They all come out. I see the seven pups along with 907 and at least two other gray adults and the black yearling.

The wolves go west and approach the willows at the bottom of the aspen drainage. Then we lose all them going north up into the aspen drainage at 0816

Doug goes to Little America to look up that drainage.

Bill sees the wolves coming out of aspen pass and going north. At 0833 I see the wolves going north from the pass. They are on the ridge about the two trees.

907, 969, the black yearling, the drab gray female and the gray male yearling are with the seven pups. The gray male is leading north.

I do a check at 0841 and get 890 and 969, but not 994.

The black yearling leads toward the sage den area. She and a gray yearling go to the dead tree meadow and sniff around.

Doug did see the wolves going up the aspen drainage from Little America.

The pups reach that meadow. 907 goes up to the eastern trees as does 969.

A black pup goes to the spring and seems to drink briefly.

Most of the wolves are now heading up to the gap. The male gray leads through the lawn and others follow. We lose them there.

I go to the fourth lot and only get 890 and 969. No wolves are in sight.

I call Lizzie and she still does not have anything in Hayden.

I get the same signals when I leave at 1022.

I do not get any signals in Lamar at 1040.

I checked on the estimated age of 712 and his white female and our list says they are 11. That would be about in human years.

Deb had the three Mollie’s blacks and two Wapiti females appear in the Alum Creek area after Lizzie had left. That was in later morning. The white female chased elk in the river area and the other four wolves did not help her. I later heard that all four pups were seen with the adults in the morning.

Melba called around 2030 to say Rebecca has been seeing 755 in the NPS employee housing area at Canyon.

Larry and Linda had the five adults and four pups by the western trees from Grizzly in the evening.

July 21:

It is 42 when I leave at 0445.

I do not get any signals in Lamar.

I only get 994 at Slough at 0529.

We do not see anything. I do not get any signals at 0608.

I leave Slough and head to Hayden. I got a message that wolves are out there.

I get good signals from 755 just south of Otter Creek Picnic Area.

I get to Grizzly Overlook at 0656 and see the wolves to the north, east of the western trees. I have the white female, the gray yearling and a bedded gray pup

Two gray pups and a black pup were seen earlier. One of the gray pups had a dark back and it went east toward the RS.

I see that gray pup coming back and it does have a dark back.

I hear that 1014 and the uncollared black went into the western trees.

I have a black adult and he goes west.

The gray yearling goes into the western trees.

Laurie saw the black pup go into the western trees.

I now have a black adult and the gray yearling with a gray pup and both adults are wagging their tails. The black is 1015.

I hear that 1014 and the uncollared black have not been seen yet, as well as the third gray pup.

I see the black pup east of the trees.

I now have 1014 going east. People lose him by the cut bank at the point of trees in the RS. The black pup goes that way at 0746.

Doug calls to say he heard howling from the trees to the upper right of the horizontal forest. That would be west on the route to the yellow grass meadow. He thought the howling was from pups. Doug saw a gray yearling north of the horizontal forest in the line of low aspens. It went north toward the yellow grass meadow. Later he saw the male black yearling near the western bend in the creek. He went to the area east of the diagonal forest, then traveled up the north side of that forest and went out of sight. Doug also had a gray pup by the leading aspen north of the diagonal forest after the black adult disappeared in that area.

At 0750 I see the uncollared black.

I hear him howling at 0751. Others howl back.

The uncollared black is going west.

1014 comes back from the east. He stops near the western trees and looks around.

At 0847 we see 755 way off to the northeast. He goes out of sight into trees in that area and we lose him.

Then we see the gray yearling going east toward the RS.

I do not get any signals at 0910.

I go north at 0934 and do not get any signals as I go north.

I get back to Slough at 1041 and do not get any signals and do not see anything. I go to the fifth lot and get weak signals from 911.

I go west from Slough at 1127 and head to Gardiner for an appointment.

I go back through the North Entrance at 1326

I get weak signals from 911 and 994 at Slough at 1430.

965 is weak at the confluence at 1507. I do not get 926. I continue to get 965 in Soda Butte Valley.

It is 86 when I leave at 1759.

I get weak signals from 965 east of the den area at 1827, then lose him.

I get weak signals from 911 and 994 at Slough at 1844.

At 1934 wolves come out of the gap area and go south. 911 is leading. 994, a black yearling, four gray pups and three black pups are also in the group.

The wolves go down through the lion meadow and continue south to the far bends in the creek to the west of us. 911 is still leading.

I see three of the pups going south along the far creek bank then lose them.

I spot the adults and other pups further to the south and west of the creek at 2001.

At 2007 that group goes into the gully that leads to the southern route tree. I see some of the pups by that tree a few minutes later. I do not see any of them after that.

I go at 1016.

I hear that the white Wapiti female, the gray yearling, the three black males and at least three pups were seen at Alum Creek this evening. One of the black males played with a gray pup.

I get 965 in the confluence and 21's crossing area at 2029, then lose him.

July 22:

It is 48 when I leave at 0441.

I start getting weak signals from 926 east of the den area. She is steady through Trash Can. I get moderate signals at the Institute.

I get her and 890 at Coyote at 0523. I continue to get her part way through Lamar Canyon. She is probably up high north of the road in Lamar.

I get a good signal from 890 at Slough at 0530. I join Doug on the hill and see pups on the north side of the diagonal forest at 0548.

This is the 100^th^ day in a row that we have seen the Junction wolves at Slough, starting on 4/14.

I eventually see all eight pups there. 890 is with them and the pups follow him to the north, toward the outcrop hill. At times he looks back at them. The pups stall out. 890 beds. A black pup goes to him and greets him.

Other pups go back to the south.

890 gets up and goes back to the south. He looks toward the pups that way. 890 beds again. I wonder if he went north to get away from the pups.

I see that the gray pups now have dark stripes on their backs.

I get 994 along with 890.

At 0720 I see 890 going north from the horizontal forest, past the bend of the aspen line.

I hear that 994 has come in.

From the fifth lot I see pups running to an incoming gray yearling from the north. This is the drab female. She does two regurgitations to the pups. Some pups remain at the feeding site and others follow her and try for another feeding.

I see 994 in the background. He goes to the pups and does a regurgitation. I see a gray pup carry off a good sized tidbit.

I hear from people at Hayden that a grizzly has gone into the western trees where we think the wolves have a carcass. The three black males and the gray yearling are in sight. It is 0736.

I leave Slough at 0747 and head to Hayden.

I hear that the five adult wolves and four pups are now visible there.

I get a good signal from 755 at Otter Creek and in the area just south of there.

I get to Grizzly Overlook at 0844 and see 1015 with two pups. I now have two gray pups and a black pup. Another black adult is bedded east of there. I also see the white female. 1014 is going toward her. 1015 is limping.

The pups play together vigorous.

They jump up to see over some sage the incoming 1014. They run to him and reach up to greet him in the muzzle.

One of the gray pups is much larger that the other gray pup. The larger one has a dark back.

The three pups resume vigorous playing.

I hear that all four pups were seen earlier

I see the uncollared black male by the point of trees east of there. The gray yearling is south of him and going his way.

1014 goes to them and all three are at the point of trees at 0907. The two males romp with her. She jumps on the back of 1014. All three jump up on each other.

I see the pups playing at the base of the cut bank near those adults. I have two gray pups and the black pup.

The three black males and gray yearling go south into the trees in that area and the pups go a similar direction. All are now out of sight in the trees of the Sour Creek RS.

At 0956 755 and 1014 are weak.

I hear that the uncollared black did a lot of playing with the pups. 1015 also played with them, but 1014 did less playing.

The two signals are weak at 1114.

I realize that 755's situation is somewhat like what happened to 890 after 970 died and he had to accept a subordinate position to 911 to rejoin the pack.

I only get 969 at Slough.

I do not get any signals in Lamar.

I later hear that 755 was seen at Hayden in the evening, near the Sour Creek RS. He looked to the west, toward the other wolves, then he left. 755 was visible for about 30 minutes.

I later hear that he was within 100 yards of a pup but that they did not see each other. The black males and two females apparently did not spot 755. Another report from Fred was that a gray pup was out in the new RS. 755 moved west and was near that pup, but they did not see each other. He went out of sight into the western trees at 1845.The gray yearling was out in the RS as well. Laurie later gets a report that the three black males did chase 755, but not in an overly aggressive manner.

July 23:

It is 42 when I leave at 0444.

I get good signals from 926 on the east side of the den and she seems to be to the north. She is better on omni than on direction.

I go to Slough and at 0543 see a black pup on the north side of the diagonal forest from the first lot. Doug had other pups and a black adult earlier.

I go to the fifth lot and see 969 and a black pup. The pup greets her, then runs off downhill. A gray pup runs downhill.

Doug is seeing more behind the horizontal forest. He has four black pups, two to three gray pups and two gray adults. The other wolves did not seem to see him.

I see a group of pups playing behind an aspen on the north end of the horizontal forest. 969, the drag gray yearling and the black female yearling are also there.

I hear that the three black males and the gray yearling are visible at Hayden, near the western trees.

I now have all eight pups there.

I now have 907, 994, the gray male yearling, and the black male yearling in that area.

The yearlings play with the pups and each other. The pups play with each other and with the yearlings.

The wolves howl at 0614.

The drab female runs back and forth as she plays with the gray male.

969 looks south as she howls. There is a lot of howling.

The black female rolls on the ground under 907.

The howling has continued for some time and 969 is still looking south.

A pup chases the female black yearling and grabs her tail as she trots off with a small stick in her mouth.

The gray male tries to bite the tail of a gray pup moving away from him.

I only get 969 and 994.

Most of the wolves are out of sight when I go north from the fifth lot at 0628.

I go up to the hill and see some of the wolves in that area. At 0718 some of the adults are going north. The pups are still playing.

I hear that the five adults and four pups are visible at Hayden.

I see 907, the drab gray and the black male up in the yellow grass meadow at 0825 and soon lose them going north.

I leave Slough at 0840.

I get good signals from 755 to the south of Otter Creek at 0930.

I get to Grizzly Overlook at 0936 and see two black adults and both females. The gray yearling is going west. I also see a black pup and a gray pup.

The gray pup rolls on the ground under the gray yearling. The black pup joins them.

A collared black goes to them and sniffs the pups. The yearling gets up and wags her tail. The black pup rolls on the ground under the collared black. He sniffs the pup. The pup later reaches up and sniffs the muzzle of the black adult.

I now have all three black adults there, east of the western trees.

1014 is limping.

The uncollared black goes to the bedded white female and she thumps her tail on the ground.

At 1010 the white female walks off to the west and does a SU. The three blacks follow her.

I go to Alum Creek at 1023. From there at 1025 the white female and the uncollared black are on the island with the trees. The two collared blacks are south of them and watching the pair.

The white female wades the water to the north and goes up the hill into the forest. A collared black and the uncollared black follow her. The third black also follows.

At 1110 the gray female follows the scent trail of the others through that area and also crosses the water to the north. We lose all of them in the trees.

I get 755 and 1014 at 1125.

I see the gray yearling again at 1128.

Around 1150 we see what we think is the gray yearling well to the east. She is looking west, toward where we had seen the other wolves. Then we realize that this is 755. He runs off to the east, stops to look back, then runs out of sight. Laurie later tells me that she saw one of the collared black males running halfheartedly in 755's direction.

A bit later we see one of the collared blacks going north behind the island of trees.

I do not get any signals at 1253.

We hear that people at Grizzly Overlook did see two black adults in the open when 755 was visible. The observers lost sight of the blacks and did not see any chasing of 755. They thought that 755 saw the two blacks and run off due to that sighting.

I do not get any signals at 1322.

I go north at 1353.

I later got a report from Fred that around 1500 he saw 755 by the point of trees at the old RS. He went west in a meandering route toward the western trees and cautiously looked around a lot. His belly was full. Fred lost him going into the western trees.

I get another report that at 2200 the three black males and two females were seen going south from Grizzly Overlook. They chased some elk for about a mile. Two pups had also been seen in the RS.

At 1505 I get 969 and 994 at Slough.

I do not get any signals in Lamar.

I later get a report of howling in the Antelope Creek area.

July 24:

It is 41 when I leave at 0450.

I do not get any signals in Lamar.

I get to Slough at 0537 and see one black near the lower right side of the diagonal forest.

I do not get any Junction signals.

I go out on the hill and see more wolves.

Doug tells me that a guy known as Panzer took photos of what he thought was the Cougar Creek Pack outside the park near West Yellowstone. There were five blacks in including a collared black with a white face. There were no grays.

I eventually see all eight pups. I see a black pup do some pounces for insects or rodents.

The black female yearling, the drab female yearling, and the gray male yearling are with the pups. I also see 907 there. She is still limping.

The yearlings play a lot with each other and with the pups. 907 does not interact with the pups much.

At one point the pups look south and howl. The adults join in.

Lizzie is at Grizzly Overlook and she is getting 755 toward the point of trees. It is foggy there.

I do not get any signals at 0634.

I go to the fifth lot and see 911 coming downhill from the west to the other wolves. He greets the gray male, then goes downhill to the gully where the pups should be. That was around 0645. Doug sees him do a regurgitation to them.

I do not get any signals at 0700. 911's signal is probably blocked with him being in that gully.

I look from the entrance lot and see a yearling playing with the pups.

I head to Hayden at 0708.

I talk to Lizzie again at 0801 and she says it has cleared. She has lost 755's signal but has seen the gray yearling and a black pup.

I get a good signal from 755 south of the Otter Creek Picnic Area at 0810.

I join Lizzie at Grizzly Overlook and just miss seeing a gray pup.

I go back to Alum Creek and see two gray pups near the island of trees. The first pup goes north into the western forest and the other pup goes east and I lose it.

People at Grizzly tell me that they saw 755 come out of the east side of that forest and go back in. I return to Grizzly.

A gray pup comes out and seem to be hunting in the meadow. It does pounces for insects and rodents. The pup continues far to the east.

I get a good signal from 755 at 0919.

Lizzie tells me that she saw 755 come out of the eastern point of the western trees. He sniffed around, then went back into those trees. Two gray pups had been seen in that area.

At 1015 we see 755 out to the east of the western trees.

The other two gray pups and the black pup are in the meadow near him. He beds on a low rise north of them and watches the pups. Soon he gets up and goes down to them. He sniffs around them and sniffs at the pups, then he rolls on the ground next to them and seems to be picking up a scent there.

I do not get 1014's signal.

There was a reported howl to the southeast earlier.

755 walks off from the pups and looks west intently.

The original gray pup that went east is coming back.

I hear that Eight Mile has 11 pups: 8 grays and 3 blacks. There are 8 adults.

The pups are running around and 755 gets up to watch them.

I soon lose him in the western trees.

Two of the pups are still out hunting.

I get 755 at 1205.

I go south at 1219 and do not get 1014 through the area about a mile south of Mud Volcano at 1228.

Deb calls at 1233 to say 755 crossed the road to the west, north of Alum, and was last seen going north. I go to Alum and do not see him or get his signal. He must have swam the river prior to crossing the road.

At 1257 I do get him at Alum. I go north and lose the signal just south of the Otter Creek Picnic Area.

I continue north.

The Wapiti pups may be a week or two older than the Junction pups and seem to be more independent and more into hunting for small prey on their own.

At 1422 Deb calls to say the three black males and two Wapiti females came in from the south. The blacks did a lot of sniffing around and must have been getting the scent of 755. The wolves also did a lot of howling.

I wonder how 755 knew that they were coming back a few hours before they arrived? I do not get any signals at Slough or in Lamar.

I later hear from Fred that he saw the three blacks come in at 1405. In the evening he saw the gray yearling come in from the north. Later the white female was seen going into the western trees with a pup.

I hear that a wolf was seen trying to cross the road in Soda Butte Valley this morning around 1045.

July 25:

It is 41 when I leave at 0451.

I do not get any signals in Lamar.

When I arrive at Slough at 0534, I get signals from 969 and 994 from near the first lot

I see a few blacks in the lower right side of the diagonal forest. I try looking from other lots, but the wolves are often in a low gully behind the south end of the horizontal forest and are not easily seen from those angles.

I do hear howling at 0546.

I return to the first lot area and see them again in that area at 0552. I eventually see all eight pups, 969, 994, the male gray yearling, the limping gray female, and the black female. The pups and yearlings do a lot of playing. 969 is much less involved in interacting with the pups than the yearlings.

At one point 969 gets up on a rock and looks around.

I go up on Dave’s Hill and look from there at 0624. I heard howling on my way up at 0619.

Emile saw two gray pups and a black pup at Blacktail and 763 was with them. They were behind the triangle of trees.

At 0717 two adults howl and look east.

I get back to the car at 0749.

I call Deb and she says there has been a lot of howling from the western trees, but that no wolves have been seen yet.

I can see some of the wolves from the entrance lot.

I leave Slough at 0802.

At 0837 I hear that one gray pup was seen from Grizzly.

I get a good signal from 755 in the area south of the Otter Creek Picnic Area.

I do not get any signals at Alum Creek at 0855.

I see one gray pup from Grizzly at 0907. It soon goes out of sight heading for the western trees.

I get weak signals from 1014 at 1012.

We see the gray yearling coming in from the Sour Creek RS area at 1040 and lose her heading for the western trees.

I go north at 1058 and do not get any signals through Canyon Junction.

I do not get any signals at Slough at 1211 or at Footbridge at 1235.

The bison have started their rut with the bulls grunting and following cows.

I hear that in Hayden the five adults and the four pups were seen in the evening. 755 was not seen.

July 26:

It is 49 when I leave at 0455.

I get a loud signal from 926 in Round Prairie at 0513. It is dark. I continue on to the west at 0519.

I look from the Slough entrance area and at 0542 see wolves in the usual area on the lower right side of the diagonal forest. I have three black pups, a gray adult and a black yearling. The two adults are playing. I see a second gray adult.

I go up on Dave’s Hill and at 0555 have a better view of that low area. I eventually have 907, the drab gray yearling, the black male yearling, three black pups and three gray pups.

The yearlings play with the pups and with other vigorously. The pups play together and with the yearlings. 907 was somewhat involved with play with the pups.

At times 907 looks uphill and to the south.

The gray pups have dark backs now.

The wolves howl at 0615 but are out of sight.

I call Deb and she has not seen anything yet. There is some fog.

I continue to see the pups and adults. I go downhill at 0635.

I do not get any signals at the car. I pull out at 0638.

Russ says he saw two collared grays here.

I start to get 755 at Canyon Junction at 0741. He is best south of the Otter Creek Picnic Area. He is weaker at Alum Creek.

I arrive at Grizzly Overlook at 0752 and see a black pup and a gray pup east of the western trees. The black does a short run like it is chasing something in the grass. I lose the pups heading toward the western trees.

At 0805 I see the gray yearling and the black pup at the grassy indentation in the western forest.

I do not get any signals at 0816.

We go up on the hill west of the lot.

A man tells me that yesterday at 1030 he saw a black adult and what looked like a black or a gray pup west of the road near the bison dangerous sign. It might have been the gray yearling.

We see the three black males, the gray yearling, and the white female to the northeast of the point of trees. The white female is well north of the others. Two gray pups are near the lead adults.

The white female going north at 0917.

The most northerly black is staring north, toward the white female, and howls.

I lose the white female going north.

Two black adults are now howling.

The gray yearling and two gray pups are sniffing around near the point of trees up on the bank. The uncollared black is near them and he howls. Both collared blacks are a bit north of them. They are now going south toward the others.

We see what at first looks like the white female again to the north, then realize that it is 755. He is well north of the others and going toward the western forest.

We now wonder if the white female saw 755 and was going toward him when we lost her. I also wonder if the black males were seeing 755 to the north.

755 is doing a lot of sniffing around as he goes west. His coat looks dusty gray. There is a horizontal line on his upper sides and that line goes diagonally down his front shoulders. He stops to look back at the other wolves to the east.

We lose 755 going in behind the eastern point of the western trees at 0930.

The three blacks are staring north and howling. They have started to go west but now are stopped.

A gray pup is going west toward the forest.

The gray yearling is west of the three blacks and I soon lose her running into the western forest where she should meet up with 755.

I see the white female again to the north of the point of trees, near where I had lost her. I still think she had seen 755 in that area and had gone to him or toward him.

Two of the blacks are looking at the white female.

The gray pup is going toward the three blacks.

The uncollared black is leading west.

The white female is following the route the other wolves took toward the point of trees and not toward where the three blacks currently are, west of there.

At 0938 I see that the blacks do not have raised tails as they head toward the western forest. They are sniffing at 755's scent trail and do not seem to be in an aggressive mood.

As the blacks continue west the limping collared one has a slightly raised tail. The three blacks follow 755's route into the western forest.

I see the white female west of the point of trees. She goes toward the western forest on a low rise and I see a gray pup in the grassy flats just south of her. It is also going west.

I realize that there should be a lot of scent trails made by 755 in the western trees and that the black males would need time to find the most recent trail.

I look at the indentation in that forest and see the three black males, the gray yearling, a black pup and a gray pup. They greet. It is 0944.

The white female continues west on the route of the three blacks and at time sniffs at their trail.

We see 755 northeast of her, going east, on a parallel route back toward where we first saw the other wolves. I now wonder if they have a carcass to the east and now 755 is going there to feed.

I see the other gray pup just east of the western trees.

755 looks back several times as he heads east. It is 0946.

The third gray is still moving toward the western trees and is east of the white female.

The white female stops and looks toward the western trees. She did not see 755 going past her to the east.

755 is stopped and looking back. We are impressed at how he gave the three blacks the slip.

The white female is stopped and howling. Both gray pups run to her and pester her for a feeding. She runs off and soon lowers her head and does a regurgitation.

Then she goes west, and we lose her and the two gray pups running to the western trees on the route of the others. They are out of sight at 0951.

We see 755 to the east. He is looking west, and his tail is a bit tucked. Then he runs out of sight past the point of trees at 0955.

A short time later the three blacks and the white female come out of the western trees and go east on a different route than the one they took coming in from the east. We wonder if they are on the route just taken by 755 to the east.

The uncollared black is leading in a straight line toward the point of trees at a fast pace. The two collared blacks speed up and pass the white female. The uncollared one at times has his nose down to get the scent.

As they approach where we lost 755 the white female looks back over her shoulder to the west.

The wolves go out of sight where we lost 755 at 1002.

We see two gray pup going east from the western trees.

Carl is at Alum and he calls to say 755 is going north from the point of trees. It is 1008. We do not see him. Carl saw the collar.

A few minutes later we see what looks like the white female going east into the big forest north of the point of trees. We think she is following the route of 755.

The two gray pups are running back to the western trees. They stop and sniff at a spot at 1028. Both of them have dark backs.

We see the white female and three black males coming back from the east.

The two gray pups are following a scent trail to the western forest.

The white female and three blacks go back into the western forest at 1048.

A gray pup is near there and taking a different route that way.

The uncollared black comes out of the trees and sniffs around where 755 had been with a raised tail. It is 1054. He runs back into the trees at 1056 with a raised tail.

I get a glimpse of two gray pups in the indentation area .

We do not see anything after that. I go downhill to the lot at 1124.

We hear that Deb and others saw 755 swim the river north of Otter Creek and cross the road to the west. It is 1141.

I get his signal as I go through there at 1150.

I do not get any signals at Slough at 1251.

926 is weak at Round Prairie at 1335.

I hear that in the evening three Wapiti pups came out in the meadow. The five adults went south at 1900.

July 27:

It is 42 when I leave at 0456.

I get loud signals from 926 as I approach Footbridge. She is loud to the southeast from there at 0527. After looking I go back to the eastern curve and get a loud signal to the south. I do not see her. I go west at 0559.

Lizzie has been getting 969 and 994 at Slough and saw wolves near the gap: 994, the black male, two gray pups, and a black pup. They go in the diagonal forest as I arrive at Slough.

I get a glimpse of two blacks and a gray going south through the lawn at 0626.

I go out to the hill and at 0657 see a group of wolves just north of the diagonal forest. It looks like they are greeting an incoming wolf. I see four gray adults, three black pups, and a gray pup. They run into the diagonal forest and I lose them.

I get 969 and 994 at 0725.

I see a collared gray from the fifth lot at that time. It goes out of sight. I leave the lot at 0742.

I head to Hayden from Slough at 0758. I hear that the fog there just lifted.

Deb calls to say she is seeing some of the Wapiti pups.

As I go by Otter Creek, I hear that four adults were seen west of the road in the Alum Creek area. It is now 0842

I stop at Alum and hear that the wolves went out of sight up the power line cut in the trees about a short time ago. I go south from there at 0859 after getting no signals.

I get a good report from Fred. He was on the hill west of Grizzly Overlook and looking north. There was a collared black and the white female west of the road. The other two blacks and the gray yearling followed them. All of them were well west of the road and going east. They swung north and he lost them in the trees north of the Mary Mountain Trail. I wonder if they intended to cross the road and go back to the RS but did not want to deal with the road and traffic.

I hear that a black pup and a gray pup were seen out in the meadow from Grizzly. They went back into the western trees and later another gray pup went that way and was lost at 0820. The first pair did a lot of exploring and playing.

We go up on the hill west of the lot. At 0925 I see a gray pup well east of the western trees. It is going west but a big bison bull is in the way. The pup seems nervous but gets around the bison and we lose it going toward the trees to the west. At 1000 a second gray pup comes in from the northeast and goes into the western trees.

I go back down to the lot and do not get any signals at 1102. I go north at 1111.

I hear that 755 was just seen a mile or two north of Alum Creek, just west of the road. He was going south. I see photos of him.

I stop at the lot north of Alum to look for him. I get a good signal. I do not spot him. The signal is weaker when I go at 1134. The signal fades as I drive north.

I do not get 1014 through Cascade Meadow at 1154.

I get weak signals from 911 and 969 at Slough Creek.

926 is weak from Hitching Post through Soda Butte Valley at 1300.

I talk to Doug in the afternoon and he says four pups have been seen in Mollie’s.

I hear that the white female, 755, and three pups were seen in the main meadow in the evening. The pups interacted with 755 a lot. They wanted to stay with him. They played with him for about 30 minutes. He seemed relaxed and unconcerned about the three black Mollie’s wolves who were not seen.

No wolves were seen at Slough in the evening.

July 28:

It is 48 when I leave at 0451.

I get weak omni signals from 926 from east of Footbridge through the confluence.

I only get 969 at Slough at 0535.

I start looking from the hill at 0542. At 0551 I see the gray male yearling bedded in the lion meadow, uphill from the aspens. He is looking south.

Soon 911 comes in from the south with the head of a cow elk in his mouth at 0610. He stops to look at the gray male to the north who has gotten up and come part way toward him. 911 puts down the elk head and watches the other male with a low head. The yearling has stopped and is also looking at 911 with a low head. They look like they are in a standoff.

The yearling runs to 911 and goes into a submissive posture. Now he rolls on the ground under him. 911 picks up the head and continues north.

The yearling is eating something there so he must have gotten something that fell off the head.

907 comes in from the south at 0619.

911 does a RLU as he goes up the meadow. He goes into the lawn and a black pup and a gray pup run to him. He drops the head and runs from the pups. They chase him. It looks like he stops and does a regurgitation. Then he goes further northeast and we lose him. The pups stay and seem to be feeding at the regurgitation site.

907 arrives in that area and picks up the elk head. She soon puts it down in the lawn. The black pup is at the dropped head and the gray pup with wagging its tail at 907. The gray pup then goes to the black pup. 907 walks off.

We see the black female yearling going up the lion meadow.

The gray male is romping south in the lower lion meadow and seems to be following a scent trail.

After doing a talk I hear that people have seen a gray female yearling.

I go back to the car and do not get any signals at 0734.

I go to the fifth lot and do not see any wolves. I get a faint signal from 994 there at 0740.

I leave the Slough area at 0752 and do not get any signals at that time.

At 0839 I get a weak signal from 755 at the corrals and get him off and on through Otter Creek.

I get to Grizzly Overlook at 0852 and see a gray pup in the meadow. Soon the gray yearling and another gray pup come out of the gully by the western trees. That pup tries to get a feeding from her.

The yearling is soon hunting for insects and rodents.

I do not get any signals here at 0901.

I now have three gray pups.

The yearling reportedly just got a vole and gave it to a pup who harassed her for it.

She and a pup go south through the mud flats and I lose her behind a rise. I continue to see the gray pup. It goes back to the north and picks up a long stringy object and carries it east. It looks back to the west a lot.

We hear that the gray yearling crossed the road south of Alum and went west. It is 0954.

The three gray pups are seen off and on.

I hear that the gray yearling went west, then veered south and went out of sight.

I realize that the four Wapiti pups only have one yearling to play with rather than the six in the Junction pack as well as having less siblings.

Doug flew this morning. I call him and get a report. He got 755's signals from the trees about a mile northwest of the Otter Creek den. Doug did not get 779 or 1014 on the flight. 926's signal was in the No Name Creek area and Doug did not get 965.

I go north at 1115 and do not get any signals through Canyon Junction.

I stop in the office in early afternoon and Doug says there are two wolves in the Cougar Pack with non-working collars: 757 (a seven year old black female) and 825 (also a black).

When I am leaving Gardiner just after 1410, I get some texts Emile about the wolves in Hayden. The white female crossed the road from west to east at 1315 and went to the western trees. The four pups came out and she regurgitated to them. She played with the pups.

The three black males and the gray yearling came in from the west around 1400 and were not able to cross the road. They all did a lot of howling. The males were last seen to the west of Grizzly Overlook going into some trees. Later they were seen going south.

The white female and the pups later went back into the western trees and are out of sight.

755 has not been seen.

I do not get any signals from Junction or Lamar on the way back in.

In the evening we hear that the gray yearling swam the river to the east and went into the western trees. The pups came out of those trees at 1700. The white female came out of the trees at 1933 and the pups ran to her. They went back into the trees.

At 2037 the white female, 755, the gray yearling, and the four pups came out of the trees. 755 played with the pups and seemed to be relaxed. The black males were last seen going south on the west side of the road.

We hear that an uncollared black was seen at Antelope Creek in the morning. I did not get any signals there.

July 29:

It is 48 when I leave at 0453.

I get signals from 969 and 994 at Slough at 0536.

I go out on the hill and at 0603 see two black pups east of the north end of the horizontal forest. One pup goes back west to the trees and the other one goes further east. It runs around and looks back and forth a lot. Then we see 969 northwest of it. She looks around, sees the pups and moves toward it. The pup sees her and stares in her direction like it is unsure. She stops and stares at the pup. 969 then looks around and sits up and scratches. That causes the pup to run to her.

We see the bedded 907 and black male yearling just north of the aspens at the lower right side of the diagonal forest. 907 howls.

I see four black pups and two gray pups in that area. They do a lot of playing and running around.

I hear that the three black males, the two adult females and four pups are out in Hayden. I go that way at 0700.

I call Doug and he says he has also seen 994 here at Slough. I later hear that he also had a third gray pup. A gray yearling came in from the north and joined the others.

I get weak signals from 755 at Alum and get him through Grizzly Overlook.

At 0757 I see the wolves in the meadow from Grizzly. I have all five adults and the four pups. The adults are bedded and some of the pups are walking around.

I get a loud signal from 755 toward the eastern point of trees.

I hear that a grizzly went in there earlier.

1014 gets up and walks west. He does a LFU, then beds. A gray pup goes to him and greets him, then rolls on the ground next to him. The greeting goes on for some time. It is 0810. The pup walks off and does not greet the nearby white female.

The black pup goes to the bedded 1014 and greets him.

A gray pup greets the white female.

I go up on the hill and look from there at 0824.

The limping 1015 gets up and goes west. He does a LFU, probably where 1014 had marked.

The gray yearling gets up and gives the white female a submissive greeting. Then she greets one of the bedded black males.

The white female gets up and goes west. She sniffs where the two males did the LFUs but does not mark the site.

The uncollared blacks goes that way and does not mark the site.

The adults and pups go into the western trees. I see a gray pup off and one at the indentation.

Emile says there has been a lot of howling and bark howling from the western trees.

I see some of the gray pups off and on.

I continue to get a good signal from 755 to the east.

I go north at 1118 and do not get 755 at Alum.

I do not get Junction at Slough or any signals in Lamar.

We later get a report from Barb about sightings in Hayden. The five adults chased a bison calf, then bedded. A pup came out of the Sour Creek RS and went west. Two other pups were near the western trees. The five adults went out of sight on the north side of the Sour Creek RS at 1905. At 1945 755 came out of the western trees. The four pups joined him. He was on the alert the whole time from then on. The pups played near him and he ignored them. A grizzly came out of the western trees and 755 watched it go east. She could still see 755 and three gray pups as it was getting too dark to see at 2115. He was still on the alert due to the bear.

July 30:

It is 49 when I leave at 0450.

I do not get any signals in Lamar.

I just get 994 at Slough at 0536. I see a black pup by the aspens at the lower right side of the diagonal forest at 0538.

I join Doug on the hill and see wolves playing and walking around in that area. I eventually have a collared gray that probably is 994, the drab gray, three black pups and three gray pups. The collared gray repeatedly tosses an object in the air as it jumps up on its hind legs. Some people say it is a dead ground squirrel. I see something in its mouth.

At 0639 I see the black female yearling going north in the lower lion meadow.

I go back to the car and from the lot see the black male yearling to the west.

I head out at 0710.

I hear the five Wapiti adults came in from the south and are now east of Grizzly Overlook. The gray female is leading. Two gray pups went south from the new RS like they knew the adults were coming in but passed by the incoming adults without the pups and adults seeing each other. When the adults reached the western RS and howled, those pups howled, then ran back to them. Soon all four pups were with the five adults.

I later hear that they have gone out of sight into the western trees.

As I get to Hayden, I hear that there has been a lot of howling and bark howling from the western trees.

I do not get any signals at Wapiti Trailhead at 0753.

I see a gray pup to the north of the island of trees from Alum Creek at 0816. It sniffs around a lot. I do not get any signals here.

I go to Grizzly Overlook at 0824 and do not get any signals there.

I go up on the hill at 0832. I get weak signals from 755 to the east/northeast from there, but do not get 1014. We have not had an upload from him since 7/19 and I last got his signal on 7/25.

At 0923 I see a gray pup in the indentation. People have been calling that spot the notch so I will from now on do the same.

Perry calls to say the black male Junction yearling seemed to be tugging and ripping at a site in the lower lion meadow. He then walked off to the north with an adult sized elk leg. I saw photos of him with the leg.

I go downhill at 1041 and head north. I get a loud omni signal from 755 north of the Otter Creek Picnic Area. I pull into the Wapiti Trailhead lot and the signal is much weaker at 1105.

Deb calls to say the wolves have just come out so I go back to the south. 755 is much weaker now where he had been loud just a few minutes earlier. He probably had crossed the road to the west and went on.

I get back to Grizzly Overlook around 1120 and see the five adults, two gray pups, and a black pup wading through the shallow waters of Sour Creek. They are west of the point of trees. Some wolves are drinking there. They continue on to the south.

Both gray pups have dark backs. The black pup has light streaking on its coat.

At one point the black pup leads to the south.

I do not get 1014 and try many more times, but never get the signal.

At times the pups play together as they travel.

The uncollared black seems to be more attentive to the pups than the two older males. The uncollared one has an object is him mouth at one point and the pups run after him. When the other two blacks bed he stays up and watches the pups run around.

The gray female is the wolf most often leading south. The others go single file after her.

At 1148 they reach a section of the Yellowstone River and all the wolves wade in and drink.

The uncollared black has a small stick in his mouth and the gray yearling tries to steal it. She does get it, then romps around with it. Then she drops it but picks it up right away. The uncollared black tries to get it back. She drops it and the black pup now has it. It is 1156.

The gray yearling is leading to the southeast, toward the thick forest. They pass through a small sand pit. At 1207 they are at the edge of the forest. I lose them in those trees at 1213. They were last seen going south.

I go north at 1254.

I get a weak signal from 755 at Canyon Junction and get a much louder signal at the Cascade Lake trailhead lot, west of the junction. At 1314 he is very loud to the north. People in the lot say they saw him in that area, but that he went into the trees. He is still loud when I go at 1324.

I get 969 at Slough at 1443.

I do not get any Lamar signals through the cone at 1510.

I hear that a black yearling was seen carrying a large elk leg north through the lion meadow.

The five Wapiti adults were seen to the east of Grizzly Overlook at 1435 and they were headed northwest toward the western trees. When they got there a black pup came out of those trees. Two gray pups were spotted at one of the sand pits to the east of the lot. They later joined the adults, the black pup, and the third gray pup came in to them.

We hear that the white female went south from the RS at 1713 and came back 44 minutes later with a big belly. She did three regurgitations to the pups. Later we get a report of a bison carcass to the south of Grizzly Overlook with two grizzlies on it. Bruce, a wildlife ranger at Hayden told me that he thought he saw 755 in the RS area and that the other males ignored him. The regular wolf watchers did not see 755.

The five adults and four pups were in the RS in the evening. The gray female went south on the route used by the white female when she was coming back from the carcass. The other adults stayed in the RS.

I also heard from one person that five Junction adults left the den area at 1730 and went west. Another report had eight adults leaving to the west at 1630. The eight pups were seen on the big outcrop on the lower southern side of the diagonal forest.

July 31:

It is 47 when I leave at 0449.

I do not get any signals in Lamar.

I arrive at Slough at 0534 and get 969 and 994.

I go out to the hill and at 0541 see a black and gray at the bottom of the diagonal forest. They go out of sight to the south into a gully. Wolves soon come out of there and we see 969, 994, the black male yearling, the drab gray female, and the gray male yearling.

The eight pups are on the big outcrop on the lower south side of the diagonal forest.

994 and the gray male are playing together vigorously. The gray male throws 994 down.

At 0611 the pups are going downhill toward the adults. The adults go back into the gully below the diagonal forest and the pups go out of sight there.

At 0622 they all come out in the lion meadow. 969 is not with them, just the four yearlings.

A black pup leads south, and the other pups and yearlings follow. It is 0631.

One black pup seems big.

I see a slight smudge on the chest of the black male yearling.

The drab female has a small duck in her mouth and the pups are chasing her to get it. Soon we see that a pup has it and the pup runs around with the duck. The other pups and the yearling chase it.

Some pups continue south to the ford area. Then they turn back and at 0707 a gray pup is leading back to the north.

There are two fishermen along the creek near to where the wolves had turned around. The three yearlings stop and watch those fishermen with high heads, but the men do not see the wolves. The yearlings do not seem to be too concerned. They continue north but look back at the men a few more times. The pups did not seem to notice the men.

They are going back toward the outcrop by the diagonal forest. I lose them heading into the gully on the north side of that gully.

I only get 994 as I leave Slough at 0754.

I hear that the nine wolves were seen out from Grizzly Overlook and that they later went out of sight to the east in the Sour Creek RS. Three grizzlies were at the new bison carcass south of there.

I go through Canyon Junction at 0840. It is about 30 miles from Slough Creek to here.

I get 755 as I approach Grizzly Overlook at 0849. I hear that the wolves have come back out from the trees at Sour Creek.

From the lot I see all nine wolves moving west. The two collared blacks are leading. They and the white female bed in the usual place at 0858. Most of the others bed in that area.

Around 0924 the two collared blacks jump up and run toward the western trees like they might be going after an elk. The white female is running behind them.

I look west of them and see 755 coming out of the gully just east of the western trees. He turns around and runs back to the west and I lose him right away. The two collared blacks go out of sight in that area. They may have been 200 yards behind 755.

A gray pup runs that direction.

The uncollared black was well to the east and now he is running that way.

All the wolves go out of sight toward the western trees.

A few minutes later I see a gray pup in the notch and it seems casual and relaxed.

I go north to Alum at 0938. I get good signal from 755 to the east there.

We do not see anything there. I go back to Grizzly and walk uphill to look at 1024. I still get 755 toward the RS. I do not see anything. I go down at 1055.

I drive to the second lot to the south and go uphill to the west. A man shows me the bison carcass site. No bears are at the site.

755 is weak at Grizzly at 1210 and faint at Alum at 1214. No one has seen anything.

I go north and only get 994 at Slough at 1517.

I do not get any Lamar signals through Soda Butte Valley at 1405.

I hear that in the evening people at Slough saw all eight pup, two gray adults, and two black yearlings in the lower lion meadow. They started near the horizontal forest and went as far south as the ford. They turned around there and went back to the north.

I also hear that 755 was with the pups last evening in the RS and had an interaction with the Mollie’s males. I later get several accounts of the incident.

Perry tells me that the white female came in from the south after feeding on the bison carcass. The three big Mollie’s males arrived and joined her. 755 was west of them. Between 2000 and 2030 the biggest of the males, probably 1015, ran all out at 755 with the other two males following. 755 ran away initially, but not at top speed. He was in submissive posture. It looked like he let the big male catch up. When the Mollie’s black was about ten feet away 755 charged at him. The black moved off, then turned around and 755 backed off. 755 stood there and did a lot of howling. The black male went back east to the other two Mollie’s males and they all bedded with 755 to the northwest. He continued to howl. The three males and 755 watched each other and Perry said there was posturing by all the males. Then a storm came, and he lost visibility.

Fred gives me another account. The five adults had gone south to feed on the bison carcass. They came back through the sand box area in the old RS. He looked north and saw that 755 and the four pups were coming out of the western trees. That was at 1908. They went to the new RS and 755 seemed very relaxed as he hung out with the pups.

The other five adults were lingering to the south and apparently unaware of 755.

At 1920 755 started to go east toward the five adults. The five adults went toward him at a face pace. He continued toward them. It was now 1927. At 1930 two of the black males ran at him, but in a halfhearted manner and pace. 755 retreated from them a short distance from them, then stopped.

The three black males and the two females went to the four pups. By that time 755 was east of them. The white female regurgitated to the pups. Fred could not clearly see if the black males also fed the pups. 755 was howling at that time.

At 1940 the Mollie’s males were bedded with or near the females and pups. 755 was still east of them, about 75 yards away.

One of the black male chased him for 50 to 75 yards. 755 ran from him. The black male stopped and went back to the others and bedded with them. 755 followed him back and ended up within 100 yards of the others. He bedded and seemed very relaxed as he watched the others sleep. They were still in that situation when he left due to a storm. The three males and 755 watched each other and Perry said there was posturing by all the males.

I later got an account from Story. The five adults headed to the bison carcass area around 1640 and got there at 1740. They all fed for 20 minutes, then bedded in the area for an hour. The three males and the white female headed north while the gray yearling did more feeding. Story went back to Grizzly Overlook and heard that 755 has been hanging out with the four pups. She saw them wandering off to the east. The white female and three blacks came in from the south at 1920. As they got closer the Mollie’s males sprinted at 755 and ran away from them. The female had been running with the males but broke off to go to the pups. Two of the blacks were leading the chase and apparently the third one lost interest soon after the chase began. The second of the two broke off the chase and the third black male continued to run after 755. He got close enough where he could have made contact with 755 but did not. The black turned around at that point and headed back toward the pups and the white female. 755 trotted after him closely and bark howled repeatedly. The black turned around and charged at 755 a few times. 755 ran off a bit each time, then continued to follow him when the black Mollie’s moved on. That sequence lasted five minutes.

The black rejoined the other two males, the white female and the pups and they had a rally. 755 watched and continued to howl. He bedded about 50 yards from the others. At times he stood up to watch them. At other times he bedded or sat up as he monitored them. The others bedded. They all stayed that way until a thunderstorm came in. 755 got up and trotted off to the east, away from the other wolves and went toward the western trees. That was around 2040.

I get another report from Marisa. She had the five adults go south to the bison carcass. They later came back. She saw 755 in the main RS with the four pups. 755 spotted the five approaching from the south before they saw him. The white female saw 755 and ran toward him but the three black males ran with her. She stopped. Then she ran to the nearby pups. 755 was running north, then ran east. He stopped north of the mud flats, looked at the black males and howled. The three Mollie’s ran at him. His tail was tucked, and he was in a crouch as he ran sideways away from them. He ran off further, then paused. The Mollie’s stopped. The biggest male ran at him by himself and 755 went into a submissive posture. When the black got close 755 changed his posture, jumped at the black and seemed to make contact. The black backed up and 755 stopped. The big black retreated. She said in summery that 755 stood his ground and the other male moved off from him. The black went west but did not rejoin the others. 755 did a lot of howling and looked at the white female and the pups. The pups tried to go to 755. She thought that the big male was working to prevent the pups from going to 755. He did a lot of staring at 755. 755 bedded. The storm came in and during it 755 got up and moved toward the other adults, then backed off several times. Later he went out of sight in the trees in the old RS to the east.

I last got signals from 965 on 7/21 and last got 890 on 7/22.
