# Supplementary material for: Breeding displacement in gray wolves (Canis lupus): Three males usurp breeding position and pup rearing from a neighboring pack in Yellowstone National Park
Source: PLoS One. 2022 Nov 30;17(11):e0256618. doi: 10.1371/journal.pone.0256618 (PMC9710779; doi:10.1371/journal.pone.0256618)
Supplement: S2 File — (DOCX) [file pone.0256618.s002.docx]

WOLF OBSERVATION NOTES

August 2016

by Rick McIntyre**

On the morning of 7/1 the black male yearling following the scent trail of the pups to the meadow below the diagonal forest, then continued north. In the evening four of the adults and six of the pups went south from the den area and the pups ended up south of the two diagonal Christmas trees, the furthest south they have been. Then they went back to the north with a pup leading.

On the 2^nd^ I saw 926 and 965 in the Institute area. They reportedly had been chasing coyote pups in that area. In the evening six pups followed two adults to the diagonal forest and later came back to the den area. The two adults continued on to the north.

All eight pups were visible on the 3^rd^. 969 came in with the hindquarters of a pronghorn fawn and kept the yearling from it so that the pups could eat. But she then walked off and yearlings and pups fed side by side. In the evening the adults and pups looked toward the direction of sirens on a ranger vehicle and howled back at it. Four adults started to leave on a hunt, but 969 and the male gray yearling continued on while the two gray female yearlings ended up bedding by the stump.

On the 4^th^ we saw 890 and the limping female going south. 890 later came back and regurgitated to the pups. The black female yearling came back to the den with an elk leg and we later saw the black male yearling to the west with a bison leg. He put it down and returned to the den without it. The three Lamar wolves were spotted south of Footbridge and later went out of sight into the trees at the Chalcedony fan.

A number of the Junction wolves were in the flats to the south of the den area on the morning of the 5^th^. The black male yearling was there, and he carried an elk leg from that area all the way up to the den. Four of the pups, 969, and two yearlings went south and turned around downhill from the aspen pass. That is the furthest to the south that the pups have been. They were just slightly above the lower lion meadow at the time. All the adults went back to the den area with the pups. After getting back the other four pups went downhill to the upper part of the lion meadow by themselves. 911 came in and did a regurgitation. I saw all the adults and all the pups that morning. On the evening of the 5^th^ 911 did four regurgitations to the pups.

Seven of the pups and a number of adults went north of the horizontal forest on the morning of the 6^th^, then came back to the den. That is the furthest the pups have been to the north.

890 went north, west of the horizontal forest, on the morning of the 7^th^. I had 969 and pups by the gap in the diagonal forest. The three Lamar wolves were seen north of Dorothy’s in the morning. Melba reported that seven Mollies wolves were at the Sour Creek RS in the morning: four blacks and three grays.

On the 8^th^ 890, 911 and the black male came in from the south. I saw all 18 Junction wolves that morning. We hear that the three Wapiti adults, a black pup, and a gray pup were seen at the Sour Creek RS in the evening. Later in the evening six Mollie’s wolves including 1014 were in that area. There were four blacks and two grays. They came out of the western trees and bedded halfway toward the point of trees. Another report suggested that the two gray may have been the Wapiti alpha female and the gray yearling. They were apart from the four blacks.

I had pups and adults at the Junction den in the morning of the ninth and heard that four Junction adults were spotted south of Dorothy’s while I was at Slough. I only saw five pups and no adults at the den in the evening. We heard that three black Mollie’s wolves chased the Wapiti alphas in the early morning. The Wapiti alpha female split off and the Mollie’s continued after her. Later the three Wapiti adults got back together. They crossed the road and went west. The Mollie’s went south and veered into the trees. In the evening the white female and two pup were back in the RS. Two of the Mollie’s were collared and likely were 1014 and 1015.

On the 10^th^ I had eight pups and five adults at the Junction den. Melba reported saw the two Wapiti females with the three black Mollie’s by the sand pit. 755 was seen alone east of the road. He crossed to the west. Earlier the gray yearling was seen crossing the road near Alum Creek.

We had 22 horses and mules go through the den area on the morning of the 11^th^. Three riders passed within 100 to 200 feet of the sage den on the way to gather up the stock then came back and passed close the site again leading the animals back. In the evening I saw eight of the adults on the north side of the diagonal forest and others saw seven pups there. A video was taken of two black Mollie’s interacting with the two Wapiti females. One of the blacks was collared and reportedly was 1014. Earlier only one female was with them. The males and females were very playful and were flirting with each other. No Wapiti pups were seen. 755 was spotted at 2230 crossing the Chittenden bridge to the east.

The Junctions were mostly in the diagonal forest area on the 12th, near or in the gap and lawn. I did not see any wolves at Slough in the evening. The two Mollie’s males were seen with the two Wapiti females in the afternoon. Lizzie did not get 1014 so we wonder if the collared male is 1015. The other black male was uncollared. Lizzie got 755 from the RS in the early morning but it was too foggy to see anything. His signal dropped off before it cleared.

On the 13^th^ the Junctions were in the diagonal forest and south of it. I also had them on the lower right side of the forest, an area that they later spent some time at. The two Wapiti females and two black Mollie’s were seen by Melba at the sand pit area north of the bridge around 0600 and in Cascade meadow at 1130. They were going south. 755 was seen west of the road and had trouble crossing to the east due to traffic. We saw photos of 755 traveling with the four pups in the evening. They were east of the Alum Creek lot on the other side of the river. That would put them near the western trees. There were three gray pups and one black pup. Later 755 and two gray pups were going south from the RS, then going back to the north. He did a lot of howling.

We had the Junction adults and pups going north from the diagonal forest on the morning of the 14^th^. I went back to the east and saw the three Lamar wolves near the confluence. When I later returned to Slough Doug told me that 911 had turned around in the yellow grass meadow and took the group back to the diagonal forest. I saw the wolves in that area in the evening. The two Mollie’s males and two Wapiti females were in the RS at 1430. They later went south to the Nez Perce Picnic Area and probably had a carcass there. At 1800 the two males and the white female came back from the south and looked full. The female had blood on her face.

On the 15^th^ some of the Junctions were near the south end of the den ridge. 969 was with the pups in the lion meadow. She took them north and up the north side of the diagonal forest.

I went down to Hayden and saw 755 and the gray yearling coming back from the south from a lot south of Grizzly Overlook. She may have brought him to a likely carcass at Nez Perce. We lost them going north. I went to the Wapiti Trailhead to look for them. After I left the two black Mollie’s wolves were seen chasing 755, but in a halfhearted manner. The white female was lagging behind those two males. Photos of the collared black indicated that he was 1015. I did not get 1014. 755 ran off to the north then stopped to look back. The Mollie’s did not pursue him further. Photos of the incident showed that both Wapiti females were with the two black males. At 2015 the white female was seen south of Mud Volcano on the west side of the road. She howled for 30 minutes and there were answering howls from east of the river. She crossed the road and river and was seen with two blacks. They went north at 2100.

I had Junction wolves in the lawn and gap area on the 16th. Carl saw two black pups with a black adult at Blacktail in the morning and in the evening, Emile had two gray pups there. I went to Hayden and from Grizzly Overlook see three black Mollie’s including 1014 and 1015 with the two Wapiti females. They do a lot of interacting and flirting. All three males did RLUs. The white female did a FLU at 1014's RLU. The five later went south and tried to get elk from a big herd of cows and calves. We think that they did get a calf in a gully. The white female was later seen going north with a full belly and even later came back from the south with an elk leg. We think that the pups are in the western forest, the one on the east side of the river north and south of Otter Creek.

On the 17^th^ I had the Junction wolves in the lion meadow including the eight pups. 994 and the gray male yearling chased a pronghorn fawn for a long distance. I went to Hayden and did not see any wolves. The fine adults were seen in the evening, but not any pups. On the way back from Hayden I got signals from 911 and 969 at Antelope Creek. I hear that two wolves were seen at a carcass in this area yesterday. The signals are coming from that area.

I had the Junction adults and pups go to the yellow grass meadow to the north on the 18^th^, then saw them veer off to the northwest. Later they started to back to the den area. I went to Hayden and had the five adults to the east of Alum Creek. A grizzly reportedly pulled an elk carcass into the western trees in that area and the wolves went in and out of that area. People heard a wolf howling from the trees to the east of the five wolves and the three Mollie’s males got up and went that way. Two soon stopped and went back to the females. A collared black traveled all the way across the meadow and went into the trees near one of the sand pits, then later came back out and returned to the others. People thought that the howler could have been 755, but Lizzie did not get his signal. He might have been bedded behind a hill and had his signal cut off.

I had two yearlings and four pups at Slough on 7/19. A yearling and a pup went to the sage den. I did not see any wolves in Hayden. I later checked 1014 points and he was at a likely carcass site to the west of the road in the Elk Antler Creek area. I heard that two blacks were briefly seen in the RS early in the day just as the fog was lifting. Quinn flew that day and got 755 two kilometers southeast of Wrangler Lake. In the evening he was seen crossing the road to the east near Alum Creek.

On the 20^th^ I had seven adults and seven pups going south at Slough. The pups went down to the water in the western bend of the creek. The wolves went up the aspen drainage, then came back through the aspen pass. All five adults and four pups were seen at the Hayden in the morning and in the evening. 755 was not spotted during the day, but in the evening was seen in the NPS employee area near Canyon Junction.

I did not see any wolves at Slough on the morning of the 21st. Three were seen after I went to Hayden. I saw the five adults and three of the four pups there. We also saw 755 to the northeast. In the evening I had three Junction adults and seven pups leave the diagonal forest and go all the way to the southern round tree. This was the last day I got signals from 965 for some time. The next day I go his signal was on 8/4. The five Wapiti adults and at least three pups were seen in Hayden in the evening.

On the 22^nd^ I had three adults and eight pups at Slough. That was the 100^th^ morning we saw wolves at Slough. I went to Hayden and saw the five adults and three of the pups in the new RS. Other people saw all four pups. In the evening 755 reportedly came out of the eastern RS and went toward the western RS. He came near to a gray pup, but they did not see each other. Then 755 went into the western trees. We later heard that the three black males chased him, but not too aggressively.

That was the last day I got signals from 890 or saw him. Erin later got his signal and possibly saw him with or near the Mollie’s wolves in Pelican Valley on 8/5.

I had three Junction adults and the eight pups at Slough on the morning of the 23^rd^ and saw the five adults and two pups in Hayden. We saw 755 well to the east of the new RS and he ran off to the east. Other people told us that two of the black males were in sight at the time and probably 755 was running from them.

On the 24^th^ I had five Junction adults and eight pups. The pups are based in the area o the lower right side of the diagonal forest. In Hayden I had 755 with the four pups. He later crossed the road to the west and went north. The three Mollie’s males and the two females came in from the south after that. We wonder if 755 somehow knew that they were returning.

I saw five Junction adults on the 25^th^ and the eight pups. Emile had two gray pups and a black pup with 763 at Blacktail that morning. I only saw the gray yearling and one gray pup at Hayden. I got loud signals from 755 in the Cascade Meadow area west of Canyon Junction and people saw him to the north in that area before I arrived. He was going north. In the evening all five adults and four pups were seen.

I had three Junction adults and seven pups at Slough on the 26^th^. I went to Hayden and saw the five adults and four pups. Those adults went off to the north. 755 came in from the north, then went into the western trees. The black males later came back and followed his scent trail there. The white female went that way. We saw 755 going east, north of her, and lost him going past the eastern point of trees. The white female and the three blacks followed his scent trail that way. Carl saw 755 north of there and we saw the white female follow his route without the black males. Later she and the males went back to the western trees. 755 was seen after that swimming the river to the west and going north in the Otter Creek area.

On the 27^th^ I saw four adults and four pups at Slough. When I got to Hayden, I heard that the group of five adults were seen west of the road. They went out of sight to the north. I saw two gray pups in the RS and other people also saw the black pup. In the evening we heard that 755, the white female and three pups were in the RS and all of the wolves were relaxed. The pups seemed to want to stay with 755 rather than with the white female.

I saw four adults and two pups at Slough on the 28th. 911 came in from the south carrying the head of a cow elk. He went to the lawn and dropped the head there as pups ran to him for a greeting. We later saw the pups feeding on that head. In Hayden I had the gray yearling and three pups in the morning. We heard that later the yearling crossed the road to the west and continued west. In the afternoon Emile saw the white female come in from the west and cross the road to the east where she regurgitated to the pups. After that the yearling and three black males also came in from the west but could not cross the road. They did a lot of howling. In the evening the gray yearling was seen swimming the river to the east. She went to the RS and joined the pups and the white female. Later people saw 755 with the two females and four pups and all of them seemed relaxed. The three black males were last seen going south, west of the road.

On the 29^th^ I had three adults and six pups at Slough. I went to Hayden and saw the five adults and four pups. I got good signals from 755 toward the eastern point of trees. In the evening the five adults reportedly chased a bison calf into the western trees. The adults later went east and were lost behind the eastern point of trees. After that 755 and the four pups came out of the western trees. 755 was on the alert as the pups played. He watched a nearby grizzly.

I had four adults and six pups at Slough on the 30^th^. I heard that the five adults had come in from the south in Hayden and went back to the new RS. I went down there and saw the five adults and three pups. I have not gotten 1014 since 7/25 and his collar has not done any uploads since 7/19. We wonder if it has stopped working. The five adults later go back to the south and we lose them in the trees to the east. We hear that there is a new bison carcass to the south and they probably went there. Grizzlies have been on that carcass. I got loud signals from 755 at Cascade Meadow when I went north and talked to people that saw him to the north of the trailhead lot west of Canyon Junction. I later heard that the white female went back to the south and came back with a big belly. She regurgitated three times to the pups. In the evening the five adults and four pups were in the RS. The gray yearling went south on the route that the white female had come in on. I later got reports about the Junction wolves in the evening. Either five or eight adults left and went west, and all eight pups were seen.

I saw all eight pups and five Junction adults at Slough on the morning of the 31st. They went south an adult and later a pup ran around with a duck in their mouths. I hear that in the evening eight pups and four adults were seen. I went down to Hayden in the morning and saw the five adults and four pups. Two of the black males ran toward the western trees from the east and I saw 755 there. He turned around and ran in to the trees with the blacks running after him. In the evening the five adults went south to the carcass. While they were gone people saw 755 in the RS with the pups. The five adults came back to the RS at 1920 and briefly chased 755. 755 reportedly stood his ground when the biggest of the blacks came at him and actually charged at him. The big black retreated from him. Later 755 bedded about 75 yards from the others and did a lot of howling.

August 1:

It is 42 when I leave at 0459.

I do not get any signals in Lamar.

It is 23.6 miles from my cabin to the Slough Entrance Road.

At 0543 we see wolves to the west of Bob’s Knob in the lower part of the lion meadow. I soon have all eight pups, 911, 994, and the drab female. 911 is still limping.

Lizzie is only getting 994.

The pups continue south and soon are near the ford. The drab female is with them. The pups are sniffing around the ford area. 994 joins them.

We see the black male yearling coming in from the south. He goes up the lion meadow without seeing the pups or other adults to the east. I see that he is carrying a tidbit. He passes the bedded 911 who is east of him. The black veers up the ridge and continues north toward the den area.

Some of the pups wade out in the shallow water and drink.

I go back to the car and at 0618 only get 994.

I leave Slough at 0623 and go through Tower at 0642.

Doug later tells me that pups went to 911 and he seemed grumpy with them.

I get omni signals from 969 from the Gut Road through the hairpin turn in the Antelope Creek area.

I get weak signals from 755 at Alum at 0725. The river area is fogged in, but everything else is clear.

I park at Grizzly Overlook and go up on the hill to the west at 0736. The RS is fogged in and I have to wait for it to clear.

I heard howling on the way uphill.

Perry joins me and says there has been a lot of howling and bark howling all morning. The howling sounded mournful and people wondered if it was from 755.

It clears and I see wolves in the main meadow at 0803. I have the three black males and the gray yearling. The males are bedded. The white female and two gray pups are walking around east of the males.

The gray yearling romps around, then does a lunge like she is going after a rodent. She later goes into the gully as she heads toward the western trees. The uncollared black male follows. I lose them both in the trees.

At 0808 what looks like 1014 gets up and howls.

I see the third gray pup in the notch section of the western trees.

1014 continues to howl from a bedded position. He is looking toward the white female and the pups as they are coming toward him.

I see that one of those gray pups is much smaller than the other one. People tell me that the bigger one did a LFU.

1015 is up and limping toward the white female. He stops and howls. She and the two pups look at him to the west. Then she beds facing him.

The small gray pup continues west, then stops to look at 1015.

1014 gets up. He is west of the other visible adults.

The small pup runs to him. 1014 is bedded when the pup arrives, and the pup gives him a submissive greeting. It licks his muzzle and paws at his face with a front paw. The male tolerates the greeting.

The white female is up and looking at them. She goes toward 1014 and the small pup. The big pup follows her. 1015 is up and looking that way. Then he also goes toward 1014.

The small pup is still greeting 1014 as the white female and 1015 approach. The big gray pup has stopped and seems to be hesitating.

The female goes past 1014, then stops and stares toward the western trees, like she is seeing something there. She beds there and continues to look west.

I see a gray pup in the notch.

What is probably the big gray pup goes west and I lose it in the gully heading toward the forest.

The two collared blacks and the white female are still bedded.

At 0907 I get a good signal from 755 and he seems to be toward the old RS to the east.

The white female gets up at 0909 and walks by the bedded 1015. He does not lift his head to look at her and she does not go to him.

I now have the black pup and a gray pup in the north.

I see the gray yearling and uncollared black coming in from the north at 0930. She goes toward the western trees and he veers off to the east. Soon he goes back to the west and I lose him on her route toward the trees.

The other three adults did not notice them.

I see the black pup leave the notch and hear that the gray pup did as well. They are probably going to the two incoming adults.

The other two black males and the white female are up and going toward the western trees. The males are leading. I lose them heading that way. Then I see one of the collared blacks in the notch. He runs west through that area.

At 0941 1015 and the white female come out of the western trees and go east. The other two black adults and the gray yearling follow.

The two collared blacks sniff around a site east of the trees and probably are getting 755's scent. They move off to the north on the route used by the gray yearling and uncollared black earlier. The white female follows. They encounter a bison herd and some of the bison charge at the wolves.

At 0949 the gray yearling and uncollared black follow the others on that route. I lose all of them to the north.

I go north at 1000 and get weak signals from 755 at Alum. His signal is better at the Otter Creek meadow and I also get a good signal from him in the Wapiti Trailhead lot. We wonder if there is a carcass in the area.

I go south at 1034.

He is weak at 1057 at Grizzly Overlook. I do not get him at 1111.

I do not get him at the trailhead lot at 1119.

I do not get any signals at Slough and do not get any signals in the Lamar den area at 1241.

Lizzie later tells me that she lost the eight pups at Slough going up through the gully on the north side of the outcrop on the lower left side of the diagonal forest. The yearlings had gotten them turned around and led them north.

It is 81 when I leave at 1723.

I do not get any signals in Lamar or at Slough at 1804.

I look from the knob at 1806 and do not see anything. I go the fifth lot and at 1855 see a gray pup at the lower right side of the diagonal forest.

I go back to the entrance lot and see a black pup in that diagonal forest site at 1911.

I do not have any signals when I leave at 1919.

I get a good signal from 969 toward Jasper Bench from Coyote. I look, but do not see her.

I do not get any signals at Hitching Post.

I hear from Fred that the five adults and three of the pups were visible at Hayden this evening. 755 was not seen.

August 2:

It is 48 when I leave at 0457.

I see two snowshoe hares on the road west of Upper Baronett.

I do not get any signals in the Lamar den area at 0521.

I get 994 from Dorothy’s to Coyote.

I do not get any signals at Slough at 0537. I go out on the hill and at 0544 see a black pup and a gray pup in the lower lion meadow. They are running north up the meadow and at times go at a run. I lose sight of the gray. The black is soon in the upper part of the meadow. I then see that the gray pup is following the black. We lose them as they go into the sage on the south side of the lion meadow. They are probably going back to the diagonal meadow.

I see another gray pup at the lower right side of the diagonal forest.

I leave Slough at 0655.

I do not get 755 at Alum.

I get him at Grizzly Overlook. At 0751 I see the five adults in the western RS. No pups are visible.

Two of the black males are going west at a trot. I look west of them and see 755 in the sage east of the western trees. He turns around and runs west at less than full speed, then stops and looks back at the two males. 755 goes west again and stops to look back once more.

The lead two males are not running at him but are more at a trot. The gray yearling is between them. The third black male and the white female are further to the east and just looking toward the others.

755 is staying in the same place. He beds and howls a number of times. A big bison bull is between him and the others. The lead black male and the gray female are looking at him.

The white female howls from behind the males.

The lead black male beds.

I go uphill to our new observation spot.

When I look out at 0803, I see that 755 is up and moving off to the southwest with a bit of a limp. The gray female is northeast of him. 755 looks at her as he runs past her. He is now going east and looks back a lot.

None of the males are watching him as far as I can tell. One of the males is bedded where 755 had been and another black is playing with the gray female. The white female is bedded.

Bonnie tells me that as I was going uphill the blacks were going toward 755 at a walk. He got up and skirted around them. The blacks did not chase or follow him. That was when I picked him up again uphill.

755 is moving back to the west a bit. He looks west and is on the alert. 755 beds at 0813. He does a lot of howling.

The gray yearling is near two of the black males. They both get up. I see a gray pup near them.

A black pup is also there. It goes west and the gray pup follows.

Later two black adults and the gray female go north. I lose sight of one of the blacks, a collared one. The other black is uncollared black and he does a LFU. The female sniffs the spot and does a SU there. Both continue north through a marsh and I lose them.

755 is bedded and watching the wolves from the east. He gets up and is still on the alert. 755 moves off to the east, then comes back. At 0830 he is going east again at 0830. I lose him going behind the point of trees above the cut bank at 0832. He was not limping when I last saw him.

I see two grizzlies on the bison carcass to the southeast.

The gray yearling is back in the new RS and she is interacting with a black male and pups in the thick sage. It looks like there are two gray pups and a black pup.

I see a collared black going north.

A gray pup and the black pup go out of sight toward the western trees.

I see 755 coming back out of the point of trees at 0910. He is going at a fast pace and has a high head. 755 stops and looks toward the other wolves at 0913.

He continues to look toward the others.

At 0920 the white female gets up and looks west.

755 trots off a bit. He stops and looks back. I think he should be seeing her.

A black male is just east of the white female. It is 0922.

755 is standing and watching them. He beds and continues to look that way. At 0926 he gets up and moves off to the north.

Both collared blacks are going west now. Lizzie sees the third black. A gray pup is visible in that area. We have lost the white female.

We have lost 755 to the north.

At 0940 we see him again and notice that he is circling the general area rather than leaving.

We have seen all four pups.

755 is now well to the north near the mud flats. That is where we earlier had the gray yearling and uncollared black. I see him rolling at what looks like the spot marked by the black male and gray yearling. He rolls again and sniffs around the spot. I did not see if he did a scent mark there. I lose sight of him after that.

I see the gray yearling coming in from the north to the berm in the new RS. A gray pup is following her. The uncollared black male is following her and his tongue is hanging out. It is 1004.

The gray goes west through the gully toward the western trees.

The uncollared black stops and does either a LFU or RLU.

Then he goes by the gray pup. The pup runs to him and tries to greet him in the face. The male does not respond, and the pup moves on and follows the scent trail of the female

I lose them in the gully going toward the western trees.

At 1018 the uncollared black comes out of that area and goes east. The yearling also comes back out. I lose the black male heading north again. He was near where 755 was rolling on the ground. There may be a carcass to the north of that area.

The two other black adults follow the white female toward the western trees. The uncollared black comes back from the north and joins them. They all go out of sight toward the western trees at 1029. The gray yearling went there as well.

At 1050 two gray pups are in the notch.

I go downhill at 1213 and do not get 755 at the lot. I head north at 1215.

I do not get him as I drive north. He is weak as I go by the corral area at 1227.

I do not get any signals at Slough at 1327. I get a weak signal from 994 at Dorothy’s.

I get a good omni signal from 926 from Picnic Area through the den area. She was best at 21's crossing. I do not get 965. I continue to get her through the cone, then she drops off. She is probably up high.

Later all three blacks are together again

Doug later tells me that the Junction black male yearling crossed the campground road near the third lot and went out of sight behind the horizontal forest.

I hear that in the evening at Slough two black pups and two gray pups went to the Simpson tree area. They were visible for two hours. I get a secondhand report of wolves getting a bison calf by a bridge last evening. That may have been the Lamar bridge.

I later hear from Fred that 755 showed up at 1900 in the evening and went toward the western trees. He bedded in the RS at 1928. The three Mollie’s and the white female came out of the western trees at 1931 and 755 got up and retreated to the east. Two pups went toward him, as did the two females. 755 continued east as the pups went toward him due to seeing the black males behind the pups. One of the Mollie’s had a brief slow chase of 755. He ran off but stopped when the black stopped.

The adults and pups got back together and moved off to the northwest and 755 followed from about 200 yards behind them. The adults continued north, and Fred lost sight of the pups. 755 went into the western trees at 2002.

August 3:

It is 52 when I leave at 0502.

I get variable signals from 926 from Round Prairie through the confluence. They are best at Footbridge and seem to be to the south from there.

I get signals from 969 and 994 at Slough at 0546.

We see three black pups and a gray pup in the lion meadow. They do some howling.

I now have three black pups and four gray pups out there. I also see the bedded 994 south of them. He is looking south. 994 gets up and stares ahead with a fast wagging tail, then does a pounce for a rodent. I see him eating something, probably a vole.

Later I see one of the three black pups going back to the aspens at the lower part of the diagonal forest. Other people saw the fourth black pup there.

I do not get 969 when I leave Slough at 0700.

Deb is in Hayden and she reports seeing an uncollared black going south through the RS area. She also has the three Mollie’s blacks in sight. Those blacks and the pups reportedly went into the western trees at 0715.

I get 755 at Alum at 0749.

I go to Grizzly Overlook and at 0752 see the five adults in the RS. One of the blacks is doing a LFU. Deb had the four pups out earlier.

A collared black playfully jumps on the back of the gray yearling.

Two of the black adults (1014 and the uncollared one) play together and the gray female watches. At one point the uncollared black playfully pins the older 1014. The gray comes over and joins in on the play. 1015 joins them and the three blacks and the gray all play.

I get a good signal from 755 at 0800.

At 0806 two gray pups and a black pup come in from the western trees. The pups go toward the bedded adults to the east. The adults have their heads up and are watching the pups. Two gray pups run to the white female, then one of those pups goes to the males. The second gray pup also goes to the males. That pup greets one of the bedded collared blacks for some time. That pup walks off and bypasses the other two blacks and greets the gray female. The black pup has not greeted any of the adults.

Some of the adults come back. I see the gray female running at two gray pups. The uncollared black runs at a black pup and the pup drops down and rolls on the ground under the adults. The gray female is playing with two gray pups. The two collared blacks and the white female are bedded nearby.

The uncollared black runs to a gray pup and pins it. The pup rolls on its back under the adult.

The gray yearling chases a gray pup toward the gully. A second gray pup is there. The black male runs after those wolves and all of them go into the gully. I lose them in the western trees at 0820.

The two collared blacks and the white female are bedded east of there.

The gray yearling and uncollared black come back out.

Deb tells me that the unknown black crossed the river at Alum Creek from the north and went south through the RS toward the bison carcass. She said it was an adult black male with a big blaze. He seemed comfortable about going through the area.

The gray yearling playfully runs at the uncollared black and he dodges her. They go to the other bedded adults. The uncollared black has a lot head as he greets a collared bedded black, then rolls on the ground next to him. I now see that this is 1015.

The two females get up and interact with the uncollared black. The gray female chases him in circles.

1015 and the white female are now going east.

At 0840 the wolves are going to the mud flats southeast of the RS. But 0845 it looks like they are going south toward the bison carcass. I hear that four grizzlies have been at the site. The wolves wade the shallow creek with the gray yearling leading.

A collared black pins the uncollared male.

The gray leads uphill from the flats to the southeast and is sniffing at a scent trail that she seems to be following. The others sniff at it as well.

The gray female is at the edge of the trees and leading south as she continues to follow the scent trail. The black male follow her. The white female runs to catch up with them.

The pups have stayed behind in the RS.

All the adults stop to sniff at a spot, and I wonder if they are getting the scent of 755.

We then lose the five wolves going into the thick forest there. They were still going south at the time.

I look north at 0927 and see 755 going toward the distant mud flats to the north.

I hear that the adults are at the bison carcass, then get a report that the bears are keeping them from the site. They give up and leave.

At 0934 755 goes out of sight to the north.

I see the gray yearling by the bison carcass. She now is going back to the north. I hear that the other four adults are going south.

At 1010 the gray is heading into the western trees via the gully. The pups come out and it looks like she does a regurgitation. There are two gray pups and the black pup. They soon go into the western trees.

I get a good signal from 755. At 1036 I see him coming back from the north. He veers over to the eastern point of trees and I lose him there at 1104. I get his signal toward the sand pits at 1121. I still get him when I leave at 1129.

It was 52.8 miles to Grizzly Overlook from the NE lot.

I do not get any signals at Slough at 1232. I get weak signals from 926 at Mid-Point at 1244. She is also weak in the den area.

Doug later tells me that 907 and the black female came in from the Simpson Tree area. The pups were in the upper lion meadow at the time. The two females greeted 994 in the lower meadow. A black pup saw them and ran all the way downhill to them, but the incoming females ran past that pup and went to the lower right side of the diagonal forest where they went out of sight. They may have fed the pups there.

Laurie later called and passed on a report from Fred about events in the evening at Hayden. 755 and the pups were together in the RS. The three black males and the two females came in from the east. They bedded for an hour with 755 being 75 yards from them. He did a lot of howling. Later he walked around sideways to the males with a neutral tail, then he bedded. The other five adults were bedded together.

I later get a report directly from Fred. At 1800 he saw 755 and a gray pup in the notch. There were more sightings of pups there over the next hour. At 1938 755, the yearling, and the four pups, came out from the western trees and went east into the new RS. They all bedded there. At 2015 the white female and three blacks came in from the eastern sand box. 755 got up and approached them sideways. His tail was not tucked. One of the blacks briefly chased him and he ran off a bit. They got to within ten years of each other. The black stopped and 755 stopped. Then the black went back to the other adults and they bedded. 755 did a lot of howling. All were bedded by 2020. 755 was about 50 to 75 yards from the three males. The white female and 755 did not get together.

August 4:

It is 38 when I leave at 0452.

I get a good signal from 926 at Footbridge at 0517 and between the lots. She gets weaker by the time I get to the Exclosure Fence.

It gets down to 33 in Lamar.

I do not get any signals at Slough. At 0618 we see the black female yearling down the lion meadow. Later she goes back to the horizontal and diagonal forests.

I leave Slough at 0718.

Deb tells me that she had seen the white female and others to the east of Grizzly in the fog at 0630. They went south. The lighting was poor and hard to tell who was in the group, but she thought the three black males were with her.

I get good signals from 755 at Alum and at Grizzly at 0814. I hear howling.

I see the four pups to the northeast of the lot in the flats. Then I see 755 and the gray yearling bedded on a low hill southeast of them. They are watching the pups.

At 0823 he goes to the four pups and the gray yearling follows him. 755 sniffs the nearest gray pup. He beds and watches the pups walk around to the north of him. When the pups move off further to the north, he gets up at 0832 and follows.

The pups wander around, but gradually move northwest toward the new RS. 755 repeatedly gets up to follow them, then beds and watches them. The gray yearling also follows them. 755 seems to on the alert at times. It is 0851.

At 0854 he gets up and follows the pups again. 755 stops at the nearest pup and it rolls on the ground under him. Now he seems relaxed. He looks around at times, probably for the black males.

Now 755 is following a gray pup. He beds up on the berm and continues to watch the pups to the west. There is a pattern where the pups continue to move toward the new RS and as they get further away 755 gets up, gets closer to them, beds, and watches them, then gets up again and follows as they move on. It is like he is monitoring them. At one point he stands up and watches as he stays in place.

The black pup comes back to the bedded 755 and greets him. That gets him up.

The pups now seem a bit on the alert.

The pups and yearling are now hunting for insects and rodents. She does a run and pounce. 755 just watches them.

A gray pup does a LFU. The black pup does a SU.

755 is now standing and looking east and southeast, likely for the Mollie’s males.

At 1014 he looks toward a pup to the east of him, then toward the western trees. He beds and the gray pup greets him in the face.

755 gets up three times, walks around each time and then beds on the west side of sage clumps each time. That would put him in a little shade and would also give him a clear view of the pups to the west of him.

Three pups have gone out of sight into the gully. They had been moving toward the western trees. The fourth pup goes out of sight that way at 1035.

I lose sight of 755 in that area.

He is weak when I go north at 1125. I also get him at Alum.

I go to Gardiner and head back to the park at 1515.

I get back to Grizzly Overlook at 1817 and hear that 755 had come in from the northwest and went into the western trees at 1720. I get weak omni signals from him.

I look from the hill at 1835. The four pups come out of the western trees at 1940. They play aggressively with each other. Three pups have one gray on the ground and go after it.

I hear that the gray yearling has been chasing elk in the Alum Creek area. At 1951 I see her heading east toward the pups. 755 is with her.

They look toward the east end of the western trees and must be looking for the pups. The pups are east of the trees and they do not see the adults. The two adults are going toward the western trees. 755 stops and looks south, probably for the Mollie’s males. He looks around the RS area for the pups, but does not see them, then he look south again.

Suddenly he raised his tail and runs aggressively at the pups. I think he saw the black pup and mistook it for one of the Mollie’s blacks. The yearling also runs that way with a raised tail. The pups scatter like they are scared. Two run past the two adults toward the trees. They must have mistaken their own adults for rival wolves.

Now three of the pups run to 755 and try to get regurgitations from him. The fourth pup must have been too scared to realize who the wolves were. I do not see 755 feed the pups. They go to the yearling and pester her for a feeding, but do not get one. They roll on the ground and try to touch her face.

The pups are now totally concentrating on getting a feeding from the yearling and are leaving 755 alone. As she interacts with the pups he is looking around, like he is guarding the family. The female runs off and the pups chases her. 755 beds and looks south. The yearling is now playing with the pups.

755 beds close to the pups. They harass him and get him up. He beds as the pups play together within a few lengths of 755.

There are two gray pups and the black pup.

I lose all of the wolves in the gully. A bit later I see all of them further to the north. They sniff around a lot. The yearling is leading west in that area and 755 and the pups follow. All are out of sight at 2022.

I go downhill at 2027 and leave the lot at 2030. 755 is weak.

I do not get any signals at Slough at 2136. I get good signals from 926 at the Picnic Area and Exclosure Fence, then they drop off.

I later hear that at 2145 the white female came in from the west and crossed the road to the east in the Alum Creek area. She then went toward the RS. The three black males tried to cross but turned back due to traffic.

August 5:

It is 44 when I leave at 0448.

I see a young snowshoe hare in the usual place west of Upper Baronett.

I get a good signal from 926 between the two lots, then lose her.

I do not get any signals at Slough when I arrive at 0535.

I look from the hill through 0603 and do not see any wolves. I go back to the lot and get a moderate signal from 969. I look from the entrance lot and do not see anything. I go west at 0616.

I turn around at the Yellowstone bridge at 0624 on hearing that wolves have been spotted at Slough. They are seeing 969 and two gray pup through the tops of the horizontal forest trees.

Deb calls to say she has been seeing some of the wolves in Hayden.

I see two gray pups above the horizontal forest at 0647. I go down the road and from the fourth lot see 969 bedded north of where the pups had been. She is looking south and howling. It is 0703.

I only get 969 when I go at 0728.

I call Deb and she says she has been hearing howling from the Alum lot. She had seen the adults and pups east of the western trees. A grizzly came into the area and the wolves ran into the trees. Later two of the black males and both females came out of the trees and played together as they went east. She thought she saw the third black male bedded near the trees. The four adults went northeast and then the fog came in. Three grizzlies had gone that way so there may be a carcass that way. Deb is seeing all four pups now.

I hear that the adults are back in the Hayden RS at 0746.

I get 755 as I approach the Mary Mountain Trail at 0827.

Deb is at Alum. She saw the white female north of the island of trees, then lost her going into the trees.

I go to Grizzly Overlook and hear that 45 to 60 minutes ago 755 came in from the north and went into the western trees.

I go up on the hill and see a gray pup bedded east of the western trees.

The plane is circling at 0849.

Lizzie talks to Erin on the radio and hears that she saw the three black males bedded in the western trees and got signals from 755 in that area.

I see the wolves come out of the trees and go southwest. They are west of the point of trees in the western forest. I saw the three black adults and the four pups. I lose them behind the island of trees.

I go down to the lot and head north at 0910.

I look from Alum and have all five adults and four pups bedded on the other side of the water, south of the island of trees. The three males and the white female are bedded on the mud.

I hear that a wolf is visible to the west of the Alum lots. I look that way and see 755 only 300 yards to the west. He is heading north parallel to the road. His coat is shaggy but looks good. I notice that he has an injured area on his right hip. That site is wet with blood and it looks like there are some puncture wounds there.

755 goes north at a steady slow pace and does not seem to be limping or in obvious discomfort. We lose him going up in the trees on the hill with the power line at 1025.

I look at the other wolves from Alum at 1050 and see that most are still bedded. Two gray pups go toward the western trees.

They are still bedded there when I leave at 1139. 755 is weak at that time. I lose the signal as I approach the Otter Creek Picnic Area.

I get 911 at Slough Creek at 1232 and 1256. I look, but do not see any wolves.

I get good signals from 926 and 965 in Soda Butte Valley and in Round Prairie, then lose them as I continue toward Ice Box Canyon.

I talked to Erin about her flight in the afternoon. She saw the three black Mollie’s males sleeping in the western trees and got a good signal from 755 that indicated he was 300 to 400 yards west of them in the trees.

She flew over Pelican Valley and got signals from 779 and 890. There were five black adults and two gray adults, along with three black pups. They were spread out and howling. Erin saw one collared black and from the air could not tell if it was 890 or 779. She felt there was some type of interaction going on but could not tell if he was with the other wolves or retreating from them. The pups were in the trees and the adults were in the open. 978 was one of the grays seen. Erin commented that the wolves were acting weird. I recall that Dan said 890 had DNA linking him to the Mollie’s. I last got his signal at Slough on 7/22.

Erin got 969 and 994 in the Slough den area.

She did not get any Prospect signals anywhere and did not see any wolves at Blacktail. There is some concern that 821's collar is not working. It’s last upload was on 7/27. Erin added that a collared gray with a Lotek collar was seen at the Otter Creek dump site by rangers and she wondered if it was 996.

She did not get 926 or 965.

The Eight Mile wolves were at Fan Creek in the northern Gardiner’s Hole area.

She got signals from Cougar female 953 in the Cougar Meadows area but did not see her.

I recall that 890's DNA indicated he had a connection with the Mollie’s wolves.

I wonder if he might have bred 779 when she was up in the north during the February mating season. We think that all the males in her pack are too closely related to bred her. If that is the case 890 would be there to visit her and their pups.

I called Abby and talked to her about the GPS collar on 965. It records two positions per day. In the early denning season he was based at the Druid den. She checked his locations for the last ten days and said that from 7/30 to 8/2 he was north of the Needle. On the 3^rd^ he was in lower Cache Creek. Today he was near the Fossil Forest area. Other recent locations included the Druid den area, the area southeast of Thunderer and Cache Mountain. That last area is close the eastern park border.

Her last sighting of 778 had him with a gray member of the Slip and Slide Pack. That was two months ago. There are seven adults in that pack and two of the ones other than 778 are collared. She thought that the pack probably did not den and that 778 is the likely alpha male. Three members of the pack were shot in the last wolf hunt, including the previous alpha male.

We talked about the four male yearlings that dispersed from the Lamar pack in the spring of 2015. We know that 967 and an uncollared black were found dead. That leaves another uncollared black and an uncollared gray. She said there is a pack in Cinnabar Basin that had pups last year. There are three gray adults she said one was unknown to her and possibly could be the missing gray male.

I later hear that the three black males, the gray yearling, the white female, and the four pups were seen at Hayden in the afternoon and evening. The white female crossed the road to the west just before 1500 and the others were in the RS. 755 was not seen.

At Slough a black and gray were seen in the evening downhill from the den area. People were unsure if they were pups or adults. I also hear that two collared grays were spotted in the lion meadow at 2030.

August 6:

It is 54 when I leave at 0453.

I do not get any signals in Lamar or at Slough at 0543.

I look from the hill at 0552.

We hear howling that seems to be to the west at 0606, but do not see the wolf.

At 0631 we see pups at the lower right side of the diagonal forest. I soon have three black pups, three gray pups, and the drab yearling.

I leave Slough at 0752 after doing a talk.

I later hear that all eight pups were seen after I left.

I get weak signals from 755 at the Chittenden bridge at 0844. He is loud at Otter Creek. I do not get him at Alum.

I later hear of carcasses placed at the dump up on the service road west of the bridge.

At 0856 Deb tells me that she has seen the four pups, but none of the adults.

I look from Grizzly and at 0920 see a gray pup east of the gully. I later have another gray pup and the black pup. They all go into the western trees.

I head north at 1140.

I get a good signal from 755 at the Otter Creek Picnic Area at 1145 and at Otter Creek. He drops off at the bridge.

I get 969 at Slough at 1247.

I do not get any signals in Lamar.

I hear that at Slough in the evening an adult and three black pups were seen for about a minute.

Two gray pups and the black pup were seen in Hayden in the evening. There was a report of what was said to be a dark gray pup west of the road at Alum in the evening with something in its mouth. It was trying to cross the road to the east. I wonder if it might have been a coyote.

August 7:

It is 42 when I leave at 0457.

There was a lot of rain and lightning and thunder during the night.

I do not get any signals in Lamar.

I get a weak omni signal from 969 at Slough at 0548, then lose her.

Carl calls to say he saw 763, a gray adult, and a black pup at Blacktail.

At 0625 I start seeing pups playing at the current RS at the lower right side of the diagonal forest. I soon have a black pup and two gray pups. We see 907 just north of them. She goes north and we lose her behind the horizontal forest. The pups play together and head that way. We lose them.

Deb calls to say she lost the five adults in Hayden going south.

I do not get any signals when I leave Slough at 0712.

I get weak signals from 755 to the north of the corrals, then lose him. It is 0803.

Deb has been seeing pups in the notch.

I look from Grizzly and at 0812 and see all four pups there. They run northwest into the trees. Then the four pups run out of the trees to the east and meet up with the white female. They harass her and she does a regurgitation to them. The gray yearling and three black males come in from the east. The pups chase the yearling and it looks like she did a regurgitation.

I do not see any of the males doing a regurgitation but may have missed it. The pups concentrated on the two females.

I lose the wolves going into the western trees at 0821.

I do not get any signals at 0826.

I hear that a cow bison died out from the Hitching Post hills yesterday. She had been dying there over the last seven days. I later hear that another bison died up near ranger rock on the west side of the Institute.

I hear that wolves are visible from Alum. I go there and see the two female to the northeast of Alum. The white female goes south and swims the river to the east side. The gray female follows. Both cross the road to the west and we see them off and on as they continue west.

We hear howling from what must be the black males from the trees to the northeast. I then see all three blacks in that area. They go back and forth and do a lot of howling. The blacks probably wanted to follow the females west across the road but turned back due to people and cars.

The collar on 1014 is crooked and swings back and forth as he walks.

I spot a grizzly to the west by the poles. It slowly comes west and crosses the road just south of our position. I ask people to go back to their cars that are on the hill. Rangers Bruce, Rebecca, and a bear tech are in the area and helping with crowds. The bear goes east toward the river.

The three black males are bedded between the two islands of trees and continue to do a lot of howling. They casually watch the bear to the south of them. I see two gray pups north of the males.

The bear tech tells me that there is a carcass to the west but out of sight. He also says that on July 29 and 30 a collared gray was feeding at the carcass dump. When he sent a photo to Doug Smith, he said it looked like 978. We also had information from Erin that Prospect wolf 996 might have been there. The bear tech also tells me that he saw 755 at the carcass dump on July 26. A black was there on the 30^th^.

I go north from Alum at 1115 and do not see any wolves from Grizzly. I do not any signals there when I leave at 1133.

There is heavy rain with some hail at 1155. There is also some lightning.

I do not get 755 as I go north.

I do not get any signals at Slough at 1301 or in Lamar.

I walk out on the Hitching Post trail and at 1319 see the new bison carcass to the south. There has been some feeding on it.

I go east at 1416 and do not get any signals.

I later hear that in the evening a black was seen chasing bison in the Chalcedony RS. That was around 2000.

All nine of the Wapiti wolves were seen from Grizzly Overlook in the evening. The gray yearling came in from the west and regurgitated to the pups.

August 8:

It is 38 when I leave at 0513.

At 0540 I see Small T on the bison carcass out from Footbridge. I do not get signals from 926 or 965.

Small T was probably the black seen last evening.

She is still feeding when I go west at 0550 to check on the carcass at the Institute. When I get there, I see at least five coyotes at or near the carcass. I do not get any signals.’

I go back to Footbridge and at 0618 see Small T going east on the middle flats. I see a small blaze on her chest. Her tail is thin but fully furred and her coat looks good. She goes up th west side of DPH and we lose her there.

I do not get any signals at 0628. Two coyotes are now feeding at the carcass.

I hear that a car hit a bison two miles west of Pebble Creek two nights ago and the bison walked off.

I go to Slough and do not get any signals there at 0655. We do not see anything. This is the first day since 4/14 that we did not see any wolves at Slough. I leave at 0705 and head to Hayden.

Kevin Dooley rode up the Slough Creek Trail that morning and later told Lizzie that he saw three to four pups in the first meadow along with a black adult and two gray adults. The wolves did some howling.

At 0807 I get a good omni signal from 755 just north of Canyon Junction by the Cascade Lake Trail.

I go on and see that Hayden is fogged in. I stop at Grizzly and at 0832 hear howling to the northeast, toward the RS. It is too foggy to see. I do not get any signals from 755 or 890.

Carl spots the wolves from the meadow north of Grizzly. I go there and at 0852 see the five adults bedded to the east. The four pups are nearby and walking around or playing.

I go up on the hill west of Grizzly and look from there at 0930. 1015 does a LFU.

The black pup and two gray pups wade in the creek east of the adults. They soon move off to the west/northwest and head toward the western trees. They mouse on the way.

The adults are still bedded when I go downhill at 1000.

I still get 755 as I go by the Cascade Trailhead at 1024.

I go to Mammoth and fill Dan in on some things. I leave there at 1326.

I do not get any signals at Slough or in Lamar and do not see any wolves at the carcasses.

Kate and Ger went to Footbridge in the evening and saw Small T near the seedlings from 2000 to 2100. She walked around, did not go to the carcass, and went back into the seedlings.

Fred was in Hayden in the evening and saw eight wolves in the RS, all but the yearling. At 2030 they all went south and were south of Grizzly Overlook when he left.

August 9:

It is 43 when I leave at 0509.

I do not get any signals at Footbridge.

At 0544 I see a grizzly feeding on the carcass. It leaves and at 0555 Small T comes right into the carcass. She feeds for a few minutes. At 0607 she looks west and leaves the site and goes up the bank. I wonder if she saw people going on the Hitching Post trail and that caused her to leave. I later hear that a group did go out on that trail.

The bear is going southwest toward the river and the wolf heads toward the seedlings. She looks back a lot. I lose her south of the seedlings, going southeast, at 0609.

Lizzie arrives and says she did not get any signals at Slough.

I do not get any signals here at 0611. I go west at 0621.

I only have ravens at the Institute carcass at 0638 and do not get any signals.

Doug says he has seen six wolves at Blacktail tail: three blacks and three grays.

I join him at Nature Trail and at 0718 see the bedded wolves in the western RS. I have two blacks and two grays. Then we see a bedded gray pup apart from them. I only get signals from 996. I heard that 821's collar is out.

A collard gray gets up and does a SU. That should be 821. The gray pup goes to the main group and all the wolves get up, move off a bit and bed again. The other gray looked uncollared and had a limp. Both blacks looked like adults and Doug said one was collared.

They are still there when I leave at 0815.

I hear from Kerri that she briefly saw eight wolves from Alum. She lost them going south.

At 0856 I get a good signal from 755 just as I start going downhill from Dunraven Pass.

I go up on the hill west of Grizzly and at 0927 see the five adults and four pups to the east, near the edge of the forest. They are going south with the white female leading. The pups are following, and this is the furthest south I have seen them.

I hear that three grizzlies have been see a mile or so to the south at a likely new carcass site. That site is in a gully and the possible carcass is not in sight.

The nine continue south. The uncollared black does a RLU. 1014 sniffs the site and does not mark it.

I go south at 0945 and see the wolves continuing on as I drive. I stop further to the south and 0958 and see that the white female is still leading. She is just east of the river by the small drainage where the bears had been seen. I see her looking around. I wonder if one or more bears are at the carcass and preventing the wolves from getting to it.

I hear a lot of howling to the east of her and see the three black male retreating to the east. There is a big crowd and that must be making them move away.

I also see the gray yearling near the drainage.

The males move uphill to the edge of the forest. They bed and do a lot of howling.

I do not see any pups and hear that they stopped and turned back before reaching this area.

I lose the males going uphill into some trees.

I see a few ravens flying in and out of the lower part of the drainage. I have lost the white female in that area.

The gray female is there. She moves south along the top of the drainage and does a lot of sniffing and looking around.

I still hear howling from the males at 1035.

Two of the blacks have come downhill a bit toward the gray female. They howl. I hear that the third black is south of them. I hear howls from that area.

The two visible blacks are looking around like they are trying to see that howler.

The cluster crew has been with me. They walked to an uphill spot north of me and say that they can see the white female and a grizzly at a carcass near the outlet of that creek. The bank blocks my view.

The gray yearling joins the two blacks and does a fast tail wagging greeting of both. The other black joins them.

At 1103 the crew tells me that the gray yearling has joined the white female at the carcass site.

I walk up to the crew and join them at 1126. I see the bear bedded at the presumed carcass site and both females are bedded near it. The grizzly gets up and moves toward the white

female. She gets up and walks off a bit. When the bear stops, she stops.

The bear goes back to the carcass and occasionally takes a few steps toward the nearby bedded white female. Each time she casually gets up, moves off a bit, then beds when the bear goes back to the site. The yearling is bedded further away.

I hear that a black pup and a gray pup are in the RS and heading toward the western trees. Later another gray pup goes that way.

I see the white female eating a good sized piece of meat just east of the bear. Later she moves off and it looks like she is pushing dirt over a cache with her nose.

The yearling is feeding on meat where the white one had been eating.

The white wolf carries off another piece of meat, digs out a cache, drops the meat in it, and pushes dirt over the site.

She had a long dark streak on the back of her tail.

They are both still there when I leave and head north.

I do not get any signals at Slough at 1351 or in Lamar.

Lizzie and others went up to the first meadow and saw all eight pups and seven of the ten adults. 890, 911, and a gray female yearling were missing. She did not get 994's signal, but saw him.

Matt send out a message about 890 being in Pelican for about two weeks and today having a point just north of the Fishing Bridge area.

Kate and Ger saw the Wapiti pups in the RS in the evening. Deb later tells me that in evening all five adults ran down into the gully with the carcass and drove off two grizzlies.

August 10:

It is 43 when I leave at 0509.

I do not get any signals in Lamar.

I do a check at Slough at 0607 and do not get any signals.

I get a weak signal from 755 as I start downhill from Dunraven Pass at 0654. It gets better as I go further down slope.

It is foggy as I go by Grizzly Overlook at 0712.

I join Doug at the lot near the carcass to the south and at 0716 see the uncollared black to the east.

I go up on the slope and at 0742 see the white female and gray female feeding on the carcass. No bears are there. I now see that this is a cow bison.

I see two of the black males howling uphill from the carcass area. The third black is near them.

I have been getting a good signal from 1014. We had thought that his collar was dead. I call Doug, Dan, and Matt and let them know.

Dan later texts me that 1014 still had not uploaded since 7/19. He checked 890's most recent point from this morning and found that he is still in Pelican Valley about where Erin had him last Friday.

Deb tells me that 1014 and 1015 were in the gully and feeding earlier in the morning. Both males left when the crowd got bigger. She also tells me that the uncollared black ran in to the site, grabbed some meat, an carried it uphill.

The white female leaves the site and goes north at 0800. She soon stops and looks east and uphill at the three blacks. They go downhill toward her. The uncollared black does a RLU. 1015 also does one. She moves behind a lot hill and the male likely meet up with her there. The gray female is still at the carcass site.

I go downhill and drive back to Grizzly at 0813.

At 0824 the four pups are walking around the RS.

I see the white female to the east of the lot. She is leading toward the RS and the pups. The three males follow her, and the gray female is behind them.

The black pup is walking around with a dead sage branch. It must have dropped it for now a gray pup is carrying it around.

The adults are crossing the mud flats just south of the pups. At times the pups seem to look that way but must not be seeing them.

The adults stop and sniff around the mud flats, then continue north. The white female gets up on the bank on the north side of the flats and stops to look at the pups. It is 0831. Two of the pups are looking at her. After a few moments of looking they wag their tails and run to her. The other two pups do the same thing. They all romp toward her.

The gray female is with the white female and the males are behind them. The pups run to the females and try to get regurgitations. The two female run off and the pups chase them while still trying to lick their faces. The males arrive and I see a gray pup greet one of them in the face. The pups do not try to get a feeding from the males anywhere near as much as from the females. I see the white female snap at a pup. We do not see any regurgitations from the adults. By 0834 the pups are losing interest in getting a feeding.

The gray yearling is playing with a gray pup. The uncollared black comes over and sniffs that pup as it squirms on the ground under him.

Then that black adult plays with the gray yearling as they roll around on the ground.

A gray pup greets 1015 in the face and he gives the pups a slight holding bite.

Then 1015 rolls on the ground with three squirming pups. There may be a scent there he is interested in.

The uncollared black gives a subordinate greeting to 1014.

Now the gray yearling and the three black males are all playing together.

A gray pup does a SU.

At 0842 the white female leads east toward the point of trees. She crosses the creek and beds. The black males go there. Some of the pups follow them. I lose all of them in that area.

A gray pup is still out in the meadow.

I do not get any signals at 0950.

I go to Alum and at 1015 see one pup behind the island of trees.

I go north at 1022.

Doug saw two blacks at Blacktail.

I do not get 755 as I go north.

I get a weak omni signal from 969 in the upper Antelope Creek area at 1044.

I do not get any signals at Footbridge at 1139.

I talked with Matt about 890 and he sent me a map of his point. On 7/24 he started to head southwest toward Pelican Valley from upper Lamar River. Some days did not record during this period. He was in the main Pelican Valley area from 7/29 through today but probably was there prior to that, but those points did not resister. On August 2 and 3 he mad. a trip northeast toward the Lamar River. He was near Lake Yellowstone on 8/6 and north of Fishing Bridge on 8/9.

Lizzie and Bill saw 17 Junction wolves in the first meadow. 890 was the only missing one. She saw 994 but did not get his signals. Since she had 969 there that means that I was getting her signal from a long ways off, but up high.

I later hear from Carl that in the evening all the adults in Hayden went west at 2130. The two females crossed the road. The uncollared black and 1015 also crossed. There was a lot of howling. All four adults went west. 1014 did not cross. He went back east to the pups. At least three pups were seen.

August 11:

It is 37 when I leave at 0517.

I do not get any signals in Lamar and do not see anything at the two carcasses.

I stop at Slough and do not get any signals at 0609 and do not see any wolves.

I get a weak signal from 755 as I go downhill from Dunraven. He is better in the Cascade Lake trailhead area at 0700

As I approach Alum Carl says that he has the four pups to the east. The white female just crossed the road to the west. She is probably going to the bison carcass in that area that was discovered on 8/7. The trail in that area has been closed.

At 0710 I see the white female to the west of Alum. She is going west, toward the forest. The four pups are visible to the east near the island of trees across the water. A gray pup is chewing on a plastic bottle. The black pup is being pinned by the other two gray pups and they are nipping at it.

At 0722 I go to Grizzly Overlook. I do not get any signals there.

We see the pups out in the RS from the hill.

Around 0845 we see the five adults coming back from the west on the north side of Alum Creek. The white female is leading east at a fast pace. The gray yearling is behind her and the three black males are further behind.

I go back to Alum and at 0857 see the two female about to cross the shallow creek to the west. Both look full. We lose sight of them to the south, then hear that they both crossed the road to the east south of us, probably through the gully that the wolves often travel through.

The three males have turned back. They go all the way back to the forest to the west and go out of sight there.

I look east and see the black pup howling. Then it runs south and I see the two females there. All the pups run to them and greet the females. They try to get a regurgitation, but we do not see any feedings.

I go to Grizzly Overlook and see the wolves going toward the point of trees to the east. The gray yearling wades the creek and goes toward the cut bank. The white female is there.

They end up south of the point of trees. Most of them go into the trees in that area at 1006.

After doing a talk I do not get any signals at 1048. I go north.

I heard that at times there are signs at Norris Geyser Basin that say: Lot Full Come Back in an Hour.

I get a weak signal from 755 as I approach Canyon Junction at 1056. He is better at the trailhead lot.

I go to Gardiner and head back through the North Entrance at 1644.

At 1721 I see a black adult in the western RS at Blacktail.

I get a weak signal from 755 at Canyon Junction.

It is raining hard.

I go to Grizzly Overlook and at 1836 see the gray yearling and the three gray pups near the eastern point of trees. They do a lot of playing together. The white female and the black pup are bedded away from them to the west.

At 1900 the white female gets up and goes west. I later hear that she crossed the road to the west and probably is going back to the bison carcass that way.

I head north at 2026 after doing a talk.

I later hear that the three Lamar wolves were seen near the cone in the evening. The two females had good coats, but 965 still had some mange.

August 12:

It is 38 when I leave at 0520.

I do not get any signals in Lamar and do not have anything at the two carcasses.

I also do not have anything at Slough.

I get weak signals from 755 as I start downhill from Dunraven at 0655. I also get him on just south of the corrals at 0704. He is best at the Brink Road.

There is fog as I approach Alum at 0708.

I go to Grizzly and see the four pups in the RS after the fog lifts.

Quinn is flying and he calls down to say 755 is heading toward the RS from the north.

We later see him coming of the notch in the western trees. He goes east, toward where the four pups have been walking around and mousing. 755 looks around a lot and has not yet seen the pups. He is mainly looking east toward the point of trees and the pups are more to the southeast of him

755 sniffs around as he goes. He turns back and sniffs around the area just east of the western trees. After that he goes east again and continues to look around.

The pups are now in the mud flats southeast of him. They are slowing going west as 755 goes east north of them. He continues to look around a lot.

One pup runs around with a bison pie in its mouth. It is 0933.

At 0936 755 stops and sees the pups to the south. His head is high as he watches them. At 0939 he takes a few steps toward them, then stops and stares at them for some time. He must be making sure that none of the black males are around. I see him stare at the pups and look around the general area.

He goes forward at a fast trot at 0944 and is in the mud flats north of the pups at 0947. The pups are going west. 755 now has a lot head as he watches them and stalks toward the pups.

A gray pup sees him and runs to 755. He stops and looks at the pup. Now all four pups are running to him. It is 0954. He just stand there. The pups reach hm and greet him in the face. He runs off to the west at a slow pace the pups chase him. They try to get a regurgitation, but we do not see one.

We lose them behind trees further to the west at 0958.

I go north to Alum and see some of them at 1015. A black pup is howling. Other pups are running back and forth. I spot 755 south of the pups and he is watching them. I see them off and on. They are slowly moving north. A gray pup goes to 755 and greets him. I see two pups running north. 755 follows them. I see all four pups north of him. He stops to watch them. I then see him sniff a gray pup. We lose them going further north.

Then we see the other five adults to the east, coming out of the point of trees. It is 1059. They go to the western trees and all four pups come out and run to them. The pups converge on the white female and greet her. She runs off and the pups chase her and try for a feeding.

We do not see 755 and the three black males do not seem interested in looking for him.

The wolves go north, and we lose them in the western trees. The white female was leading.

I go to Grizzly and look for them at 1122, but do not see anything. I get a weak signal from 755.

I go north and see 755 swimming the river to the west north of Alum. He comes out and crosses the road to the west at 1150.

I look at him and see a ding in his fur on the right side of the base of his tail and what may be a few puncture marks on the right hip.

We lose him, then see him much further to the west at 1204. He goes over a hill north of Alum.

I go north at 1218 and do not get any signals from 755 or 1014.

I get weak signals from 926 at Dorothy’s and at the Institute at 1331. I lose her as I continue east.

Lizzie and Doug went up to the Slough first meadow and aw 16 Junction wolves. 890 and one of the gray female yearlings were not there.

Quinn flew this morning. He did not get signals from 1014. 890 was at Raven Creek and going back toward Pelican Valley. Quinn saw 779, 978, four black adults, and four black pups there. He got 926 and 965 near the base of Bison Peak, up from Rose Creek, but did not see them. Quinn got 994 when he flew over the Junction wolves.

He only got 996 at the Blacktail RS and did not see any wolves there. There were five black Eight Mile adults and two gray adults at Gardiner’s Hole and did not see any pups. 962 and 1005 were elsewhere.

The three Lamar wolves were seen north of Mid-Point at 2000 and went out of sight heading toward the den area.

I later hear that just the four pups were seen at Hayden in the evening.

August 13:

It is 0522 when I leave at 522.

I get a weak signal from 926 at 21's crossing at 0549. I go on to the Institute to check if the wolves might be on the carcass there. I do not get anything there and do not see anything at the carcass.

I go back to the east and get a good signal from 926 in the Hitching Post area at 0605. She seems to be out toward the creek. We look, but do not see her. Later the signal seems to be better to the north, toward the den forest.

I go west at 0724.

I hear that the four pups have been seen at Hayden.

I get to Alum Creek at 0830 and see the black pup and two gray pups in the mud flats to the east. 1014 and the third gray pup are further to the east at the point of trees. 1014 is howling.

I get some weak signals from him.

I hear that 1014 came out of the eastern trees, went north, and greeted that pup.

We lose sight of him and that pup. I later hear that they went back into the trees.

I go to Grizzly and see the black pup and a gray pup on the west side of the RS meadow. The black howls.

I go up on the hill and look at 0917. I hear that the black pup and gray pup went into the western trees. A gray pup is still visible.

I then see the black pup and other gray pup.

I do not get 1014 now.

I do a talk for two groups on the hill.

I go north from Grizzly at 1146.

At 1329 926 and 965 are both weak in the den area. She is better to the north. I head in at 1351.

It is 75 when I leave at 1819.

I get weak to moderate signals from 926 and 965 in the den area. I had started to get 926 at Soda Butte East. I go west and still get a weak signal from her at the Institute. She is probably up high.

I have not seen coyotes on the bull bison carcass since the first day and have only see a few ravens there off and on. Often there are no ravens. Fur has been plucked from the bull’s shoulders and neck, but the carcass is intact from the road angle. The coyotes and birds had been feeding at the rear end earlier.

I go back to the den area and continue to get the two signals. I head in at 2012.

We did not know it at the time, but lighting stuck a tree in the Slough Creek area today and started what became the Buffalo Fire. It would burn through the Slough den area and end up being 10,800 acres by early September.

August 14:

It is 45 when I leave at 0517.

I get a weak signal from 926 at Footbridge at 0547. I continue on and she gets better as I approach the Exclosure Fence.

I see an east bound car stopped ahead of me. I spot what must be Small T approaching the road from the south at 0551. She goes behind that car. Then I see 926 crossing the road behind the car. Small T follows. I lose them going north. It is still somewhat dark.

I look from Trash Can and do not see them.

The signal has dropped off by 0605.

I go to Hitching Post and at 0610 get a loud omni signal from 926. I look at the Ledge Trail, but do not see them.

I did not get 965 at first there, but later get him

I still do not have anything at 0622 so I head west.

I hear that at 0705 the Wapiti gray yearling crossed the road and went west. The pups have been seen in the RS.

I stop at Alum Creek at 0729 and see the four pups to the east. They are mostly mousing. A gray pup jumps up and lunges at a marsh hawk flying over it.

The black pup carries around a plastic water bottle, then beds and chews on it.

I do not get any signals.

I go to Grizzly at 0901 and go up on the hill. We see one or two of the gray pups walking around in the RS from there.

I go back down to the lot and at 1040 still have one gray pup in the RS.

I go north at 1050 and do not get 755 or 1014.

At 1138 I get a weak omni signal from 969 at Upper Antelope Creek.

I do not get any signals at Slough. Emile had walked up to the first meadow and did not see any wolves but did hear howling from up creek and to the west. He was concerned about a man who seemed to be walking toward the sound of the howling.

August 15:

It is 41 when I leave at 0523.

I get variable signals from 969 from Hitching Post through Coyote. At Coyote I get her 360.

I go down to Hayden and at 0721 see one gray pup to the east from Alum. I hear that only pups have been seen so far.

I do not get 755 or 1014.

I go to Grizzly and at 0726 see all the pups mousing out in the RS. A gray pup makes several short runs, puts it head down, then lifts its head and seems to be chewing on something.

Later a gray pup does some digging at a spot.

At 0812 we see the five adults coming in from the west. I had just heard howling from that direction. The white female is leading, and the gray female is behind her. They both cross the road at the gully. 1015 was behind them. He turns back not far from the gully and goes back to the others to the west. Two cars had stopped on the road near that gully where the females had crossed.

The uncollared black is west of 1015 and 1014 has stayed back further to the west. 1015 is going toward them.

I recall that the pups had all looked west just prior to our spotted the five adults so they were probably hearing the howling from the adults several miles away. They did not howl back at the adults.

I look back at the RS and see the two females come in from the west at 0824. They are on the south side of the mud flats. I see a gray pup north of the mud flats.

The two females had been going east. They turn and run north, toward that pup. Both of them run through the shallow creek there. The white female stops and looks around.

The gray pup sees the two females, but hesitates a bit, then runs to the mother wolf. The other three pups appear and run to her as well. They chase her to the east and all of them go behind a tree. I can see them interacting there. She comes out a bit to the east and I see her do a regurgitation for the pups. The pups feed there. Soon they all run to the gray yearling and get a feeding from her. Pups run back and forth to the two females for more feedings. Soon some pups give up and feed at the previous regurgitation sites. It is now 0830.

Carl is at Alum and says that the white female did three regurgitations and the yearling did two.

A few ravens have landed at the regurgitation sites and the yearling chases them off.

The adults and pups all stop and intently look west, then howl. Fred tells me that one of the black adults to the west had just howled. The six wolves bunch up and have a rally. It is 0857.

I go north from Grizzly at 0910.

From the Alum lot I see the three blacks to the west. 1015 is going at a half run toward the other two males. I also see the two females and four pups to the east. The pups look like they might be on the west side of the river, meaning that they would have had to cross the water to get there.

I do not get any signals at 0917.

The pup are running back to the east and may now be on the east side of the river.

We see the white female running toward the road. She crosses to the west north of us at 0929.

I see the three blacks to the west. The female is heading toward them. At 0936 she reaches two of them: a collared black and the uncollared one. They have a long greeting, then run west toward the other collared black with the white female out in front. They have another long greeting.

At 0942 I see the pups to the east.

The four adults are going west, toward the forest. I turn around to look at the pups, then do not see the adults when I look west again. I hear that they went behind the cut bank going north and were lost.

A gray pup howls at 0947. I see all four pups and the gray yearling. All of them are looking west.

A bit later I see the gray yearling going west on the slope to the southeast. She crosses the road near where she and the white female had crossed to the east earlier through a gully in that area. The yearling recrossed well south of where the white female had.

The gray female goes north after crossing. She finds the scent trail of the white female and turns west to follow it.

I later hear that people saw a gray pup follow the yearling to the road crossing site, but it turned back. The pup had to have crossed the river to get there. The crossing would have been around 1019.

I see at least two other pups to the east across the river.

I do not get 755 as I go north at 1055.

I later hear from Deb that she has the gray pup between the road and the river. She says it is the big gray pup. Later she sees it swim the river back to the east. That was at 1130.

I do not get any signals at Slough at 1203 or in Lamar.

I now have seen wolves for eight months in a row or 244 days. That is since 12/16/15.

I later hear that in the evening in Hayden the four pups and the gray yearling were in the mud flats. They went out of sight to the west. A man walked out to the east from the Alum lot and scared the wolves. They ran off to the east.

August 16:

It is 42 when I leave at 0519.

There is smoke in Lamar and more at Slough. A new fire up on Buffalo Plateau is uphill from Aspen drainage.

I do not get any signals through Slough. At 0618 969 is faint at the west end of Little America.

I get to Alum at 0712 and hear that so far no wolves have been seen. I do not get 755 or 1014. I first look west and do not see anything.

Doug goes up on the hill west of Grizzly and spots wolves west of the three conifers. I join him and at 0726 see wolves by the conifer clump there. I soon have all five adults and the four pups. Two of the black males walk off to the east and bed in the mud flats. The others stay west of them and later go east.

I saw the big gray pup do a LFU.

The uncollared black leads east toward the eastern forest and the others follow. At one point I see the gray yearling doing a FLU. One of the collared blacks does a RLU there. The white female is east of them and she does a FLU there. The gray yearling sniffs that site and marks it.

The adults continue east and I lose track of the pups. At 1010 the white female beds by the creek, just west of the forest and the other four adults follow and bed near her. On the way the uncollared black and 1014 both did LFUs. 1015 is still limping.

At 1023 the white female is the only wolf visible. She is bedded on the creek bank and is howling.

I go downhill at 1024 and do not get any signals in the lot or as I head north.

There are several columns of smoke above the Aspen drainage from the Buffalo fire.

I do not get any signals at Slough or through Footbridge at 1204.

I later talk to Lizzie and she saw five pups, 907, and a gray yearling up at the first Slough Meadow.

In the evening Barb had the nine Wapiti wolves go into the trees to the east of Grizzly Overlook.

August 17:

It is 44 when I leave at 0458.

I get weak omni signals from 969 in Lamar. I do not get her at Slough.

I go through Tower Junction at 0546 and pass through Canyon Junction at 0616.

I see a big bull elk who has shed his velvet as I go south.

I do not get any signals at Alum at 0628 or at Grizzly. No one has seen wolves yet.

I go up on the hill west of Grizzly and look at 0646. We hear what may be group howling to the west at 0656.

I go downhill and drive south. At 0825 I see a gray pup in the RS from the lot south of Grizzly.

I return to Grizzly and see that pup and a black pup. I go up the hill. The black howls at 0902. At 0914 the other two gray pups come in from the west and go toward the first two pups.

We hear that the white female has crossed the road to the east through the gully north of us.

At 0933 we see her south of the mud flats. A gray pup runs to her and greets her in the mouth. She does a regurgitation to it. Both have their heads down there.

The black pup is east of them and she howls. The female goes east and soon all four pups are chasing her for a feeding. She looks back at them over she shoulder as she runs, then stops and does another regurgitation. The pups stop there and feed, then chase her again. She runs off and does a third regurgitation.

That third one may not have had much meat for the pups quickly run back to the first two sites and feed there. Then two pups try for another regurgitation and the rest of the pups feed at the earlier sites. It is 0940.

Later one of the gray pups does a SU. Another gray pup and the black pup go over and sniff the site, then walk off.

The five wolves are on the south side of the small creek. The wade to the north side and bed there at 1111.

I go north at 1145.

I do not get any signals in the north through Footbridge at 1318.

I talk to Quinn in the afternoon about 890's recent points. On 8/11 he was at Little Saddle Mountain, east of upper Lamar. The points were at Raven Creek, in upper Pelican on the next two days. On the 14^th^ he was at the Pelican RS. The next day he was in lower Pelican, not far from Fishing Bridge. The point did not register on the 16^th^. On the 17^th^ he was at the northeast end of Pelican Valley, two miles east of the den.

We do not know of anyone who walked up to the Slough first meadow to look for the Junction wolves.

In the evening Barb had two grays and a black bedded in the Hayden RS and thought that they were pups. I later get a report from another man who said as it was getting dark, he had six wolves approach the road from the east. There were five grays and one black. It must have been the two adult females and four pups. The females crossed the road to the west, but the pups turned back. The females crossed back and forth a few times to check on the pups.

August 18:

It is 46 when I leave at 0521.

I get weak omni signals from 969 in Lamar.

I join others at Grizzly Overlook and see a black pup and a gray pup bedded along the creek.

I get a text from Lizzie that she has seen one gray pup and one black pup at the first Slough meadow. Later Jeremy tells Doug that they saw six or seven wolves.

I go up on the hill and hear howls to the southeast. At 0740 I see a collared black howling from that area. He is near the old bison carcass there.

The gray pup is looking south, but does not seem to act like it is hearing the howls.

I soon have all three black adults in that area at 0800. They do a lot of howling. At 0805 the pups howl and the adults howl back. We then see the two adult females near the three blacks. They go north. 1014 does a RLU and the white female does a FLU at his site.

I now have all four pups together in the RS.

The white female is leading north. The others follow single file. They pass east of an elk calf in the river who is watching them. The wolves did not see it. They are going toward where the pups had been howling.

At 0829 they are crossing the mud flats.

Three of the pups are looking toward them. The fourth pup looks at the other three, sees the direction they are looking, and turns to look that way.

I wonder if that is why captive wolves do not understand the concept of humans pointing at an object. They might react to the direction the person is looking intently.

One pup starts to run south, and several others run with it, then they stop and hesitate. The black pup runs back to the north like she thinks there is danger. Then a gray pup runs south again the other two grays follow. They soon stop and look at the adults, then turn back like they are not sure of the situation.

The two females are wading the small creek as they are going toward the pups. They stop and look at the pups to the north. The pups look at them.

The white female continues toward them at a slow pace with a low head and in a stalk. The pups watch her, then all run toward the adults. They reach the gray female first and try for a feeding with her. She does not regurgitate so the pups run to the white female and three black males and try them. None of them do any regurgitations.

The black pup greets a collared black and he nips at the pup and gives it a holding bite.

A gray pup goes to the white female, sits up in front of her and licks her mouth and paws at her head.

A pup runs to one of the blacks and rolls on the ground under him.

The uncollared black nips at two gray pups rolling on the ground under him.

The white female beds and one of the collared black beds right after her.

The uncollared black lunges at a gray pup and gives it a holding bite.

All are now bedded.

I go downhill at 0918 and leave the lot at 0946.

I go Gardiner and later go east from Mammoth around 1530.

There is smoke from the Buffalo fire northwest of Mom’s Ridge, in upper Aspen Drainage and in Buffalo Fork.

I see two beavers in the river from the Lamar bridge.

I do not get any signals at Footbridge at 1709.

Barb saw eight of the Wapiti wolves bedded in the RS in the evening. One of the gray pups was not visible.

August 19:

It is 43 when I leave at 0518.

It rained last night.

I get a good signal from 969 at Slough. After looking at the usual areas I do a direction check and get her to the west.

Her signal is very good just past Aspen, then drops off.

Carl saw 763, a gray adult, and three pups near the Blacktail outhouse. They were on the road and went south.

I join others at the hill west of Grizzly and at 0748 see some of the pups bedded north of the creek. We soon have all four pups.

I walk off to look to the west and hear that two of the pups ran to the western trees and greeted the gray yearling. I come back at 0836 see her bedded with the pups.

A gray pup later goes to her and rolls on the ground under her, ends up on its back and paws at her face. We see her snarl and give the pup a holding bite. It is 0845.

I go south at 0954 to look for the other adults.

When I get back to Grizzly Overlook, I see a gray pup going into the western trees. I hear that others already went in there.

I go north after meeting up with Doug Smith and his family.

I go to Blacktail and get a weak signal from 996 at the S Curve at 1236. I look from there and from Nature Trail and do not see anything.

969 is weak at Boulder at 1220 and at Slough 1227.

The smoke from the fire is nearly gone.

August 20:

It is 31 when I leave at 0521. There is frost on the windshield.

I see that some campgrounds are now not filling up.

I spot a run over snowshoe hare in the usual area and it looks small.

I get weak omni signals from 926 from Soda Butte East through the confluence, then they drop off.

It gets down to 30 in Lamar.

I do not get any signals at Slough at 0606. There is almost no smoke left.

When I get to Tower, I hear from Doug that he has wolves bedded at Blacktail. I also hear that Hayden is fogged in and that it is 15 there.

I get weak omni signals from 969 at Petrified Tree lot and at Hellroaring.

I go to Nature Trail and at 0641 see wolves bedded on the hill at the western RS. I eventually see four blacks and five grays. The adults include 821, 964, and 996. There are two black pups and at least one gray pup. I see an uncollared black adult. That leaves two other grays in the group that I do not have a good view of.

Doug later tells me that he saw a second gray pup. We think that in total there are two black pups and two gray pups.

At 0802 I hear that the four pups are out in the RS at Hayden.

I go east from Nature Trail at 0841.

Calvin and Lynette went to Slough and saw an uncollared black at Aspen Pass. It has been doing a lot of howling.

I get weak signals from 969 and 994 in Little America.

I go to the first Slough lot and around 0920 see what looks like the gray male yearling going north from the pass. He drops down to the lion meadow and howls. The male goes up behind the lower right part of the diagonal forest at 0938 and later comes out of the gap at 0950. He sniffs around and looks around a lot as he travels south along the ridge. We lose him going through the pass southwest of the black stump.

Doug tells me that the Prospect wolves go up and went south where they met up with 763. They then went to the triangle forest and turned north. He lost them in a low area. Doug said that he had six grays and four blacks including 763.

Calvin earlier had the same count that I did: four blacks and five grays. We were up by Frog Rock and may have seen a gray that Doug did not from the lot.

Lynette got a call from Story in the first meadow and she reported that she was not seeing any wolves but did hear some howling. I talk to a man who hiked up there yesterday afternoon and on the afternoon of 8//17 and did not see any wolves.

I do not get any signals at 0956.

I head to Hayden at 1002.

I do not get any signals in Hayden at 1100.

We go up on the hill west of Grizzly Overlook and see a gray canine mousing north of the usual RS. It does not have a dark back so it might be a coyote. We do not see anything else.

I talk to Laurie about the lineup of adults in Prospect. There should be six black adults and four gray adults. For blacks there would be 763, 996, the spotlight black male, a black female, and two other blacks. The four grays would be 821, 964, 966 (the alpha male who lost his collar), and a gray female. With the two black pups and two gray pups there would be 14 in the pack with eight blacks and six grays.

I do not get any signals when I leave Grizzly at 1226.

I do not get any signals as I go north.

969 is weak at Slough.

We later hear that the black pup and two gray pups were seen in Hayden in the evening.

Kathie got a report about the Canyon alphas on a deer carcass south of Madison Junction this morning at 0700.

August 21:

It is 37 when I leave at 0525.

I get good signals from 969 and 994 at the Institute at 0604. We look for them, but do not see anything. The signals are dropping off at 0613. I do not get them as I go west.

The smoke has come back a bit to the north when I arrive at Slough. It is north of the den area and in the Buffalo Fork area.

969 and 994 are loud here at 0624.

I spot the black male yearling bedded in the lion meadow, north of the aspen clump.

We hear group howling from the diagonal forest area at 0625. The black does not lift his head or react to the howling.

After looking from the first lot I go down the road. I do not get any signals at 0656. I look from the fifth lot at 0700 and do not see anything. At 0703 there are faint howls to the west.

I go back to the first lot and see that the black is still bedded.

I do not have any signals when I leave Slough at 0720 to go west to look north from Little America. I stop at Long East and do not see anything and do not get any signals. I go back to the east at 0746 and do not get any signals as I pass Slough. I continue on to the Institute and do not get anything there at 0758.

I hear that people at Slough are seeing two gray pups going north through the yellow grass meadow. They go out of sight just as I get back to Slough at 0805. People tell me that there had been a lot of howling before the pups were spotted going north.

I hear that the yearling got up and went west. I see him going west, southwest of the Simpson Tree, then lose him. Then others see him going up the aspen drainage at 0838.

I leave Slough and head down to Hayden on hearing that the black pup is visible.

I get to Alum at 0937 and do not get 755 or 1014. I see the black pup and two gray pups to the east. They are walking around separately and mousing.

What looks like the big gray pup moves toward the black pup and she gives the gray a submissive greeting. The black rolls on the ground under the gray as he stands in a dominant posture and has a raised wagging tail. She also licks his face. This goes on for some time. The black then gets up and tries to climb on the back of the gray. The other gray pup comes in and also seems to act subordinate to the black.

They move off and head west, toward the western trees. I lose sight of them there.

I head to Grizzly Overlook at 1008 and go up on the hill. We do not see them from the hill and assume they went into the western trees.

Kathie tells me that the guard hairs come in at about 12 weeks on pups and that is what causes the black lines on their backs.

At 1047 the black pup comes out of the western trees and walks around mousing.

I go down and head north at 1131. I still do not get any signals.

I wonder if at this time of the summer the prey animals are much further away from den sites due to the summer heat and that caused the adults to be away from the pups much longer periods that earlier in the season.

I do not get any Junction signals in Little America or at Slough and do not get any Lamar signals as I continue east.

Story stayed in Hayden through 1500 and continued to see two gray pups and the black pup. The black went way to the south from the RS and was later east of the first lot south of Grizzly Overlook.

August 22:

It is 48 when I leave at 0530.

Due to darkness I did not look at the bison carcass at the Institute. I later hear that a grizzly sow with a cub of the year was seen at it at first light.

I do not get any signals at Slough at 0614.

I go west at 0629 on hearing that Doug has wolves at Blacktail.

I join him at Nature Trail at 0652 and see wolves bedded in the western RS. I eventually have five blacks and six grays. 821, 964, 996 are in the group. I also see two gray pups. 996 is limping.

Later the wolves get up and go southeast. Some bed and others continue on. Two end up east of the triangle forest.

I go east at 0922.

Lynette and Calvin are at Grizzly Overlook. They saw the four pups, both adult females, and 1014, but all of them are in the western trees now except for one gray pup.

I do not get any signals at Slough at 1025 and do not get anything in Lamar.

Lizzie went down to Hayden and did not see anything.

I later hear that seven wolves were seen at the Hayden RS in the evening. All four blacks were in the group.

August 23:

It is 34 when I leave at 0530.

I stop at the Institute and at 0605 and see a grizzly sow with an older cub at the carcass to the north. I do not get any signals.

I continue on and do not get any signals at Slough at 0631.

I go south from Tower and get a weak omni signal from 969 at lower Antelope Creek.

Doug calls to say he briefly saw 965 up at the Institute carcass. He also saw an uncollared black to the south by the river and thought that it was not Small T.

I later talk to Lizzie and she missed 965 but saw the other black. She got a good signal from 965.

I do not get any signals in Hayden. I do not see anything from Grizzly Overlook and hear that no wolves have been yet. There has been howling to the southeast of the last bison carcass to the south of Grizzly.

We see a tall man walking around in the closure area and I call ranger Kyle in to show him the situation. I hear that the guy was also seen out there last evening. I see him walking around and looking around. At times he sits in the brush and is out of sight. Other times he goes into the trees.

We hear howls to the southeast of the lot and at 0850 see 1014 there. He does a lot of howling as he looks south. Then he moves off to the north, but frequently stops to howl and look south. He has a limp when he continues north.

At 0929 we see the gray yearling howling in the flats to the northeast of us. She would be northwest of the male. The gray looks toward him.

Then she continues north and seems to be looking for the pups in the RS. I can see her vocalizing as she moves north and must be trying to call out to the pups to find them. She is looking around for them. At times she stops and howls as she looks around.

The gray goes out of sight into the western trees at 0928. Soon she comes out and goes east, still searching for the pups.

1014 is going back to the south at 0944. I wonder if the presence of the man last evening and this morning has caused most of the wolves to leave the RS area and to cause 1014 to go back to the south.

The yearling is now going back to the west. I lose her in the western trees at 0954.

I see her again at 1020. She is going back to the south through the RS. Soon she veers to the southeast and goes up on the rolling hills. I lose her heading into the forest near one of the sand pits at 1027. I hear that 1014 had gone out of sight in that area earlier.

I go south from Grizzly at 1114 and see the grizzly sleeping at the edge of the river east of the bison carcass in the river at LeHardy Rapids. A ranger tells me that this is the 11^th^ day for the carcass and that the bear just found it yesterday.

I get back to Grizzly at 1429 and do not see any wolves.

I stop at the Institute at 1759 and get a good signal from 965 and a weaker one from 926. I do not see them.

There is a new bison carcass to the south on Amethyst Bench. Three coyotes are southwest of it. A larger canine that looks like an uncollared gray is bedded between the carcass and those coyotes.

I do not get the two signals when I go leave at 1816. Then I get a weak signal from 926 as I drive off. I lose her right away.

I later hear that all three Lamar wolves were see at or near the Institute carcass around 2030.

I later talk to Kira about the flight that she had this morning. She did not get 755 or 890. The signals from 926 and 965 were up the east fork of Rose Creek and were not visible. 909, 963, three adults, and six pups were scattered in Gardiner’s Hole. She told me that the 24^th^ or 25^th^ 890's point was at White Lake in upper Pelican

August 24:

It is 36 when I leave at 0537.

I get 926 in the den area and at the confluence, but she is weak. Then I lose her as I continue west. I do not get her at the Institute.

The two grizzlies are at the Institute carcass at 0611.

At 0626 I get a loud omni signal from 926, but do not get 965.

The grizzlies leave the site at 0630.

I see a black wolf in the Chalcedony RS at 0636.

I drive to Trash Can and go up on the hill. I see the three Lamar wolves in the RS. 926 and Small T are playing together vigorously. They pause and Small T turns in circles as she tries to grab her tail. They get back together and playfully romp around. 965 is near them, but does not join in. From this distance his coat looks OK. The females continue their intense play.

926 has a lot of gray on her muzzle and chest.

They all go east to the Chalcedony fan and I lose them in the trees on the east side of the fan.

I go back to the Institute at 0710 and only see ravens on the two carcases.

It get down to 29 in Lamar.

I do not get any signals at Slough at 0734.

I hear that the wolves have been seen in Hayden, so I head there. I later talk to Bonnie. She had the wolves to the east of Grizzly Overlook. They went north and bedded in the flats. There is a new partly eaten carcass between the road and the river near the first lot south of Grizzly and thinks the wolves had been at that site. A grizzly swam the river from the east and fed on the carcass, then dragged part of the carcass back through the river to the east and is now there with that section. The wolves did a lot of howling when the bear came into the area. They apparently had already left the carcass site before anyone spotted them and before the grizzly came on the scene.

I do not get any signals in Hayden at 0826.

I go to Grizzly and see the bear bedded on the east bank of the river to the southeast.

I hear that the wolves are bedded in a low spot to the north/northeast of the lot. I do not see them.

I go up on the hill and at 0903 see some of them get up. I soon have all nine there.

At 0930 1015 gets up and looks at a mule deer doe to the southeast. He slowly moves toward her, then beds and watches her with a low head. The deer has not seen the wolves and she is now going north, somewhat toward 1015. The other wolves have not seen her.

The deer pauses to drink at small creek. She then slowly walks north. At that point she must have seen or gotten the scene of some of the wolves for she turns around and trots off to the south.

1015 now has his head up and is watching her. The white female gets up to look at the deer. 1015 also stands and stalks toward her. Now he goes after her at an all-out run. He gains on her. She is now stotting up the rolling hills to the southeast and is going toward the trees. 1015 slows down and soon stops. He sniffs at the scent trail of the deer and I wonder if he can tell that she is in good condition. It is 0939. Soon he is limping back to the others.

I see that the gray yearling had run part way toward 1015 during the chase. She is not stopped and looking that way. I see that she is full.

1014 does a RLU.

The four pups run to the gray yearling and get two regurgitations from her. They had been close to her earlier when they all were bedded.

I see the black pup do what looks like a LFU.

1015 howls. The uncollared black also howls.

Some of the adults seem to be heading toward the bear and the carcass. I hear that a lot of people are near the carcass in the lot south of us.

The white female stops and beds and the big bend I the river, looks toward the bear and the carcass and howls. The other wolves had stopped and bedded or are walking around northeast of her.

The bear is still bedded and may be feeding on the part of the carcass that it has.

All the wolves are bedded when I leave and go north at 1046.

I do not get any signals at Slough at 1215.

I get 926 and 965 at the Institute at 1233, then see both black females feeding on the southern carcass. Small T soon walks off to the southeast, pauses to look east, then goes into the trees. 926 soon leaves the site and also goes into those trees. I do not see 965 but get a good signal from him. I go east after doing a talk at 1322.

The people at the Institute did not see any wolves on the Amethyst carcass in the evening.

Nine wolves were in the Wapiti RS in the evening.

The office got an email today with trail camera photos of the Canyon Pack that were taken on 7/31. It shows the white female, a gray adult, and a gray pup at the Mesa Pit area. That is south of Madison Junction and in the Canyon pack’s area.

August 25:

This is the 100^th^ year anniversary of the founding of the National Park Service.

It is 34 when I leave at 0530.

There is some snow on the ground in Soda Butte Valley.

Fog is covering much of the valley, including the carcass site on Amethyst Bench.

I got variable signals from 926 from Footbridge to the Institute.

I later lose her at the Institute. She is good as I head east, but mostly 360.

It is still foggy, so I give up and go west at 0746.

I do not get any signals at Slough at 0755.

I join Calvin and Lynette at Nature Trail and at 0820 see five grays in the western RS. At least one is a pup.

I go back to the east at 0814.

I call Lizzie and she says she is not getting any Lamar signals.

I also hear that nothing is visible in Hayden.

I go south from Tower at 0922.

I get a weak signal from 969 at Antelope Creek.

At 0945 I hear that there are still no sightings in Hayden.

I hear that two blacks and a collared gray with an injured leg were seen at Slough between 0900 and 0930 so I turn around just south of Canyon Junction at 1007 and go back. Lizzie is at Grizzly Overlook and still has nothing in sight and no signals.

I get a moderate signal from 969 at Boulder.

I do not get any signals at Slough.

People at Slough saw two blacks just south of the eagle nest cliff at Slough. I briefly look for them there but have to leave at 1111 for an appointment at the clinic. Just after I leave people see one black bedded in that area and think that it is a pup. I later hear that a black pup and a black yearling were seen there. No one reported seeing the injured collared gray.

I leave Gardiner at 1331 and head out after stopping at the office at 1452.

I do not get any signals at Slough at 1621. People saw a black catch a small animal and carry it off just before I arrived. It was in the area just south of the cliff. At 1703 I see a black yearling bedded there and it is feeding on something.

I go east at 1708.

At 1730 I get loud signals from 926 and 965 toward the ledge trail from Hitching Post. The signals drop off at 1751 so I go west.

At 1755 I see the three Lamar wolves west of the west end of the ledge trail.

926 now has a perfect coat. She had a lot of gray on her chest and it extends all down her undersides to the area under her tail. There is also gray on the side of her face and on the outside of the upper part of her front left leg.

Small T also has a good looking coat. I see gray on the back of her head and on her muzzle. There is also a lot of gray on the back of her hind legs. There is a thin white line extending down from her small chest blaze. Her tail is full.

965 is almost completely recovered from mange. He face and torso are all normal. I see a fist sized dark area on his left hip and notice a thin black line down his right hind leg.

926 leads west. She and 965 bed together after she greets him at 1822 and Small T goes uphill and beds on the crest.

926 gets up and goes uphill. Her left hind leg is stiff. 965 follows. I lose all three over the crest at 1834.

I go to Mid-Point and see the three going further north. I soon lose them. I go east at 1845.

Deb tells me that at 1700 she saw a gray in the notch at the western trees and thought that it was 755. Earlier she had seen the black pup and black yearling at Slough. Later in the evening she had a grizzly on the Amethyst Bench carcass.

I later hear that two gray pups were seen in the Wapiti RS in the evening.

August 26:

It is 36 when I leave at 0534.

I get some faint signals from 965 at 21's crossing and at Picnic area.

969 is loud as I approach the Institute at 0606.

At 0611 I see her and a black yearling at the bison carcass on Amethyst Bench. 969 has a U shaped tucked tail and looks around a lot. She may be getting the scent there of the Lamar wolves.

This looks like the black female yearling. Her tail is thin, and she has a small blaze. At 0652 she leaves the site and goes southeast. I lose her in the trees.

At 0658 969 walks off to the west with a piece of meat in her mouth. I lose her in the trees at Amethyst Creek. She comes out at 0703 without the meat and probably cached it.

I do not get any other Junction signals or Lamar signals at that time.

I go east to the Picnic Area and do not get any signals there at 0711.

Carl saw two wolves crossing the road at S Curve and go east.

I do not get any signals at Slough at 0721.

Doug saw a black and two grays at Blacktail.

I go south from Tower at 0754.

It rains a bit as I approach Canyon Junction at 0828 and the road is wet.

I do not get any signals at Grizzly at 0846 and no wolves have been seen by people in the lot. I look from the hill through 1005, then go south to the three sign lot to the south. I turn around there at 1012.

Nell calls to say she is with people that saw a collared silverish wolf that was probably 755 across the river from the bison carcass at LeHardy Rapids last evening around 1930. Deb later sees a photo of the wolf and I see it as well. It is 755. The man who took it said a black was with 755.

I go to Slough and get variable signals from 969. She may be toward the diagonal forest.

People tell me that from 1115 to 1120 they saw a collared gray cross the road to the west near the third lot. It had what they described as a hole on her upper right shoulder and was limping badly. They show me four photos of the wolf and it looks like it could be 907. The wolf is thin, and I see what could be the GPS element on the top of the collar. There is not a good view of the bottom of the collar. I later revised my thinking and felt that it was 911. See later observations of 911.

I only get 969 at 1201.

I do not get any signals in Lamar.

I head out again at 1734.

I do not get any signals in Lamar.

I see a black yearling feeding on the bison carcass at 1806. She goes off to the west at 1940. I later hear that Kathie and Jeremy feel that the black yearling this morning and evening was Big T from Lamar. They felt it did not match either Junction black yearling or Small T.

I do a talk for about 40 students, mostly African Americans after showing them the black wolf.

August 27:

It is 34 when I leave at 0535.

I have back from the heart operation now for a full year now. I went out every day starting on 8/27/15 and saw wolves on all but four days through now.

I only get 969 in Lamar.

At 0609 I see her, the drab gray female yearling, and a grizzly on the Amethyst Bench carcass. The bear leaves at 0613. At times 969 has a tucked tail.

As I try to get out on the phone, I hear that 969 walked off the west with part of the hide.

Doug has seen four wolves at Blacktail: a black adult, a gray adult, a black pup and a gray pup. It is 0648.

I go west at that time.

I hear that the two Junction wolves went out of sight to the west from the carcass at 0700.

I get faint signals from 994 at Slough at 0706.

I head toward Blacktail.

I hear that 969 is going back to the carcass.

Around 0850 we see wolves just west of the triangular forest. They go east and I have a black adult, a gray adult, and a gray pup. Then I see the limping 763 follow their route. All go out of sight into a gully.

I go east at 0951.

We get a report that people at the Slough trailhead heard howling to the west and saw a black and a gray above the campground area.

I do not get any signals at Slough at 1020 and do not see anything. I go east at 1047.

I get signals from 969 as I go by Coyote. I only have ravens at the carcass at 1100.

I do not get any other signals as I go east.

Kathie and Jeremy hiked up to the first meadow and saw three black pups and a gray pup.

There were no known sightings in Hayden today.

I later hear that the Canyon alphas were seen crossing the road to the west in the Fountain Paint Pots area at 1900.

August 28:

It is 37 when I leave at 0530.

I do not get any signals through the Institute.

I see a grizzly on the bison carcass to the south at 0609. It leaves at 0633 and a coyote comes in. I still do not get any signals through 0700.

I go to Slough and do not get anything there either. I take a quick look, then head to Blacktail at 0715 on hearing that Lynette has a black.

I get to Nature Trail at 0740 and at 0757 see a black adult bedded just west of the triangular forest. A gray pup comes through and greets that black. In the western RS I eventually see what looks like 763, a gray adult and a gray pup.

I go east at 0846. I have not gotten any Prospect signals

We hear that the three Lamar adults were seen across from Soda Butte Cone. That sighting was around 0900.

I do not get any Junction signals as I go east.

I only see birds on the Amethyst carcass at 0938 and do not get any signals.

At 0952 I get a good omni signal from 965 in Soda Butte Valley, but do not get 926.

965's signal is dropping off at 1010.

I go west and get weak signals from him at confluence. I get weak signals from 926 at Trash Can.

The shorter aspens are turning yellow at the exclosure fence.

Both signals are faint at the confluence. He is better in the cone area.

I head in.

I hear that nothing has been seen in Hayden through the afternoon.

August 29:

It is 39 when I leave at 0535.

I do not get any signals at the Institute and do not see anything at the carcass. I go west at 0616.

I get to Slough at 0635 and see that there is a ground fire in the den area.

I go west and hear that no wolves are visible in Hayden.

I join Doug and others at Nature Trail. At 0802 we see one gray east of the triangular forest. It moves off to the west.

I later go to Slough to look at the fire. I leave there at 1123 and take a short break at Tower Ranger Station.

Lizzie and I go to the office for a 1300 staff meeting.

When I am there Matt tells me that 890 has been in the Pelican area from 8/14 through the 21^st^.

We go east from Mammoth and meet up with four Italian wolf biologist at Nature Trail and Michelle Cowardin from Colorado Parks and Wildlife. Nothing is visible there.

I take them to Slough and do not get any signals there at 1813. There is smoke in the eastern trees, but no live trees there have burnt. The two dead aspens near the natal den have fallen and have burnt. All the rest that has burnt is sage and grass so far. It has burnt around the sage den. The fire went from the crescent rock to the gap in the diagonal forest. I also see smoke behind the diagonal forest.

We then go to the Institute, but we do not see anything and do not get any signals.

I go east and still do not get anything through Footbridge at 1931.

August 30:

It is 41 when I leave at 0537.

I get good signals from 926 from Footbridge through the Institute.

There is a grizzly on the southern carcass at 0610. It leaves the site at 0616. Five coyotes are in that area.

I go east and now get both 926 and 965. We spot them at the apex of the big fan to the south of Picnic Area at 0734. The two females are there and 965 is just west of them.

Emile texts about the Wapiti wolves chasing an elk calf into the river south of the RS.

Small T does a scent mark. 926 sniffs the site and does a FLU and GS.

965 still has the spot on his left hip.

The three come together and go west. Soon they are approaching the bison carcass on Amethyst Bench. They do a lot of looking around and sniffing around. 926 does a FLU and a lot of GS.

965 goes to the carcass and tears off a piece of meat from the rear end. The two females are looking around. The male gets another piece of meat.

926 does a lot of GS and looking around.

Emile calls to say the wolves are trying to kill the calf in the river. The alpha female, the gray yearling, and one of the black males are in the water. The other two males are west of the road and howling. They have not been able to cross the road. There is a lot of howling.

Both females leave the carcass and go back to the east. The male follows. They have not been at the site for long and may be concerned about the Junction wolves coming in. I see them looking back to the west a lot. I wonder if they are hearing howling from that direction.

926 still has a stiff left hind leg. Small T is leading east.

It is now 0822 and I have the impression that the wolves are concerned about something as they look back to the west continually.

They run down the east end of the bench and the male looks back.

Now the two females do play chasing. Small T started it. She dodges as 926 chases her and it looks like Small T is faster and more agile than her mother. The male is nearby, but not involved. At times when Small T outruns her, 926 just stops and watches, then resumes the chase when Small T runs back by her.

926 and 965 play together briefly. Then 926 sees Small T run in and chases her. She bumps into 965. He runs off and she chases him. They jump up on each other.

926 runs in circles and goes back and forth with no one chasing her.

They are going east at 0846. 965 looks back to the west once more.

Emile texts us that the wolves have killed the calf. It is 0852.

I go to Slough and get 969 and 994 at 0859. After looking I go west at 0909.

I go to the three sign lot south of Grizzly in Hayden and at 1007 see the gray yearling feeding on the calf carcass on the west bank of the river. I hear that the white female, the gray, and one of the black males killed the calf in the river and the carcass was then dragged to the west bank. The white female did most of the work in getting the calf and killing it.

I look to the east of the river and on a hill see the white female, 1015 and the four pups. The white female howls and the male and pups join in. It is 1010.

The yearling is still eating and not joining in.

I hear that earlier 1014 and the uncollared black were seen just west of the road in this area. That was around 0800.

The black pup sniffs under the belly of the big gray pup. Then the black and a small gray pup lick the face of the big pup.

Later two gray pups lick the face of 1015.

1015 howls at 1028.

At 1037 the yearling swims across the river to the others. When she arrives the four pups chase her and repeatedly try to get a regurgitation, but she does not feed them, despite having just eaten.

1015 continues to do a lot of howling. He is staring west like he is hearing howling from the other two males.

We see a grizzly to the southeast. It is moving north. The bear ends up well east of the wolves and continues north without seeming to be aware of the carcass.

The two females are interacting with 1015. He puts a front leg over the shoulders of the white female. The gray yearling crowds against his side.

1015 does not have a blaze.

Emil is in the lot to the south of us. He says he is hearing two wolves howling to the west of him and another wolf is howling south of them.

1015 howls at 1049. He must be hearing the howls to the west.

The white female casually looks at the bear to the northeast.

The black pup does a SU.

I see a photo of the three wolves on the calf in the water. 1015 has a bite on the head of the calf.

The yearling has a big tail scent mark and has black on either side of the base of her tail. The white female also has a big tail scent mark.

1015 gets up and howls as he looks in different directions.

At 1109 the white female heads down to the river and 1015 follows. She wades in the river, then swims toward the carcass on the far bank. Soon she comes out at the carcass and feeds.

1015 had waded a bit into the river, then went back to the east bank.

Lizzie tells me that the Lamar wolves went into the trees to the south of Trash Can.

I go south at 1133 to look for the two black males and 755. I turn around at LeHardy Rapids at 1141 without seeing anything or getting any signals.

When I get back at 1200 the yearling is feeding on the carcass. I see the other wolves going into the trees to the east of the river with the white female leading. The yearling soon swims the river to the east with a piece of meat in her mouth. She comes out of the water, goes behind a hill and later comes out without the meat.

The bear is coming back from the north on the east bank of the river and must be now getting the scent of the carcass. It is sniffing the air. The bear swims the river and rushes toward the carcass. It arrives there at 1205. The bear drags it into the water and feeds on it there.

Emile comes over and gives me more information about the earlier observations. He was coming back from the south. 1015 was east of the river and the calf was in the water. The white female was bedded west of the river. She went into the water and chased the calf back and forth for about 15 minutes. At times they were swimming and at other times had their feet on the ground under the water.

1015 and the yearling watched from the east and did not go into the river until the white female finally made contact with the calf. All three got bites in the calf and killed it.

During that time the other two males were southwest of the road and howling.

The four pups were at tree line to the east of the river. After the calf was killed the white female swan the river to the east, got the pups to come back to the east bank, then swam back to the carcass and ate about 15 to 20 minutes after the calf was killed. The other two adults had waited before eating as well.

I go north at 1217.

I hear that at 1248 the three adults and four pups are back at the Grizzly Overlook RS.

I get a weak signal from 969 at 1334.

The burnt area now has gone to the northern pass and extends to the north end of the lion meadow. The marsh has not burnt but the area east of it and the area south of the two dead trees has burned. The burn goes all around the sage den and up to the western trees and the eastern trees. The area downhill from the ledge has partly burned and all the area above it has burned. It extends to the bowl area uphill and north from there.

Some of the area around the natal den has not burned.

The fire is now 4,000 acres.

I go east and get good signals from 926 and 965 at Trash Can at 1413.

I later hear that in the evening the white female was seen going south from the RS. The four pups and 1015 stayed in the RS.

August 31:

It is 46 when I leave at 0534.

I do not get any signals at the Institute and do not see anything at the carcass at 0607. I go west at 0631.

I get a weak omni signal from 969 as I approach Slough, but do not get her there at 0639.

Doug says he has seen five wolves at Blacktail.

I get there at 0707 and hear that the wolves are out of sight in the western RS.

I see a black lump east of the RS and it later gets up and we see that it is a black adult. It is now 0755. The black goes to the RS and acts like it is trying to find the other wolves. It looks around, walks back and forth and sniffs around.

I go west at 0924.

I hear that no wolves have been seen at Hayden. Smoke has made viewing difficult.

I get a weak omni signal from 962 at Swan Lake at 0956. He is better on direction to the west/northwest. I go north at 1023 and head to Livingston and Bozeman for doctor’s appointments.

I pass through Gardiner at 1058 and come back through the North Entrance at 2009.

I stop at Slough at 2111 to watch the fire burn along the ground in the sage and grass up by the yellow grass meadow and also see dead trees flaming on Anderson Ridge. No live trees seem to be burning, just grass and sage. There is a lot of flames.

I go east at 2133 and do not get any signals in Lamar.

I later hear that no wolves were seen in Hayden today.
