# Supplementary material for: Breeding displacement in gray wolves (Canis lupus): Three males usurp breeding position and pup rearing from a neighboring pack in Yellowstone National Park
Source: PLoS One. 2022 Nov 30;17(11):e0256618. doi: 10.1371/journal.pone.0256618 (PMC9710779; doi:10.1371/journal.pone.0256618)
Supplement: Attachment — Submitted filename: Plos_One_review_comments.docx [file pone.0256618.s003.docx]

Thanks for allowing me to review the manuscript of SundarRaj et al. on the breeding displacement of grey wolves in Yellowstone national park. The authors have quite detailed behavioural observations of the individuals from the two packs. Studies with such vivid details are quite rare and I thoroughly enjoyed reading the manuscript. I have a few comments and suggestions for the same that I have listed below.

**Introduction**

Line 56: Please provide a reference for the same

Line56-57: Please rewrite the sentence…do you mean ”thought to benefit”

Line 58-59: Reference required…advantageous for what…genetic diversity, pack stability? Can join the next sentence and rewrite.

Line 61: Should include the Latin name when you are mentioning the species for the first time

Line 71-73: Please separate the sentence into two sentences…difficult to follow

Line 76: Please refer to those studies here

Line 103-107: Please provide the details and legend of the background map Figure 1. Is it elevation? does it have any significance with the displacement, otherwise please use a white background.

**Methods**

The authors have provided quite detailed observations. It would be better if they prepare a table of the interactions between the dominant male and invaders with the outcome of the interaction. Sometimes it becomes a little difficult for the reader to keep track of the incidents over so many days.

Line 146: Are the wolves anaesthetized or they are immobilized?

Line 149: Please provide the details of the radio collars. Was it VHF or GPS and how frequently are they located on the ground?

**Discussion**

I have a few questions that were not discussed in the discussion section

Did the three young males attempt to displace or interact with any other wolf packs around?
Does the displacement only happen because of the proximity of these two packs?
Does the prey density and composition in these areas are homogeneous or a heterogeneous resource availability which may have led to a female mate choice in this study?
