# Supplementary material for: Breeding displacement in gray wolves (Canis lupus): Three males usurp breeding position and pup rearing from a neighboring pack in Yellowstone National Park
Source: PLoS One. 2022 Nov 30;17(11):e0256618. doi: 10.1371/journal.pone.0256618 (PMC9710779; doi:10.1371/journal.pone.0256618)
Supplement: Attachment — Submitted filename: Response to Reviewers.docx [file pone.0256618.s004.docx]

5 May 2022

To whom it may concern,

We appreciate the review of our manuscript PONE-D-21-25812” Breeding displacement in gray wolves (Canis lupus): Three males usurp breeding position and pup rearing from a neighboring pack in Yellowstone National Park.” We made nearly all of the recommended changes and explain all changes, additions, and deletions in detail below.

If you have any questions, please contact the lead author Jeremy SunderRaj at [j.sunderraj@hotmail.com](mailto:j.sunderraj@hotmail.com) or 303-945-5977.

Sincerely,

Jeremy SunderRaj

Editor Notes

1. The manuscript was reviewed and meets the style requirements of PLOS ONE. All figures were submitted as .tifs or .tiffs with associated titles and legends in the main text. All tables were included using standard word doc formatting.
2. We have attached the minimal dataset from the study as supporting information, containing detailed field notes collected in July 2016 and August 2016. These are listed in the supporting information section as S1 Fig and S2 Fig, respectively.
3. We added a separate section under the Methods which includes a full ethics statement outlining appropriate permitting/approval from the ethics committee (IACUC).
4. We attached a written statement from one of the coauthors, Kira Cassidy, who illustrated the images of the wolves in Figure 2, allowing for their use in the figure. We have also added her name to the figure caption.
5. We removed the base maps in Figures 1 and 3 to address copyright issues. Reviewer 2 also raised the concern that the base maps were confusing, and removing them also addressed this issue.

Reviewer 1

1. Reviewer 1’s first concern regarded how the presence of concerned wolves was evaluated using VHF receivers or GPS fixes. On **lines 154-157**, we added a description of the collar types used, both GPS and VHF, and included the approximate number of times wolves are located from the ground or the air via the VHF signals, which was around 5 times a week. We also added that the GPS collars typically take 4 fixes a day and upload points every 12 fixes.
2. The second concern from Reviewer 1 was the length of the daily observation summaries. We addressed this by adding a table of significant events on **lines 416-423** and limiting the daily observation summaries to aggressive encounters and notable events. We feel that the remaining day-to day observation descriptions are important to describe the unique nature and length of this particular displacement event.
3. Reviewer 1’s 3^rd^ concern regarded typographical errors in the manuscript. All coauthors have re-read the manuscript and have addressed typographical errors throughout.
4. In similar fashion to concern 3, Reviewer 1 suggested addressing unclear and over-explained language. All coauthors have re-read the manuscript and have made changes to address unclear and over-explained language.
5. Reviewer 1 suggested adding an additional table including key interactions during the displacement event described in the manuscript. We created and added this table on **lines 416-423**. This is also related to concern 2.
6. Reviewer 1 suggested adding additional information regarding the behavioral changes of the individual wolves involved in the displacement event, so we added a paragraph describing these changes in **lines 425-434.** This paragraph describes how the females quickly changed their behavior within the first week of the displacement from fleeing from the intruding males to displaying courtship behavior with them and successfully hunting with them, how the Wapiti Lake pups accepted the intruding males throughout the encounters, and how the interactions between 755M and the intruding Mollie’s males decreased in intensity toward the end of the displacement. Despite this, 755M still left the pack and dispersed east of Yellowstone.

Reviewer 2

1. Reviewer 1’s first concern was the lack of a citation following **line 56**, so we added a citation addressing the following statement: “In cases where male breeding status changes, females may stay with the male that they prefer, with mate selection likely coming from physical, genetic, behavioral, and situational cues.” We used a book chapter by Stahler et al. 2020, discussing wolf breeding strategies. The citation follows: Stahler DR, Smith DW, Cassidy KA, Stahler EE, Metz MC, McIntyre R, et al. Ecology of Family Dynamics in Yellowstone Wolf Packs. In: Smith DW, Stahler DR, MacNulty DR, editors. Yellowstone Wolves: Science and Discovery in the World’s First National Park. University of Chicago Press; 2020 Dec 21. pp. 42-45. https://doi.org/10.7208/9780226728483-011
2. Reviewer 2 suggested rewriting **line 56-58**, so we rewrote it to say “Mate preference is thought to benefit individuals most likely to bring the greatest direct and indirect fitness benefits.”
3. Review 2’s third concern regarded combining and rewriting **lines 58-61** and adding references, so we combined the sentences and included the importance of the benefits of pack size on stability and genetic diversity. References here include papers that found pack size is beneficial in hunting success (MacNulty et al. 2012), pup production and survival (Stahler et al. 2013), and success in conflict between packs (Cassidy et al. 2015). These studies suggest hunting success, pup production and survival, and success in territorial conflict lead to pack stability and genetic diversity, which is noted in the manuscript now.
4. We added the Latin name, *Canis lupus,* for the gray wolf on **line 62.**
5. Reviewer 2 suggested separating **lines 72-74**, so we separated them into two sentences for clarification.
6. Reviewer 2 suggested adding citations for captive wolf studies, so we added them on **line 78**, including Zimen 1976 and Packard et al. 1985. The citations, respectively, follow: 1.) Zimen E. On the regulation of pack size in wolves. Z Tierpsychol. 1976 Jan 12;40(3):300-41. https://doi.org/10.1111/j.1439-0310.1976.tb00939.x and 2.) Packard JM, Seal US, Mech LD, Plotka ED. Causes of reproductive failure in two family groups of wolves (Canis lupus). Z Tierpsychol. 1985 Jan 12;68(1):24-40. https://doi.org/10.1111/j.1439-0310.1985.tb00112.x
7. In response to Reviewer 2’s concern about confusion regarding the background maps in figures 1 and 3, we removed them and left the background white. This also addresses the concerns raised by the editor regarding copyright issues.
8. Reviewer 2 suggested adding a table of the interactions between the intruding males and 755M, so we added a table on **lines 416-423.** This table describes the interactions between the intruding males and 755M, and gives the results of each encounter. Toward the end of the displacement period, 755M’s reactions to the males chasing him changed from fleeing to bedding near them, and in the final case, following them. This did not stop him from leaving the pack and dispersing east of the park.
9. Reviewer 2 asked for clarification regarding whether captures wolves are immobilized or anesthetized, so we added that wolves are anesthetized using Telazol, which immobilizes them on **line 147.**
10. Reviewer 2 suggested adding a description of the collar types used and the frequency of location. We added on **lines 154-157** that collars are both GPS and VHF, and included the approximate number of times wolves are located from the ground or the air via the VHF signals, which is around 5 times a week. We also added that the GPS collars typically take 4 fixes a day an upload points every 12 fixes.
11. Reviewer two asked if the intruding males interacted with or displaced any other wolf packs in the area. The three intruding males did not interact with any additional wolf packs that we are aware of during the displacement period, and we added two sentences noting this in **lines 550-551.** The reasoning for this was likely that the intruding males found themselves in an ideal situation with the Wapiti Lake pack, finding several unrelated females to breed with and an older, smaller males who was easily displaced.
12. Reviewer 2 raised a question regarding whether the proximity of the Mollie’s pack and the Wapiti Lake pack was the only reason this displacement happened. We addressed while important, other factors also likely came into play in **lines 385-388.** Although the displacement certainly was impacted because of the proximity of the two packs, this likely was not the only reason. Other possibilities include the presence of a smaller sized breeding male, the presence of two unrelated females, and the fact that the intruding males outnumbered the breeding male of the Wapiti Lake pack 3 to 1. All of these factors likely contributed to the displacement.
13. Reviewer 2’s final question addressed whether prey density in the study was homogenous or heterogenous in composition and breakdown and whether that impacted the choice of the breeding female to remain in her territory with the intruding males. We addressed this in **lines 483-492.** In the summer, prey for wolves is typically homogenous in composition, consisting mostly of elk and bison. Density in Hayden Valley is consistent through the summer, but most prey migrate to lower elevations in the winter. This likely means that her choice to remain in her territory was likely not influenced by prey density and composition, but more likely the age of her pups and the added benefits of pack size that came with the intruding males.
