# Supplementary material for: Breeding displacement in gray wolves (Canis lupus): Three males usurp breeding position and pup rearing from a neighboring pack in Yellowstone National Park
Source: PLoS One. 2022 Nov 30;17(11):e0256618. doi: 10.1371/journal.pone.0256618 (PMC9710779; doi:10.1371/journal.pone.0256618)
Supplement: Attachment — Submitted filename: Response to Reviewers.docx [file pone.0256618.s005.docx]

10 October 2022

To whom it may concern,

We appreciate the review of our manuscript PONE-D-21-25812” Breeding displacement in gray wolves (Canis lupus): Three males usurp breeding position and pup rearing from a neighboring pack in Yellowstone National Park.” We made all of the recommended changes and explain all changes, additions, and deletions in detail below.

If you have any questions, please contact the lead author Jeremy SunderRaj at [j.sunderraj@hotmail.com](mailto:j.sunderraj@hotmail.com) or 303-945-5977.

Sincerely,

Jeremy SunderRaj

Editor Notes

1. We hyphenated the word interpack to inter-pack on **line 102.**
2. We changed the incorrect citation number from 9 to 10 on **line 141.**
3. We changed the incorrect citation number from 9 to 10 on **line 411.**
4. We hyphenated the word interpack to inter-pack on **line 471.**
5. We changed the incorrect citation number from 9 to 10 on **line 473.**
6. We changed the incorrect citation number from 8 to 9 on **line 478.**
7. We changed the incorrect citation number from 8 to 9 on **line 544.**
8. We changed the incorrect citation number from 9 to 10 on **line 553.**
9. We added acknowledgments for Joe Bump for recommendations on writing and figures and Wyoming Game and Fish staff members Ron Blanchard and Ken Mills for their assistance on **lines 576-579.**
